# Sunlight Induced and Recyclable g-C_3_N_4_ Catalyzed C-H Sulfenylation of Quinoxalin-2(1*H*)-Ones

**DOI:** 10.3390/molecules27155044

**Published:** 2022-08-08

**Authors:** Sha Peng, Jiao Liu, Li-Hua Yang, Long-Yong Xie

**Affiliations:** College of Chemistry and Bioengineering, Hunan University of Science and Engineering, Yongzhou 425100, China

**Keywords:** quinoxalin-2(1*H*)-ones, visible light, sulfenylation, heterogeneous photocatalyst, recyclable

## Abstract

A sunlight-promoted sulfenylation of quinoxalin-2(1*H*)-ones using recyclable graphitic carbon nitride (g-C_3_N_4_) as a heterogeneous photocatalyst was developed. Using the method, various 3-sulfenylated quinoxalin-2(1*H*)-ones were obtained in good to excellent yields under an ambient air atmosphere. Moreover, the heterogeneous catalyst can be recycled at least six times without significant loss of activity.

## 1. Introduction

**Scheme 1:**
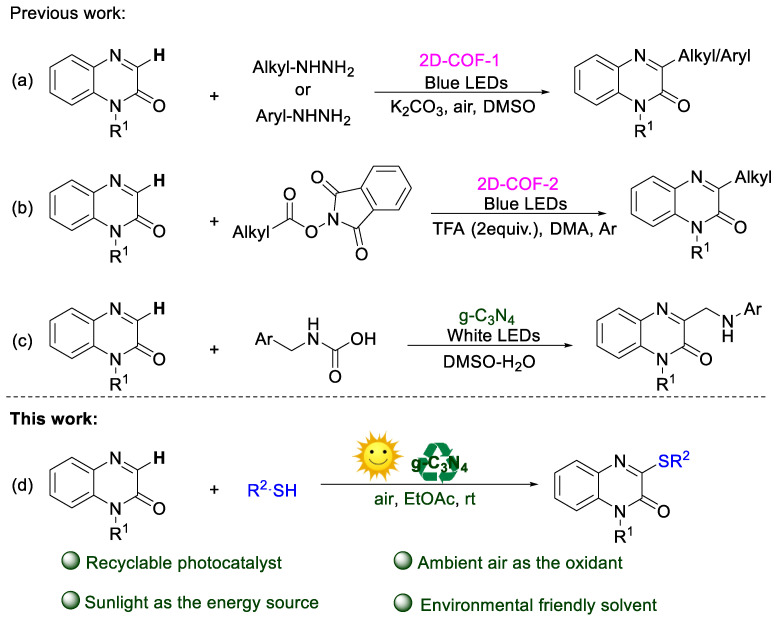
Heterogeneous photocatalyzed functionalization of quinoxalin-2(1H)-ones (**a**) 2D-COF-1 catalyzed alkylation/arylation of quinoxalin-2(1*H*)-ones; (**b**) 2D-COF-2 catalyzed alkylation of quinoxalin-2(1*H*)-ones; (**c**) g-C_3_N_4_ catalyzed hydroaminomethylation of quinoxalin-2(1*H*)-ones; (**d**) g-C_3_N_4_ catalyzed sulfenylation of quinoxalin-2(1*H*)-ones.

Quinoxalin-2(1*H*)-one is a privileged structural moiety, which exhibits various biological activities and pharmacological properties [1,2]. Consequently, a large number of 3-substituted quinoxalinones are prepared via direct C3–H functionalization of quinoxalin-2(1*H*)-ones in recent years, mainly including alkylation [3,4,5,6,7,8,9,10,11,12,13,14,15,16], arylation [17,18,19,20,21,22,23,24,25], acylation [26,27,28,29,30,31], alkoxylation [32,33,34,35], sulfenylation [36,37,38], amination [39,40,41,42,43,44], phosphonation [45,46,47,48,49] and trifluoromethylation [50,51,52,53]. Among them, photoredox catalysis has gained widespread concerns due to the unique advantages of energy-saving, high efficiency and handling simplicity [54,55,56,57]. However, most of the reported photocatalytic functionalization reactions are dominated by homogeneous photocatalysts, such as Ru(II) or Ir(III) based transition metal complexes or organic dyes, for example, Eosin Y, Rhodamine 6G, 4CzIPN and acridinium salts, whose photo properties are highlighted in the literature [58,59,60,61]. Although these homogenous photocatalysts show excellent photocatalytic activity in diverse reactions, they all encounter some common imperfections, including high economic and environmental cost, easy degradation/decomposition during the reaction, and poor reusability from the reaction system, which limit their large-scale and long-term use in pharmaceutical production. To address these issues, developing recyclable heterogeneous photocatalyzed transformation is an attractive and practical strategy. However, to date, only very limited examples of heterogeneous photocatalysis for the functionalization of quinoxalin-2(1*H*)-ones were reported. In 2019, Yang et al. developed visible-light-mediated arylation/alkylation reactions of quinoxalin-2(1*H*)-ones with hydrazines using a covalent organic framework (2D-COF-1) as a heterogeneous photocatalyst (Scheme 1a) [10]. Later, they further reported decarboxylative alkylation of quinoxalin-2(1*H*)-ones catalyzed by 2D-COF-2 under visible light irradiation (Scheme 1b) [62]. Despite these achievements, the utilization of heterogeneous photocatalyst for C-H functionalization of quinoxalin-2(1*H*)-ones is currently far from desired and of great significance.

As an abundant, clean and renewable energy source, sunlight has been wildly applied in various organic transformations. Many elegant sunlight-induced organic reactions are reported by Jiao [63], Wang [64], Pan [65], Zhu [66], Hashmi [67] and others [68,69,70,71]. With the increasing demand for green synthesis, the utilization of sunlight represents a hot topic of great interest. On the other hand, graphitic carbon nitride (g-C_3_N_4_) is an environmentally friendly, recyclable and inexpensive heterogeneous photocatalyst, which has emerged as a promising candidate to homogeneous photoredox catalysts [72]. Various novel g-C_3_N_4_ catalyzed photocatalytic reactions have been more deeply explored, such as controlled oxidation reactions [73,74,75], coupling reactions [76,77,78,79,80,81] and heterocyclizations [82,83,84]. To our knowledge, only Yu and co-workers demonstrated a visible light-induced g-C_3_N_4_-catalyzed decarboxylative reaction of quinoxalin-2(1*H*)-ones with N-aryl glycines (Scheme 1c) [84]. Therefore, developing more g-C_3_N_4_ catalyzed C3-H functionalization of quinoxalin-2(1*H*)-ones is in urgent demand.

As a part of our continuing interest in functionalized quinoxalines [30,37,85,86], herein, we wish to report sunlight induced and g-C_3_N_4_ catalyzed sulfenylation of quinoxalin-2(1*H*)-ones under air conditions (Scheme 1d). The current reaction provides a highly attractive and practical approach to selectively access various 3-sulfenylated quinoxalin-2(1*H*)-ones in good to excellent yields. Furthermore, the heterogeneous catalyst can be easily recycled up to six times, while maintaining its high catalytic activity. 

## 2. Results and Discussion

In our initial investigation, a template reaction of 1-methylquinoxalin-2(1*H*)-one (**1a**) and propane-2-thiol (**2a**) was performed to screen the reaction conditions (Table 1). Treatment of **1a** and **2a** with g-C_3_N_4_ (10 mg) in THF under 6w blue LED irradiation (450–455nm) in air for 12 h afforded **3aa** in 72% yield (entry 1). Screening of common organic solvents (entries 2–7) revealed that EtOAc was more efficient for the sulfenylation reaction (entry 4) and no reaction, occurred in water (entry 7), the light sources were also investigated (entries 8–11), to our delight, compared to blue, green, purple and white light sources, sunlight led to a better yield of **3aa** (entry 8). Besides, increasing the loading of g-C_3_N_4_ from 10 mg to 15 mg (entry 8 vs. entry 12), no better result was achieved, while decreasing the loading of g-C_3_N_4_ to 5 mg gave a lower yield of **3aa** (entry 8 vs. entry 13). When the reaction was conducted under N_2_ atmosphere or dark conditions (entries 14, 15), no reaction occurred. Furthermore, in the absence of a photocatalyst, **3aa** was also not observed (entry 16).

**Scheme 2:**
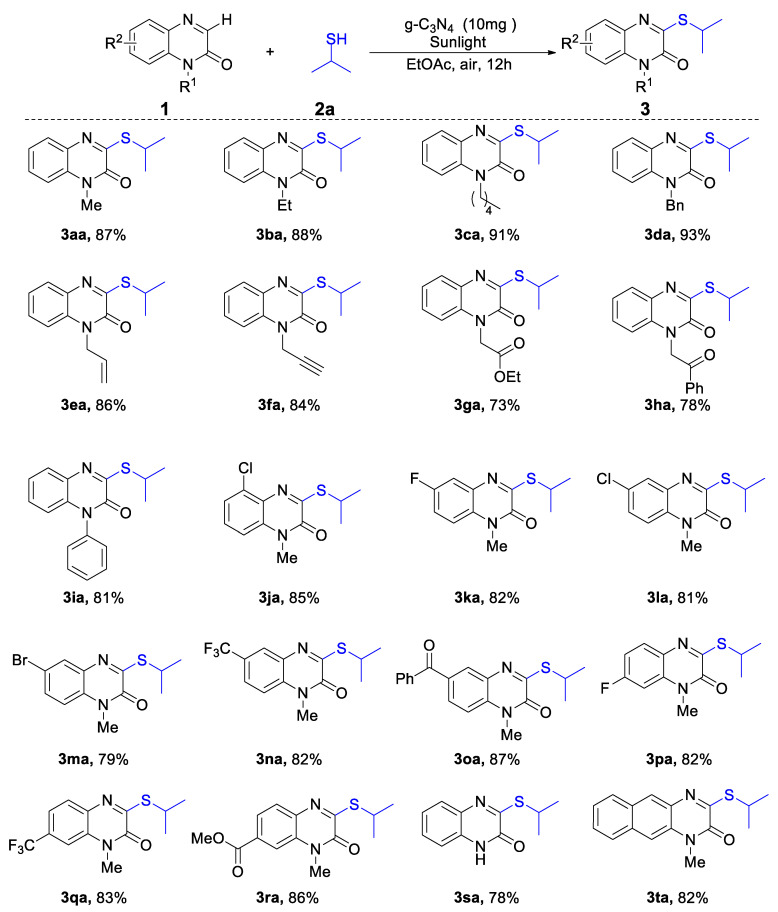
Preparation of **3aa**–**3ta**. Conditions: 1 (0.3 mmol), **2a** (0.9 mmol), g-C_3_N_4_ (10 mg), EtOAc (1.5 mL), sunlight, air, rt, 8h. Isolated yields were given.

After identifying the optimized reaction conditions (Table 1, entry 8), the substrate scope was firstly explored by employing different quinoxalin-2(1*H*)-ones (**1a**–**1t**) with propane-2-thiol (**2a**). As revealed in Scheme 2, *N*-substituted quinoxalinones bearing various alkyl or phenyl group reacted smoothly with propane-2-thiol **(2a)**, affording the corresponding products (**3aa**–**3ia**) with excellent yields. Furthermore, quinoxalin-2(1*H*)-ones containing diverse substituents on the phenyl ring could efficiently generate the desired products (**3ja**–**3ra**). Some important functional-groups, such as F (**3ka** and **3pa**), Cl (**3ja** and **3la**), Br (**3ma**), CF_3_ (**3na** and **3qa**), benzoyl (**3oa**) and ester (**3ra**) groups at different positions of aromatic rings were well compatible, providing a handle for post-transformations. Besides, *N*-unprotected quinoxalinone also reacted well to afford **3sa** in 78% isolated yield. Furthermore, 1-methylbenzo[g]quinoxalin-2(1*H*)-one also reacted well to give **3ta** in 82% yield.

**Scheme 3:**
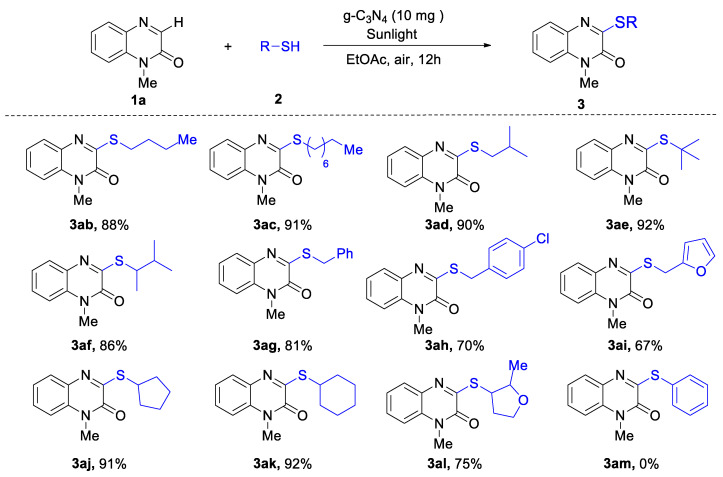
Preparation of **3ab**–**3am**. Conditions: **1a** (0.3 mmol), **2** (0.9 mmol), g-C_3_N_4_ (10 mg), EtOAc (1.5 mL), sunlight, air, rt, 8h. Isolated yields were given.

Next, we investigated the substrate generality with respect to thiols as evaluated in Scheme 3, various thiols (**2b**–**2m**) charged with different aliphatic chains and steric branched chains reacted smoothly to deliver products **3ab**–**3af** in excellent yields. Other linear thiols bearing a phenyl ring or a furan group also proceeded well to provide **3ag**–**3ai** in good yields. In addition, diverse cyclic substituted thiols were all compatible with the reaction, respectively, giving **3aj**–**3al** in good yields. Unfortunately, thiophenol (**2m**) failed to give the desired product (**3am**) and a remarkable dimerization product 1,2-diphenyldisulfane was detected in the reaction mixture.

**Scheme 4:**
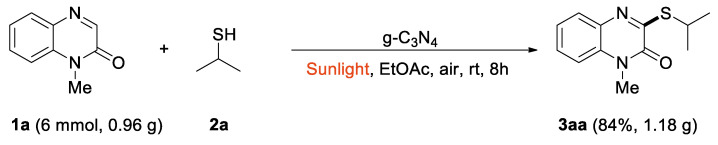
Gram-scale Synthesis of **3aa**.

To illustrate the synthetic application, a gram-scale experiment between **1a** and **2a** was carried out (Scheme 4). As anticipated, when the reaction was scaled up to 6 mmol, **3aa** was obtained in 84% isolated yield, suggesting the current reaction is a practical method for the synthesis of 3-thioquinoxalinones.

**Figure 1:**
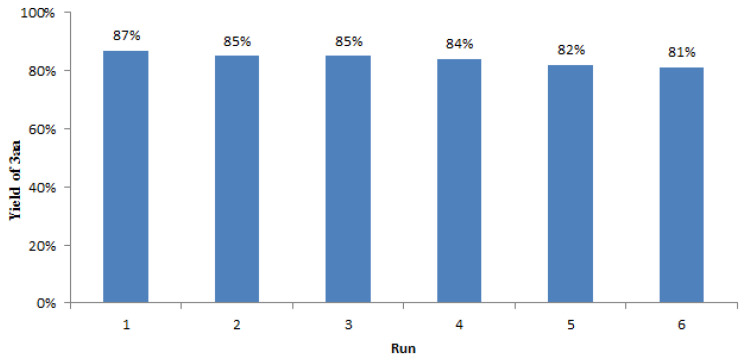
Catalyst recycling experiments.

Recycling studies were performed for the reaction between **1a** and **2a** under the standard conditions. After the reaction was complete, the g-C_3_N_4_ catalyst was recycled from the reaction mixture by simple filtration and rinsing with reaction solvent. The recovered photocatalyst was dried and then directly reused in the next round. As shown in Figure 1, the reaction was repeated up to six times, and no obvious losses in its catalytic activity were observed.

**Scheme 5:**
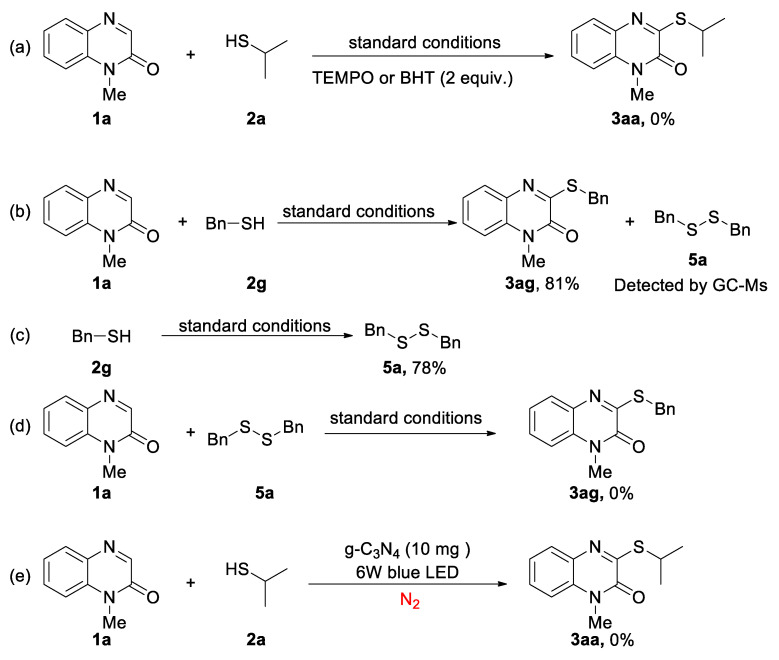
Control experiments. (**a**) Radical inhibition experiment using TEMPO or BHT as radical inhibitor; (**b**) Detection of product **5a** via GC-Ms; (**c**) Dimerization of phenylmethanethiol **2g**; (**d**) Reaction between **1a** and **5a** under standard conditions; (**e**) Reaction between **1a** and **2a** under N_2_ atmosphere.

To better understand the mechanism, some control experiments were performed (Scheme 5). The reaction was completely suppressed by addition of two equiv. of TEMPO or BHT (Scheme 5a), suggesting that radical intermediates might be involved in this transformation. Conducting the reaction using phenylmethanethiol **2g** as a substrate under the standard conditions, **3ag** was isolated in 81% yield and the 1,2- dibenzyldisulfane **5a** was detected by GC-MS (Scheme 5b). In addition, in the absence of 1-methylquinoxalin-2(1*H*)-one **1a**, phenylmethanethiol **2g** underwent a quick dimerization to generate **5a** in 78% yield (Scheme 5c). To confirm whether disulfides participate in the sulfenylation process, the reaction between **1a** and **5a** was performed, and no product **3ag** was detected (Scheme 5d), indicating that disulfides should not be the effective intermediates for the sulfenylation. Moreover, performing the template reaction under N_2_ atmosphere (Scheme 5e), no product **3aa** was observed, which demonstrates that dioxygen was crucial for the present transformation. 

**Scheme 6:**
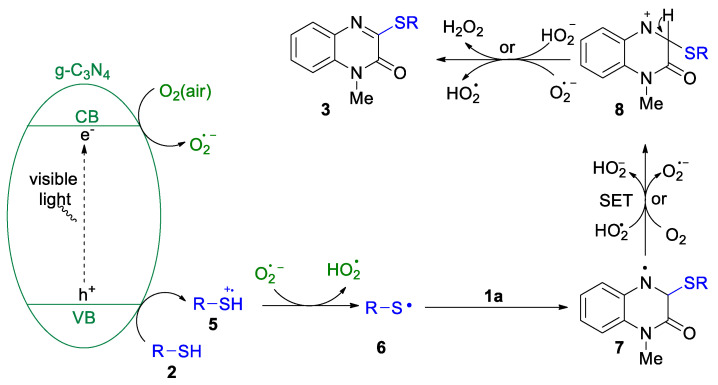
Possible mechanism.

Based on the above control experiment and related precedents in the literature [36,37,38], a possible reaction mechanism is proposed (Scheme 6). Initially, under the irradiation of visible light, g-C_3_N_4_ is excited and generates holes in the valence band (VB) and electrons in the conduction band (CB). Then, the holes obtain an electron from thiol **2** to generate thiyl radical cation **5** via a single electron transfer (SET) process. Simultaneously, the electrons in the CB were transferred to O_2_ (air) to generate O_2_^•−^. Next, O_2_^•−^ abstracted hydrogen from thiyl radical cation **5** to form HO_2_• species and thiyl radical **6**, which would add to C=N of **1a** giving nitrogen radical intermediate **7**. Intermediate **7** undergoes a further oxidative process by HO_2_• or O_2_ giving the intermediate **8**. Finally, the deprotonation of nitrogen cation intermediate **7** affords product **3**. 

## 3. Experimental Section

### 3.1. General Information

Unless otherwise noted, all reagents and solvents were used as received from commercial suppliers. The ^1^H, ^13^C and ^19^F NMR spectra were recorded at 400, 100 and 376 MHz by using a German Bruker Avance spectrometer. Chemical shifts were calibrated using residual undeuterated CDCl_3_ as an internal reference (^1^H NMR is calibrated at 7.26 ppm and ^13^C NMR at 77.0 ppm). Mass spectra were performed on a spectrometer operating on ESI-TOF. The catalyst g-C_3_N_4_ was purchased from JiangSu XFNANO Materials Tech Co., Ltd. (JiangSu, China).

### 3.2. General Procedure for the Preparation of 3-Thioquinoxalinones

A glass tube equipped with a magnetic stirrer bar was charged with quinoxalin-2(1*H*)-ones 1 (0.3 mmol), thiols 2 (0.9 mmol), g-C_3_N_4_ (10 mg) and EtOAc (1.5 mL). The reaction mixture was open to the air and stirred at room temperature under the irradiation of sunlight (sunny or cloudy weather) for about 8 h. After completion of the reaction, g-C_3_N_4_ was filtered out of the mixture. Then filtrate was extracted three times with ethyl acetate (5 mL × 3). The combined organic layers were dried over anhydrous Na_2_SO_4_. After filtration, the solvent was evaporated in vacuo. The crude product was purified by silica gel chromatography (petroleum ether/ethyl acetate = 10/1–6/1) to give the desired products **3**.

### 3.3. Gram-Scale Synthesis of ***3aa***

A glass tube equipped with a magnetic stirrer bar was charged with quinoxalin-2(1*H*)-ones **1a** (0.96 g, 6 mmol), pane-2-thiol **2a** (1.37 g, 18 mmol), g-C_3_N_4_ (200 mg) and EtOAc (30 mL). The reaction mixture was open to the air and stirred at room temperature under the irradiation of sunlight for about 8h. After completion of the reaction, g-C_3_N_4_ was filtered out of the mixture. Then filtrate was extracted three times with ethyl acetate (30 mL× 2). The combined organic layers were dried over anhydrous Na_2_SO_4_. After filtration, the solvent was evaporated in vacuo. The crude product was purified by silica gel chromatography (petroleum ether/ethyl acetate = 8/1) to give 1.18 g of **3aa**, yield 84%.

### 3.4. Recycling Experiments

A glass tube equipped with a magnetic stirrer bar was charged with quinoxalin-2(1*H*)-ones **1a** (0.048 g, 0.3 mmol), pane-2-thiol **2a** (0.068 g, 0.9 mmol), g-C_3_N_4_ (10 mg) and EtOAc (1.5 mL). The reaction mixture was open to the air and stirred at room temperature under the irradiation of sunlight for about 8h. After completion of the reaction, the g-C_3_N_4_ previously used was simply filtered and washed with EtOAc (2 mL), and then the recyclable g-C_3_N_4_ was dried under vacuum and directly reused for the next reaction cycle without any further purification. The yield of product **3aa** could be measured by ^1^H NMR using diethyl phthalate as an internal standard. (See Supplementary Materials)

### 3.5. Characterization Data of Products ***3aa–3ta*** and ***3ab–3al***

3-(isopropylthio)-1-methylquinoxalin-2(1*H*)-one (**3aa**):

White solid; mp 115–117 °C; 61.1 mg (isolated yield 87%); ^1^H NMR (400 MHz, Chloroform-*d*) δ 7.74 (d, *J* = 8.0 Hz, 1H), 7.48–7.41 (m, 1H), 7.34–7.26 (m, 2H), 4.03–3.96 (m, 1H), 3.69 (s, 3H), 1.45 (d, *J* = 6.9 Hz, 6H); ^13^C NMR (100 MHz, Chloroform-*d*) δ 160.0, 153.3, 133.5, 131.2, 128.2, 128.0, 123.7, 113.7, 34.4, 29.2, 22.5; HRMS (ESI): *m*/*z* [M×H]^+^ calcd for C_1__2_H_1__5_N_2_OS: 235.0900; found: 235.0902. The compound spectra data is in agreement with the report [87].

ethyl-3-(isopropylthio)quinoxalin-2(1*H*)-one (**3ba**):

Colorless liquid; 65.5 mg (isolated yield 88%); ^1^H NMR (400 MHz, Chloroform-*d*) δ 7.75 (d, *J* = 7.7 Hz, 1H), 7.44 (t, *J* = 7.8 Hz, 1H), 7.30 (d, *J* = 7.8 Hz, 2H), 4.31 (q, *J* = 7.2 Hz, 2H), 4.03–3.96 (m, 1H), 1.45 (d, *J* = 6.9 Hz, 6H), 1.37 (t, *J* = 7.2 Hz, 3H); ^13^C NMR (100 MHz, Chloroform-*d*) δ 160.0, 152.7, 133.9, 130.1, 128.5, 128.0, 123.5, 113.5, 37.4, 34.4, 22.5, 12.3; HRMS (ESI): *m*/*z* [M+H]^+^ calcd for C_13_H_17_N_2_OS: 249.1056; found: 249.1063.

3-(isopropylthio)-1-pentylquinoxalin-2(1*H*)-one (**3ca**):

Colorless liquid; 79.2 mg (isolated yield 91%); ^1^H NMR (400 MHz, Chloroform-d) δ 7.75 (d, *J* = 8.0 Hz, 1H), 7.48–7.39 (m, 1H), 7.29 (t, *J* = 8.1 Hz, 2H), 4.30–4.18 (m, 2H), 4.04–3.93 (m, 1H), 1.80–1.71 (m, 2H), 1.46 (d, *J* = 6.9 Hz, 6H), 1.44–1.32 (m, 4H), 0.91 (t, *J* = 7.0 Hz, 3H); 13C NMR (100 MHz, Chloroform-d) δ 160.0, 153.0, 133.8, 130.4, 128.5, 127.9, 123.5, 113.7, 42.5, 34.4, 29.0, 26.8, 22.5, 22.3, 13.9; HRMS (ESI): *m*/*z* [M+H]^+^ calcd for C16H23N2OS: 291.1526; found: 291.1529.

benzyl-3-(isopropylthio)quinoxalin-2(1*H*)-one (**3da**):

White solid; mp 123–125 °C; 86.5 mg (isolated yield 93%); ^1^H NMR (400 MHz, Chloroform-*d*) δ 7.76 (dd, *J* = 7.7, 1.7 Hz, 1H), 7.35–7.22 (m, 8H), 5.49 (s, 2H), 4.07–4.00 (m, 1H), 1.49 (d, *J* = 6.9 Hz, 6H); ^13^C NMR (100 MHz, Chloroform-*d*) δ 160.1, 153.4, 135.1, 133.8, 130.5, 128.8, 128.3, 128.0, 127.7, 127.0, 123.8, 114.5, 46.1, 34.6, 22.6; HRMS (ESI): *m*/*z* [M+H]^+^ calcd for C_18_H_19_N_2_OS: 311.1213; found: 311.1218.

allyl-3-(isopropylthio)quinoxalin-2(1*H*)-one (**3ea**):

Colorless liquid; 67.1 mg (isolated yield 86%); ^1^H NMR (400 MHz, Chloroform-*d*) δ 7.76 (d, *J* = 7.8 Hz, 1H), 7.41 (t, *J* = 7.4 Hz, 1H), 7.34–7.25 (m, 2H), 5.92 (ddt, *J* = 15.8, 10.4, 5.2 Hz, 1H), 5.23 (dd, *J* = 28.4, 13.8 Hz, 2H), 4.90 (d, *J* = 5.1 Hz, 2H), 4.04–3.97 (m, 1H), 1.47 (d, *J* = 6.9 Hz, 6H); ^13^C NMR (100 MHz, Chloroform-*d*) δ 160.0, 152.9, 133.7, 130.5, 130.4, 128.3, 128.0, 123.7, 118.3, 114.3, 44.7, 34.5, 22.5; HRMS (ESI): *m*/*z* [M+H]^+^ calcd for C_14_H_17_N_2_OS: 261.1056; found: 261.1052.

3-(isopropylthio)-1-(prop-2-yn-1-yl)quinolin-2(1*H*)-one (**3fa**):

White solid; mp 172–174 °C; 65.0 mg (isolated yield 84%); ^1^H NMR (400 MHz, Chloroform-*d*) δ 7.76 (d, *J* = 8.7 Hz, 1H), 7.52–7.41 (m, 2H), 7.37–7.30 (m, 1H), 5.04 (d, *J* = 2.5 Hz, 2H), 4.03–3.96 (m, 1H), 2.28 (t, *J* = 2.5 Hz, 1H), 1.46 (d, *J* = 6.9 Hz, 6H); ^13^C NMR (100 MHz, Chloroform-*d*) δ 159.8, 152.3, 133.7, 129.7, 128.3, 128.1, 124.1, 114.2, 76.6, 73.3, 34.6, 31.6, 22.5; HRMS (ESI): *m*/*z* [M+H]^+^ calcd for C_14_H_15_N_2_OS: 259.0900; found: 259.0908.

ethyl 2-(3-(isopropylthio)-2-oxoquinoxalin-1(2*H*)-yl)acetate (**3ga**):

Colorless liquid; 67.0 mg (isolated yield 73%); ^1^H NMR (400 MHz, Chloroform-*d*) δ 7.82–7.71 (m, 1H), 7.47–7.36 (m, 1H), 7.31 (t, *J* = 7.5 Hz, 1H), 7.04 (d, *J* = 8.2 Hz, 1H), 5.01 (s, 2H), 4.23 (q, *J* = 7.1 Hz, 2H), 4.05–3.98 (m, 1H), 1.47 (d, *J* = 6.9 Hz, 6H), 1.26 (t, *J* = 7.1 Hz, 3H); ^13^C NMR (100 MHz, Chloroform-*d*) δ 166.9, 159.7, 152.9, 133.6, 130.4, 128.5, 128.2, 124.0, 113.2, 62.0, 43.6, 34.6, 22.5, 14.1; HRMS (ESI): *m*/*z* [M+H]^+^ calcd for C_15_H_19_N_2_O_3_S: 307.1111; found: 307.1107.

3-(isopropylthio)-1-(2-oxo-2-phenylethyl)quinoxalin-2(1*H*)-one (**3ha**):

White solid; mp 184–186 °C; 79.1 mg (isolated yield 78%); ^1^H NMR (400 MHz, Chloroform-*d*) δ 8.05 (d, *J* = 7.5 Hz, 2H), 7.77 (dd, *J* = 7.5, 1.8 Hz, 1H), 7.65 (t, *J* = 7.4 Hz, 1H), 7.53 (t, *J* = 8.0 Hz, 2H), 7.36–7.22 (m, 2H), 6.92 (d, *J* = 9.1 Hz, 1H), 5.72 (s, 2H), 4.06–3.99 (m, 1H), 1.47 (d, *J* = 6.9 Hz, 6H); ^13^C NMR (100 MHz, Chloroform-*d*) δ 190.9, 159.5, 153.1, 134.5, 134.2, 133.7, 130.6, 129.0, 128.5, 128.1, 128.1, 123.8, 113.6, 48.5, 34.5, 22.5; HRMS (ESI): *m*/*z* [M+H]^+^ calcd for C_19_H_19_N_2_O_2_S: 339.1162; found: 339.1164.

3-(isopropylthio)-1-phenylquinoxalin-2(1*H*)-one (**3ia**):

White solid; mp 153–155 °C; 71.9 mg (isolated yield 81%); ^1^H NMR (400 MHz, Chloroform-*d*) δ 7.79 (d, *J* = 7.8 Hz, 1H), 7.68–7.50 (m, 3H), 7.32–7.26 (m, 3H), 7.23 (t, *J* = 7.0 Hz, 1H), 6.65 (d, *J* = 8.1 Hz, 1H), 4.12–3.97 (m, 1H), 1.50 (d, *J* = 6.9 Hz, 6H); ^13^C NMR (100 MHz, Chloroform-*d*) δ 160.7, 152.9, 135.6, 133.4, 132.2, 130.2, 129.4, 128.3, 127.9, 127.7, 123.9, 115.6, 34.6, 22.6; HRMS (ESI): *m*/*z* [M+H]^+^ calcd for C_17_H_17_N_2_OS: 297.1056; found: 297.1051.

5-chloro-3-(isopropylthio)-1-methylquinoxalin-2(1*H*)-one (**3ja**):

White solid; mp 126–128 °C; 68.3 mg (isolated yield 85%); ^1^H NMR (400 MHz, Chloroform-*d*) δ 7.44–7.39 (m, 1H), 7.34 (t, *J* = 8.1 Hz, 1H), 7.20 (d, *J* = 8.3 Hz, 1H), 4.09–4.02 (m, 1H), 3.69 (s, 3H), 1.50 (d, *J* = 6.8 Hz, 6H); ^13^C NMR (100 MHz, Chloroform-*d*) δ 161.0, 153.0, 133.0, 132.7, 130.1, 127.8, 124.6, 112.5, 35.4, 29.7, 22.2; HRMS (ESI): *m*/*z* [M+H]^+^ calcd for C_12_H_14_ClN_2_OS: 269.0510; found: 269.0513.

6-fluoro-3-(isopropylthio)-1-methylquinoxalin-2(1*H*)-one (**3ka**):

White solid; mp 174–176 °C; 62.0 mg (isolated yield 82%); ^1^H NMR (400 MHz, Chloroform-*d*) δ 7.42 (dd, *J* = 8.9, 2.8 Hz, 1H), 7.25–7.14 (m, 2H), 3.99–3.92 (m, 1H), 3.68 (s, 3H), 1.44 (d, *J* = 6.9 Hz, 6H); ^13^C NMR (100 MHz, Chloroform-*d*) δ 161.9, 158.9 (d, *J*_C-F_ = 244.4 Hz), 152.9, 134.1 (d, *J*_C-F_ = 12.1 Hz), 127.9 (d, *J*_C-F_ = 2.0 Hz), 115.5 (d, *J*_C-F_ = 24.2 Hz), 114.7 (d, *J*_C-F_ = 9.1 Hz), 113.7 (d, *J*_C-F_ = 22.2 Hz), 34.6, 29.5, 22.4; ^19^F NMR (376 MHz, Chloroform-*d*) δ −119.1; HRMS (ESI): *m*/*z* [M+H]^+^ calcd for C_12_H_14_FN_2_OS: 253.0805; found: 253.0804.

6-chloro-3-(isopropylthio)-1-methylquinolin-2(1*H*)-one (**3la**):

White solid; mp 127–129 °C; 65.1 mg (isolated yield 81%); ^1^H NMR (400 MHz, Chloroform-*d*) δ 7.73 (d, *J* = 2.4 Hz, 1H), 7.39 (dd, *J* = 8.9, 2.4 Hz, 1H), 7.20 (d, *J* = 8.9 Hz, 1H), 3.99–3.92 (m, 1H), 3.67 (s, 3H), 1.45 (d, *J* = 6.9 Hz, 6H); ^13^C NMR (100 MHz, Chloroform-*d*) δ 161.8, 153.0, 134.0, 130.0, 129.1, 127.9, 127.6, 114.8, 34.7, 29.4, 22.4; HRMS (ESI): *m*/*z* [M+H]^+^ calcd for C_12_H_14_ClN_2_OS: 269.0510; found: 269.0514.

6-bromo-3-(isopropylthio)-1-methylquinolin-2(1*H*)-one (**3ma**):

White solid; mp 144–146 °C; 73.9 mg (isolated yield 79%); ^1^H NMR (400 MHz, Chloroform-*d*) δ 7.89 (s, 1H), 7.52 (d, *J* = 11.0 Hz, 1H), 7.15 (d, *J* = 8.8 Hz, 1H), 3.99–3.92 (m, 1H), 3.67 (s, 3H), 1.44 (d, *J* = 6.9 Hz, 6H); ^13^C NMR (100 MHz, Chloroform-*d*) δ 161.7, 152.9, 134.3, 130.6, 130.6, 130.4, 116.4, 115.1, 34.7, 29.4, 22.4; HRMS (ESI): *m*/*z* [M+H]^+^ calcd for C_12_H_14_BrN_2_OS: 313.0005; found: 313.0003.

3-(isopropylthio)-1-methyl-6-(trifluoromethyl)quinolin-2(1*H*)-one (**3na**):

White solid; mp 115–117 °C; 74.3 mg (isolated yield 82%); ^1^H NMR (400 MHz, Chloroform-*d*) δ 8.01 (s, 1H), 7.66 (dd, *J* = 8.7, 1.6 Hz, 1H), 7.38 (d, *J* = 8.7 Hz, 1H), 4.03–3.96 (m, 1H), 3.72 (s, 3H), 1.46 (d, *J* = 6.9 Hz, 6H); ^13^C NMR (100 MHz, Chloroform-*d*) δ 162.1, 153.2, 133.6, 132.9, 126.3 (q, *J*_C-F_ = 33.3 Hz), 125.5 (q, *J*_C-F_ = 4.0 Hz), 124.3 (q, *J*_C-F_ = 4.0 Hz), 123.8 (q, *J*_C-F_ = 273.7 Hz), 114.3, 34.9, 29.5, 22.5; ^19^F NMR (376 MHz, Chloroform-*d*) δ -61.9; HRMS (ESI): *m*/*z* [M+H]^+^ calcd for C_13_H_14_F_3_N_2_OS: 303.0773; found: 303.0762.

6-benzoyl-3-(isopropylthio)-1-methylquinoxalin-2(1*H*)-one (**3oa**):

White solid; mp 172–174 °C; 88.2 mg (isolated yield 87%); ^1^H NMR (400 MHz, Chloroform-*d*) δ 8.17 (d, *J* = 1.9 Hz, 1H), 7.95 (dd, *J* = 8.6, 1.9 Hz, 1H), 7.82 (d, *J* = 7.3 Hz, 2H), 7.63 (t, *J* = 7.4 Hz, 1H), 7.52 (t, *J* = 7.6 Hz, 2H), 7.39 (d, *J* = 8.7 Hz, 1H), 4.01–3.95 (m, 1H), 3.74 (s, 3H), 1.44 (d, *J* = 6.9 Hz, 6H); ^13^C NMR (100 MHz, Chloroform-*d*) δ 195.2, 161.4, 153.2, 137.5, 134.4, 132.9, 132.7, 132.5, 130.5, 129.9, 129.4, 128.4, 113.9, 34.7, 29.6, 22.5; HRMS (ESI): *m*/*z* [M+H]^+^ calcd for C_19_H_19_N_2_OS: 339.1162; found: 339.1157.

7-fluoro-3-(isopropylthio)-1-methylquinolin-2(1*H*)-one (**3pa**):

White solid; mp 164–166 °C; 62.0 mg (isolated yield 82%); ^1^H NMR (400 MHz, Chloroform-*d*) δ 7.69 (dd, *J* = 8.8, 5.9 Hz, 1H), 7.10–6.84 (m, 2H), 3.98–3.91 (m, 1H), 3.64 (s, 3H), 1.44 (d, *J* = 6.9 Hz, 6H); ^13^C NMR (100 MHz, Chloroform-*d*) δ 161.8 (d, *J*_C-F_ = 248.5 Hz), 158.9, 153.1, 132.5 (d, *J*_C-F_ = 12.1 Hz), 130.3 (d, *J*_C-F_ = 2.0 Hz), 129.8 (d, *J*_C-F_ = 10.1 Hz), 111.2 (d, *J*_C-F_ = 23.2 Hz), 100.87 (d, *J*_C-F_ = 28.3 Hz), 34.5, 29.5, 22.5; ^19^F NMR (376 MHz, Chloroform-*d*) δ −110.3; HRMS (ESI): *m*/*z* [M+H]^+^ calcd for C_12_H_14_FN_2_OS: 253.0805; found: 253.0807.

3-(isopropylthio)-1-methyl-7-(trifluoromethyl)quinolin-2(1*H*)-one (**3qa**):

White solid; mp 121–123 °C; 75.2 mg (isolated yield 83%); ^1^H NMR (400 MHz, Chloroform-*d*) δ 7.83 (d, *J* = 8.3 Hz, 1H), 7.60–7.48 (m, 2H), 4.03–3.97 (m, 1H), 3.73 (s, 3H), 1.46 (d, *J* = 6.9 Hz, 6H); ^13^C NMR (100 MHz, Chloroform-*d*) δ 163.1, 153.0, 135.1 (q, *J*_C-F_ = 1.0 Hz), 131.3, 129.5 (q, *J*_C-F_ = 33.3 Hz), 128.7, 123.8 (q, *J*_C-F_ = 273.7 Hz), 120.3 (q, *J*_C-F_ = 4.0 Hz), 111.2 (q, *J*_C-F_ = 4.0 Hz), 34.8, 29.4, 22.4; ^19^F NMR (376 MHz, Chloroform-*d*) δ −62.0; HRMS (ESI): *m*/*z* [M+H]^+^ calcd for C_13_H_14_F_3_N_2_OS: 303.0773; found: 303.0782.

methyl 2-(isopropylthio)-4-methyl-3-oxo-3,4-dihydroquinoxaline-6-carboxylate (**3ra**):

White solid; mp 172–174 °C; 75.4 mg (isolated yield 86%); ^1^H NMR (400 MHz, Chloroform-*d*) δ 8.04–7.94 (m, 2H), 7.77 (d, *J* = 8.3 Hz, 1H), 4.06–3.99 (m, 1H), 3.97 (s, 3H), 3.75 (s, 3H), 1.46 (d, *J* = 6.9 Hz, 6H); ^13^C NMR (100 MHz, Chloroform-*d*) δ 166.3, 163.1, 153.1, 136.2, 131.1, 129.0, 128.1, 124.7, 115.6, 52.5, 34.8, 29.5, 22.4; HRMS (ESI): *m*/*z* [M+H]^+^ calcd for C_14_H_17_N_2_O_3_S: 293.0954; found: 293.0958.

3-(isopropylthio)quinoxalin-2(1*H*)-one (**3sa**):

White solid; mp 255–257 °C; 51.5 mg (isolated yield 78%); ^1^H NMR (400 MHz, Chloroform-*d*) δ 12.18 (s, 1H), 7.75 (d, *J* = 7.9 Hz, 1H), 7.45–7.36 (m, 2H), 7.35–7.28 (m, 1H), 4.10–4.03 (m, 1H), 1.49 (d, *J* = 6.8 Hz, 6H); ^13^C NMR (100 MHz, Chloroform-*d*) δ 159.7, 154.9, 133.6, 128.9, 128.2, 127.3, 124.4, 116.1, 34.5, 22.6; HRMS (ESI): *m*/*z* [M+H]^+^ calcd for C_1__1_H_1__3_N_2_OS: 221.0743; found: 221.0738.

3-(isopropylthio)-1-methylbenzo[g]quinoxalin-2(1*H*)-one (**3ta**):

White solid; mp 216–218 °C; 69.9 mg (isolated yield 82%); ^1^H NMR (400 MHz, Chloroform-*d*) δ 8.22 (s, 1H), 7.94 (d, *J* = 8.1 Hz, 1H), 7.89 (d, *J* = 8.2 Hz, 1H), 7.58 (s, 1H), 7.50 (dt, *J* = 23.6, 7.0 Hz, 2H), 4.08–4.01 (m, 1H), 3.76 (s, 3H), 1.50 (d, *J* = 6.9 Hz, 6H); ^13^C NMR (100 MHz, Chloroform-*d*) δ 160.5, 153.2, 132.7, 132.4, 130.5, 130.0, 128.0, 127.2, 127.0, 126.5, 125.3, 110.2, 34.7, 29.3, 22.6; HRMS (ESI): *m*/*z* [M+H]^+^ calcd for C_16_H_17_N_2_OS: 285.1056; found: 285.1059.

3-(butylthio)-1-methylquinoxalin-2(1*H*)-one (**3ab**):

White solid; mp 112–114 °C; 65.5 mg (isolated yield 88%); ^1^H NMR (400 MHz, Chloroform-*d*) δ 7.75 (d, *J* = 7.9 Hz, 1H), 7.45 (t, *J* = 7.7 Hz, 1H), 7.36–7.26 (m, 2H), 3.71 (s, 3H), 3.18 (t, *J* = 7.3 Hz, 2H), 1.78–1.70 (m, 2H), 1.56–1.47 (m, 2H), 0.97 (t, *J* = 7.3 Hz, 3H); ^13^C NMR (100 MHz, Chloroform-*d*) δ 160.1, 153.4, 133.5, 131.3, 128.2, 128.1, 123.8, 113.7, 30.6, 29.2, 22.1, 13.7; HRMS (ESI): *m*/*z* [M+H]^+^ calcd for C_1__3_H_17_N_2_OS: 249.1056; found: 249.1052. The compound spectra data is in agreement with the report [37].

1-methyl-3-(octylthio)quinoxalin-2(1*H*)-one (**3ac**):

White solid; mp 124–126 °C; 83.0 mg (isolated yield 91%); ^1^H NMR (400 MHz, Chloroform-*d*) δ 7.79–7.70 (m, 1H), 7.49–7.39 (m, 1H), 7.35–7.24 (m, 2H), 3.69 (s, 3H), 3.16 (t, *J* = 7.4 Hz, 2H), 1.78–1.71 (m, 2H), 1.54–1.44 (m, 2H), 1.37–1.28 (m, 8H), 0.88 (t, *J* = 6.7 Hz, 3H); ^13^C NMR (100 MHz, Chloroform-*d*) δ 160.1, 153.3, 133.4, 131.3, 128.1, 128.0, 123.7, 113.6, 31.8, 29.5, 29.2, 29.1, 29.1, 29.0, 28.5, 22.6, 14.0; HRMS (ESI): *m*/*z* [M+H]^+^ calcd for C_1__7_H_25_N_2_OS: 305.1682; found: 305.1681. The compound spectra data is in agreement with the report [37].

3-(isobutylthio)-1-methylquinoxalin-2(1*H*)-one (**3ad**):

White solid; mp 117–119 °C; 67.0 mg (isolated yield 90%); ^1^H NMR (400 MHz, Chloroform-*d*) δ 7.73 (d, *J* = 8.0 Hz, 1H), 7.47–7.40 (m, 1H), 7.34–7.25 (m, 2H), 3.69 (s, 3H), 3.09 (d, *J* = 6.7 Hz, 2H), 2.06–1.99 (m, 1H), 1.07 (d, *J* = 6.7 Hz, 6H); ^13^C NMR (100 MHz, Chloroform-*d*) δ 160.1, 153.4, 133.4, 131.3, 128.2, 128.0, 123.7, 113.7, 38.0, 29.2, 28.1, 22.1; HRMS (ESI): *m*/*z* [M+H]^+^ calcd for C_1__3_H_17_N_2_OS: 249.1056; found: 249.1052. The compound spectra data is in agreement with the report [87].

3-(tert-butylthio)-1-methylquinoxalin-2(1*H*)-one (**3ae**):

White solid; mp 106–108 °C; 68.5 mg (isolated yield 92%); ^1^H NMR (400 MHz, Chloroform-*d*) δ 7.74 (dd, *J* = 7.9, 1.3 Hz, 1H), 7.46–7.40 (m, 1H), 7.33–7.24 (m, 2H), 3.67 (s, 3H), 1.67 (s, 9H); ^13^C NMR (100 MHz, Chloroform-*d*) δ 160.5, 153.2, 133.1, 131.1, 128.2, 128.0, 123.6, 113.6, 47.1, 29.5, 29.2; HRMS (ESI): *m*/*z* [M+H]^+^ calcd for C_1__3_H_17_N_2_OS: 249.1056; found: 249.1058. The compound spectra data is in agreement with the report [87].

1-methyl-3-((3-methylbutan-2-yl)thio)quinoxalin-2(1*H*)-one (**3af**):

White solid; mp 114–116 °C; 67.6 mg (isolated yield 86%); ^1^H NMR (400 MHz, Chloroform-*d*) δ 7.71 (d, *J* = 8.6 Hz, 1H), 7.41 (t, *J* = 7.8 Hz, 1H), 7.31–7.24 (m, 2H), 4.00–3.92 (m, 1H), 3.67 (s, 3H), 2.11–2.00 (m, 1H), 1.37 (d, *J* = 7.0 Hz, 3H), 1.06 (d, *J* = 8.0, 3H), 1.03 (d, *J* = 8.0, 3H); ^13^C NMR (100 MHz, Chloroform-*d*) δ 159.9, 153.3, 133.4, 131.2, 128.0, 127.9, 123.6, 113.6, 45.2, 32.6, 19.7, 19.2, 17.2; HRMS (ESI): *m*/*z* [M+H]^+^ calcd for C_1__4_H_19_N_2_OS: 263.1213; found: 263.1217. The compound spectra data is in agreement with the report [37].

3-(benzylthio)-1-methylquinoxalin-2(1*H*)-one (**3ag**):

White solid; mp 137–139 °C; 68.6 mg (isolated yield 81%); ^1^H NMR (400 MHz, Chloroform-*d*) δ 7.79 (d, *J* = 7.7 Hz, 1H), 7.45 (t, *J* = 6.9 Hz, 3H), 7.37–7.20 (m, 5H), 4.41 (s, 2H), 3.68 (s, 3H); ^13^C NMR (100 MHz, Chloroform-*d*) δ 159.3, 153.2, 137.3, 133.4, 131.5, 129.3, 128.4, 128.3, 128.2, 127.1, 123.9, 113.8, 34.0, 29.2; HRMS (ESI): *m*/*z* [M+H]^+^ calcd for C_16_H_15_N_2_OS: 283.0900; found: 283.0897. The compound spectra data is in agreement with the report [37].

3-((4-chlorobenzyl)thio)-1-methylquinoxalin-2(1*H*)-one (**3ah**):

White solid; mp 148–150 °C; 66.4 mg (isolated yield 70%); ^1^H NMR (400 MHz, Chloroform-*d*) δ 7.78 (dd, *J* = 7.9, 1.2 Hz, 1H), 7.51–7.44 (m, 1H), 7.40 (d, *J* = 8.4 Hz, 2H), 7.37–7.28 (m, 2H), 7.26–7.22 (m, 2H), 4.36 (s, 2H), 3.70 (s, 3H); ^13^C NMR (100 MHz, Chloroform-*d*) δ 159.0, 153.2, 136.0, 133.3, 132.9, 131.5, 130.6, 128.5, 128.2, 124.0, 113.8, 33.2, 29.3; HRMS (ESI): *m*/*z* [M+H]^+^ calcd for C_1__6_H_14_ClN_2_OS: 317.0510; found: 317.0516. The compound spectra data is in agreement with the report [37].

3-((furan-2-ylmethyl)thio)-1-methylquinoxalin-2(1*H*)-one (**3ai**):

White solid; mp 124–126 °C; 54.7 mg (isolated yield 67%); ^1^H NMR (400 MHz, Chloroform-*d*) δ 7.81 (d, *J* = 7.9 Hz, 1H), 7.48 (t, *J* = 7.8 Hz, 1H), 7.39–7.27 (m, 3H), 6.35–6.26 (m, 2H), 4.46 (s, 2H), 3.70 (s, 3H); ^13^C NMR (100 MHz, Chloroform-*d*) δ 158.8, 153.2, 150.5, 142.0, 133.3, 131.5, 128.5, 128.3, 123.9, 113.8, 110.5, 108.1, 29.2, 26.4; HRMS (ESI): *m*/*z* [M+H]^+^ calcd for C_1__4_H_13_N_2_O_2_S: 273.0692; found: 273.0688. The compound spectra data is in agreement with the report [37].

3-(cyclopentylthio)-1-methylquinoxalin-2(1*H*)-one (**3aj**):

White solid; mp 113–115 °C; 71.0 mg (isolated yield 91%); ^1^H NMR (400 MHz, Chloroform-*d*) δ 7.81–7.70 (m, 1H), 7.47–7.40 (m, 1H), 7.31 (d, *J* = 8.0, 1H), 7.27 (d, *J* = 8.0, 1H), 4.08–4.01 (m, 1H), 3.69 (s, 3H), 2.28 (q, *J* = 9.1, 7.1 Hz, 2H), 1.81–1.63 (m, 6H); ^13^C NMR (100 MHz, Chloroform-*d*) δ 160.8, 153.3, 133.6, 131.3, 128.3, 128.0, 123.7, 113.7, 42.5, 33.0, 29.2, 25.0; HRMS (ESI): *m*/*z* [M+H]^+^ calcd for C_1__4_H_17_N_2_OS: 261.1056; found: 261.1055. The compound spectra data is in agreement with the report [37].

3-(cyclohexylthio)-1-methylquinoxalin-2(1*H*)-one (**3ak**):

White solid; mp 116–118 °C; 75.7 mg (isolated yield 92%); ^1^H NMR (400 MHz, Chloroform-*d*) δ 7.73 (dd, *J* = 7.9, 1.2 Hz, 1H), 7.46–7.40 (m, 1H), 7.31 (d, *J* = 8.0, 1H), 7.27 (d, *J* = 8.0, 1H), 3.88 (td, *J* = 9.9, 3.9 Hz, 1H), 3.69 (s, 3H), 2.12 (dd, *J* = 9.5, 4.0 Hz, 2H), 1.79 (dt, *J* = 8.0, 3.7 Hz, 2H), 1.70–1.32 (m, 6H); ^13^C NMR (100 MHz, Chloroform-*d*) δ 159.7, 153.3, 133.5, 131.2, 128.2, 128.0, 123.7, 113.6, 42.1, 32.5, 29.2, 25.9; HRMS (ESI): *m*/*z* [M+H]^+^ calcd for C_1__5_H_19_N_2_OS: 275.1213; found: 275.1216. The compound spectra data is in agreement with the report [87].

1-methyl-3-((2-methyltetrahydrofuran-3-yl)thio)quinoxalin-2(1*H*)-one (**3al**):

White solid; mp 121–123 °C; 62.1 mg (isolated yield 75%); ^1^H NMR (400 MHz, Chloroform-*d*) δ 7.67 (d, *J* = 7.5 Hz, 1H), 7.41 (t, *J* = 7.8 Hz, 1H), 7.31–7.22 (m, 2H), 4.44–4.30 (m, 2H), 3.98 (td, *J* = 8.3, 5.4 Hz, 1H), 3.81–3.72 (m, 1H), 3.65 (s, 3H), 2.51 (td, *J* = 13.0, 7.5 Hz, 1H), 2.04 (dt, *J* = 13.0, 6.1 Hz, 1H), 1.21 (d, *J* = 5.9 Hz, 3H); ^13^C NMR (100 MHz, Chloroform-*d*) δ 159.6, 153.2, 133.4, 131.4, 128.4, 128.3, 123.9, 113.8, 76.6, 66.0, 45.8, 32.9, 29.3, 17.1; HRMS (ESI): *m*/*z* [M+H]^+^ calcd for C_1__4_H_17_N_2_O_2_S: 277.1005; found: 277.1012. The compound spectra data is in agreement with the report [37].

## 4. Conclusions

In summary, we have developed a visible-light induced sulfenylation of quinoxalin-2(1*H*)-ones employing g-C_3_N_4_ as a heterogeneous photocatalyst and ambient air as the sole oxidant. The process was chemo-, regioselective and provided direct access to a series of 3-sulfenylated quinoxalin-2(1*H*)-ones in good to excellent yields. Importantly, the photocatalyst can be easily recycled up to six times by simple filtration without the significant loss of its reaction efficiency. The environmentally friendly oxidant, recyclable photocatalyst and operation simplicity make this protocol highly attractive in organic synthesis and pharmaceutical chemistry.

## Figures and Tables

**Table 1:**
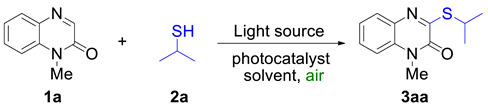
Optimization of reaction conditions ^a^.

**Entry**	**Light Source**	**Photocatalyst**	**Solvent**	**Yield of 3aa ^b^**
1	Blue (6 W, 450–455 nm)	g-C_3_N_4_ (10 mg)	THF	72%
2	Blue (6 W, 450–455 nm)	g-C_3_N_4_ (10 mg)	DCE	74%
3	Blue (6 W, 450–455 nm)	g-C_3_N_4_ (10 mg)	CH_3_CN	8%
4	Blue (6 W, 450–455 nm)	g-C_3_N_4_ (10 mg)	EtOAc	82%
5	Blue (6 W, 450–455 nm)	g-C_3_N_4_ (10 mg)	EtOH	62%
6	Blue (6 W, 450–455 nm)	g-C_3_N_4_ (10 mg)	DMSO	41%
7	Blue (6 W, 450–455 nm)	g-C_3_N_4_ (10 mg)	H_2_O	0%
**8**	**Sunlight**	**g-C_3_N_4_ (10 mg)**	**EtOAc**	**87%**
9	Green (6 W, 520–525 nm)	g-C_3_N_4_ (10 mg)	EtOAc	0%
10	White (6 W)	g-C_3_N_4_ (10 mg)	EtOAc	67%
11	Purple (6 W, 370–375 nm)	g-C_3_N_4_ (10 mg)	EtOAc	74%
12	Sunlight	g-C_3_N_4_ (15 mg)	EtOAc	87%
13	Sunlight	g-C_3_N_4_ (5 mg)	EtOAc	71%
14 ^c^	Sunlight	g-C_3_N_4_ (10 mg)	EtOAc	0%
15	None	g-C_3_N_4_ (10 mg)	EtOAc	0%
16	Sunlight	None	EtOAc	0%

^a^ Conditions: **1a** (0.3 mmol), **2a** (0.9 mmol), photocatalyst, solvent (1.5 mL), air, rt, 12 h. ^b^ Isolated yields. ^c^ under N_2_ atmosphere.

## Data Availability

The data presented in this study are available on request from the corresponding author.

## Supplementary Materials

The following supporting information can be downloaded at: https://www.mdpi.com/article/10.3390/molecules27155044/s1. Copies of the ^1^H NMR and ^13^C NMR spectra for compounds **3aa**–**3ta** and **3ab**–**3al** can be found in Supplementary Materials.

## Sample Availability

Not available.
