# Supplementary material for: Sunlight Induced and Recyclable g-C3N4 Catalyzed C-H Sulfenylation of Quinoxalin-2(1H)-Ones
Source: Molecules. 2022 Aug 8;27(15):5044. doi: 10.3390/molecules27155044 (PMC9370749; doi:10.3390/molecules27155044)
Supplement: Supplementary file 1 [file molecules-27-05044-s001.zip › molecules-1843013-supplementary.pdf]

Sunlight induced and Recyclable g-C_3N_4 Catalyzed C-H Sulfenylation of Quinoxalin-2(1*H*)-ones

Sha Peng, Jiao Liu, Li-Hua Yang , and Long-Yong Xie *

College of Chemistry and Bioengineering, Hunan University of Science and Engineering, Hunan, 425100, China

E-mail: xielongyong@huse.edu.cn

^{1}H and ^{13}C NMR spectra of products

3-(isopropylthio)-1-methylquinoxalin-2(1*H*)-one (3aa)

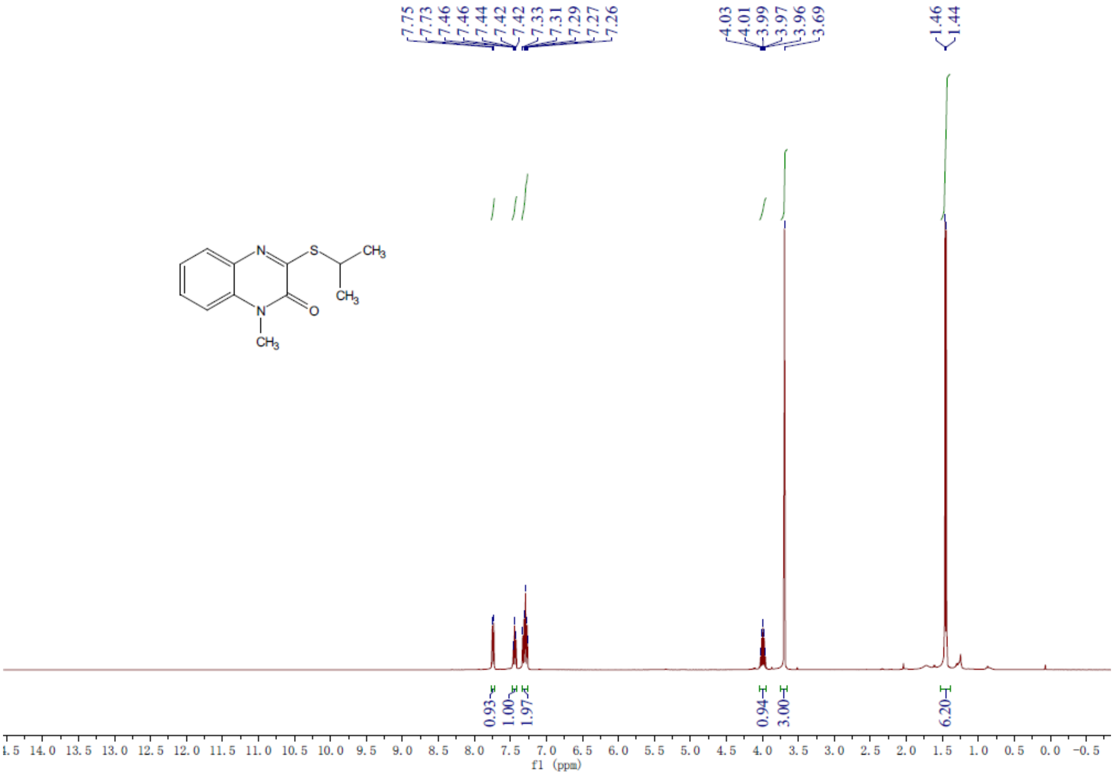

^{1}H spectra of **3aa**

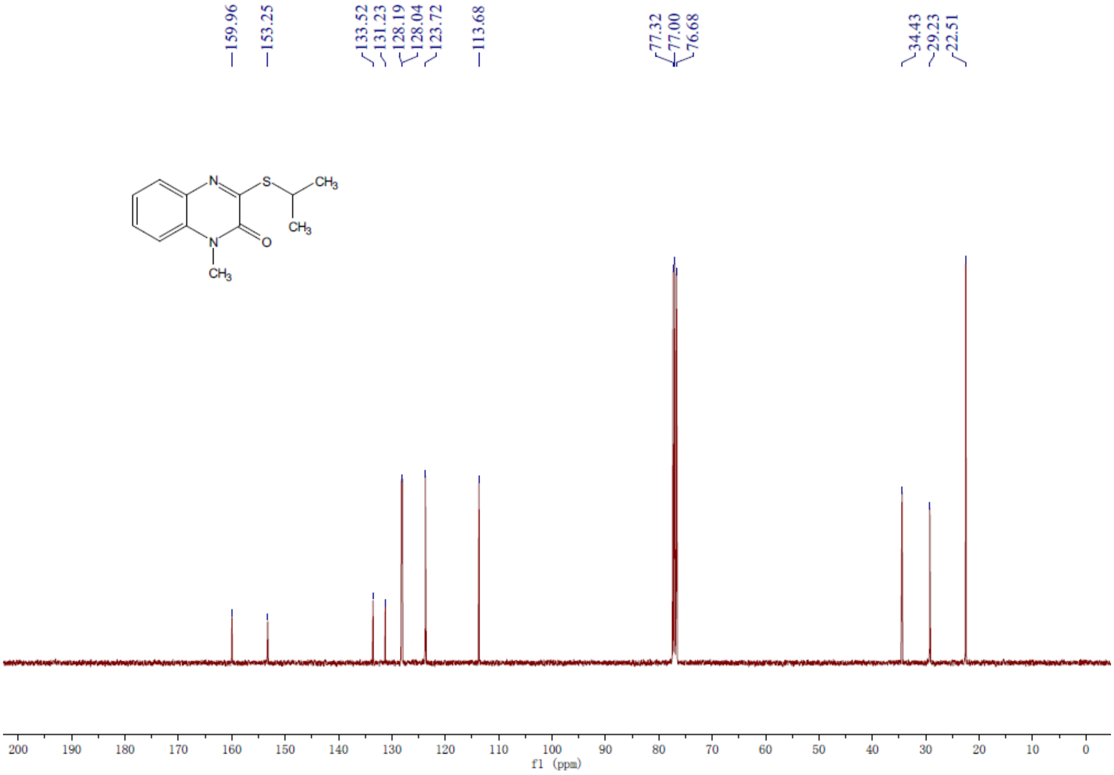

^{13}C spectra of **3aa**

1-ethyl-3-(isopropylthio)quinoxalin-2(1*H*)-one (3ba)

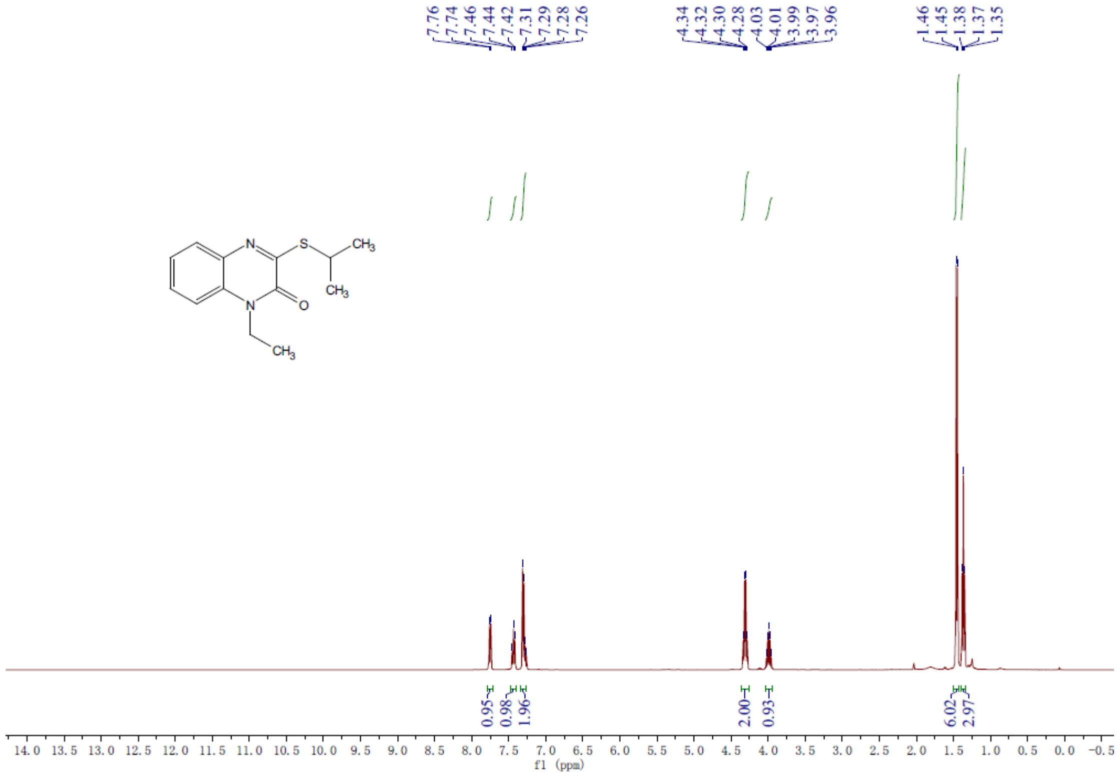

^{1}H spectra of **3ba**

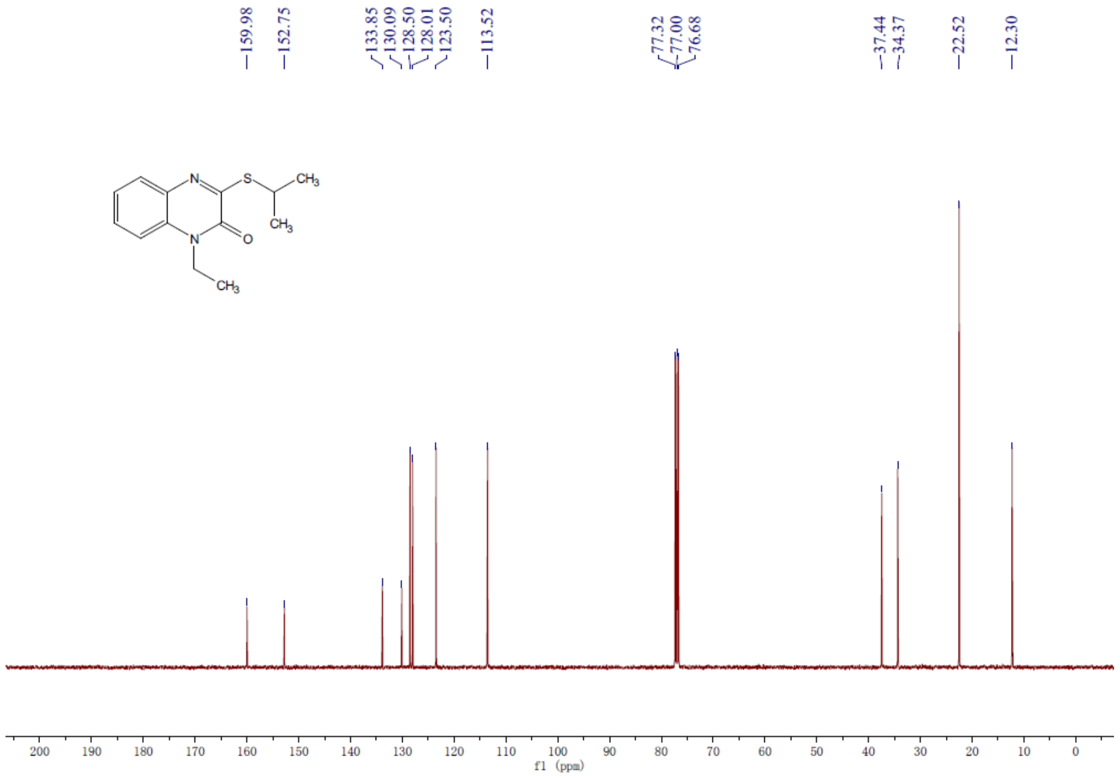

^{13}C spectra of **3ba**

3-(isopropylthio)-1-pentylquinoxalin-2(1*H*)-one (3ca)

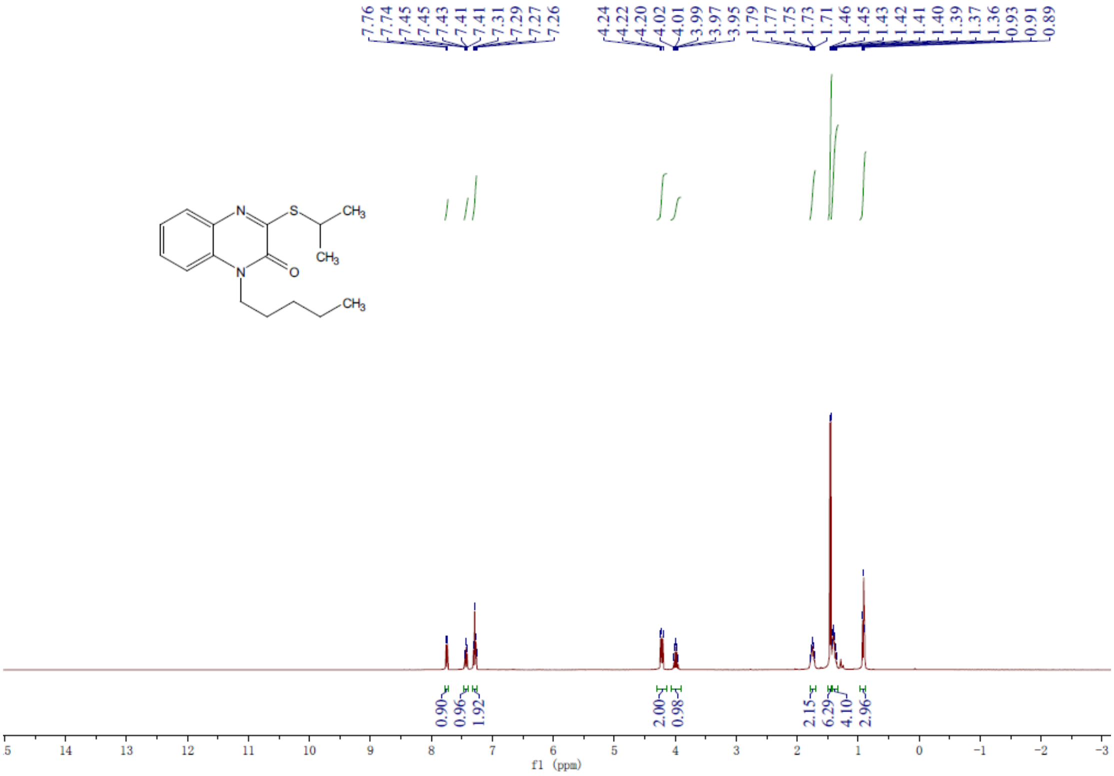

^{1}H spectra of **3ca**

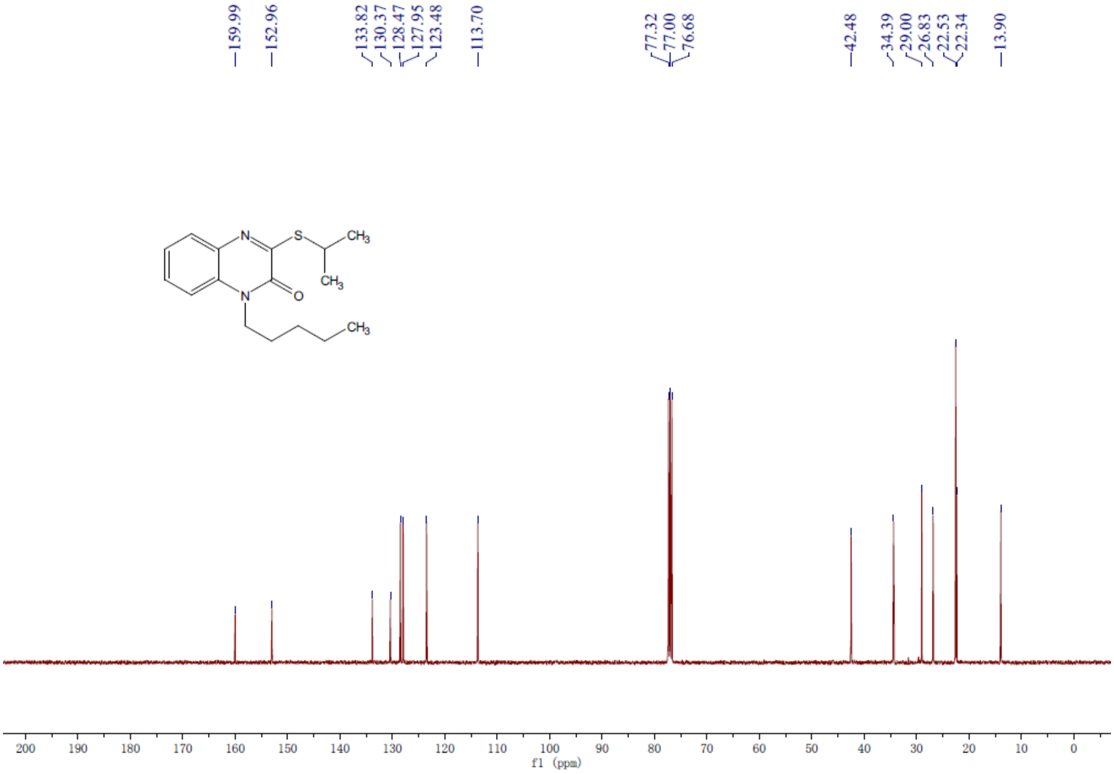

^{13}C spectra of **3ca**

benzyl-3-(isopropylthio)quinoxalin-2(1*H*)-one (3da)

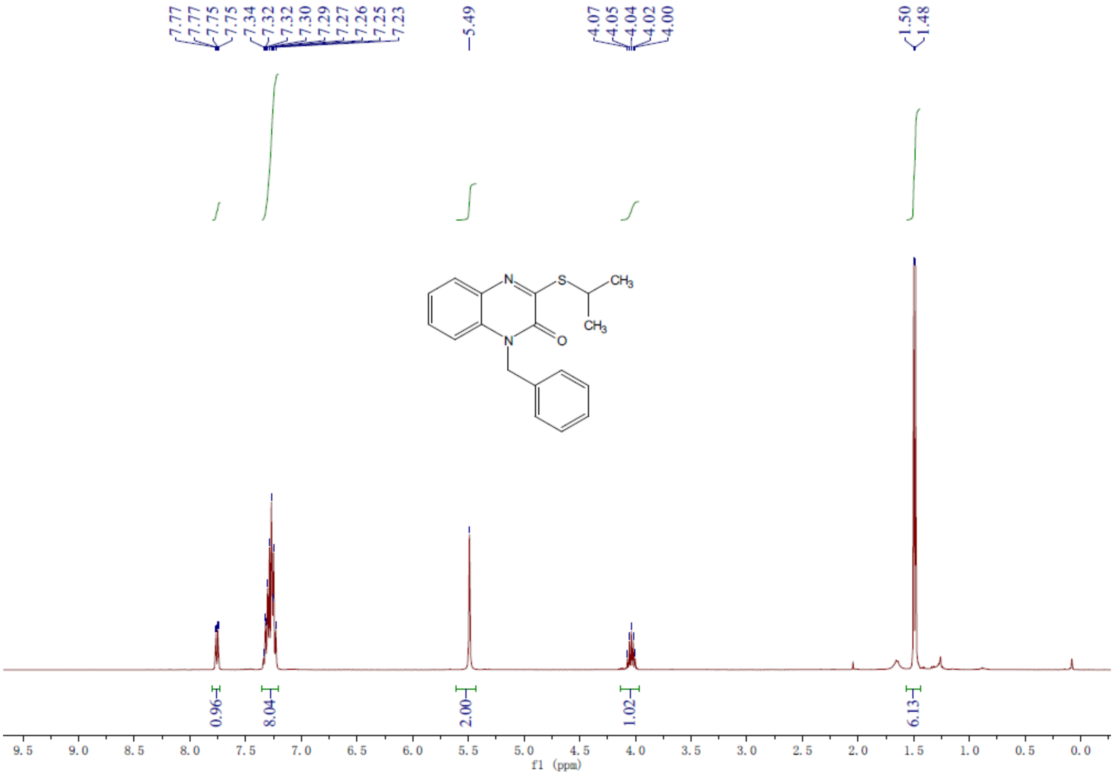

^{1}H spectra of **3da**

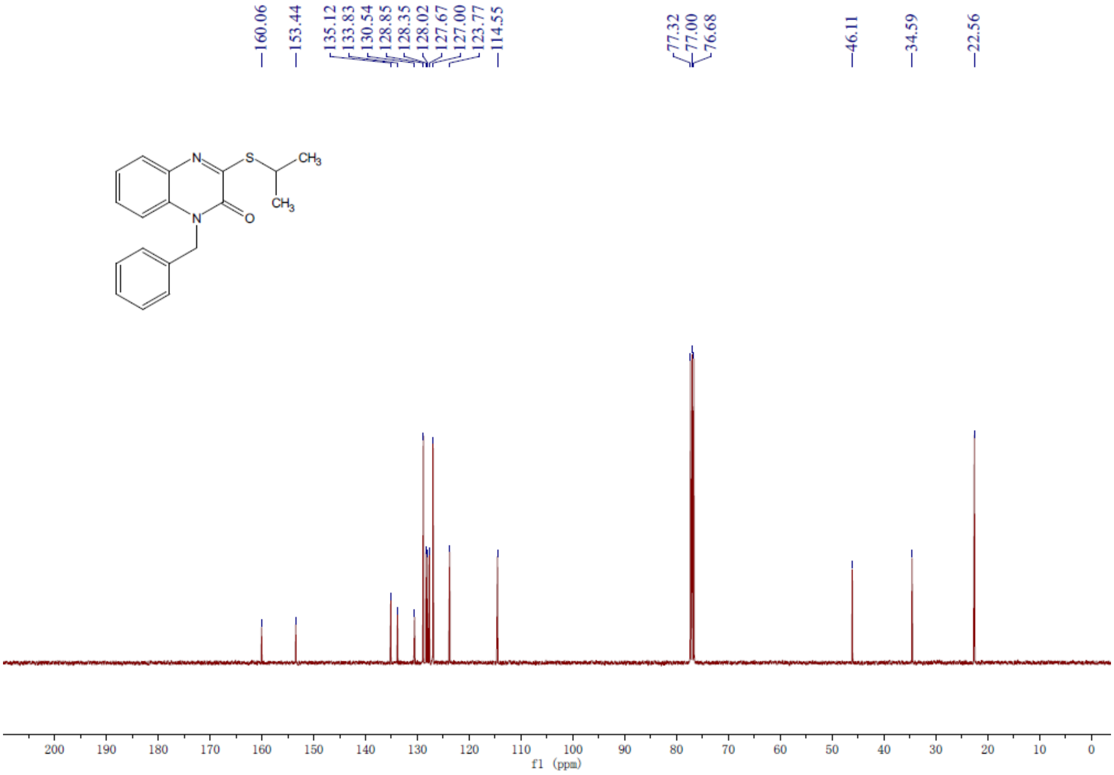

^{13}C spectra of **3da**

allyl-3-(isopropylthio)quinoxalin-2(1*H*)-one (3ea)

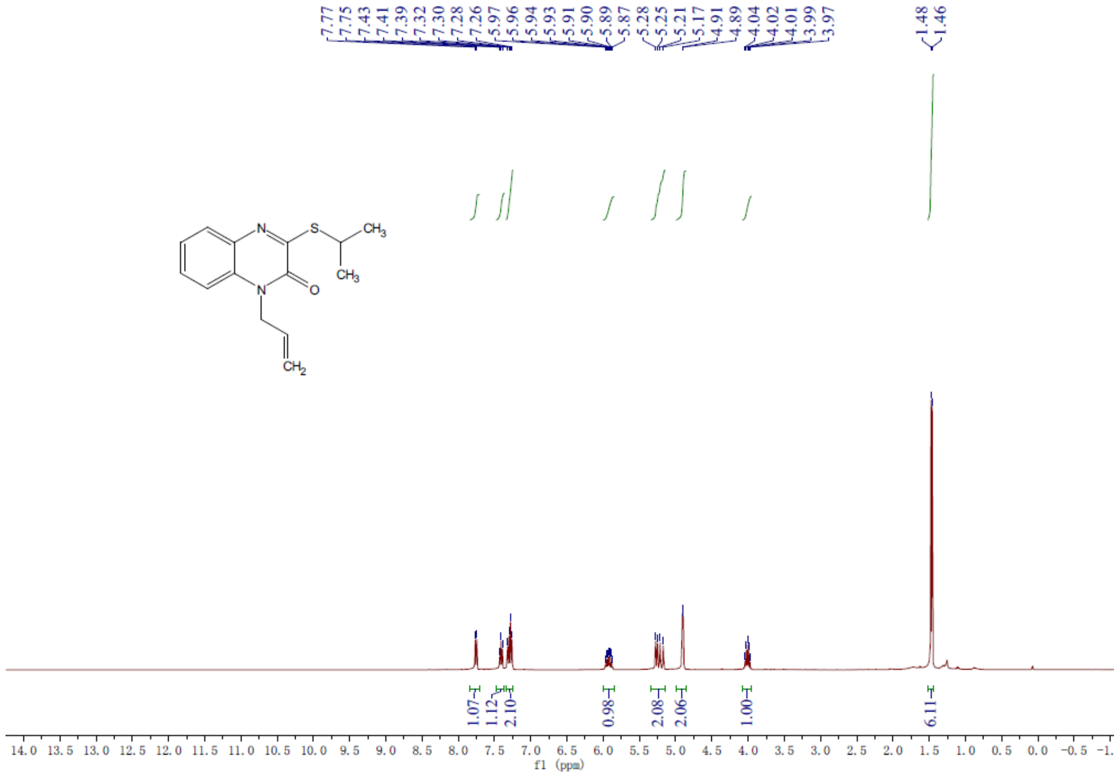

^{1}H spectra of **3ea**

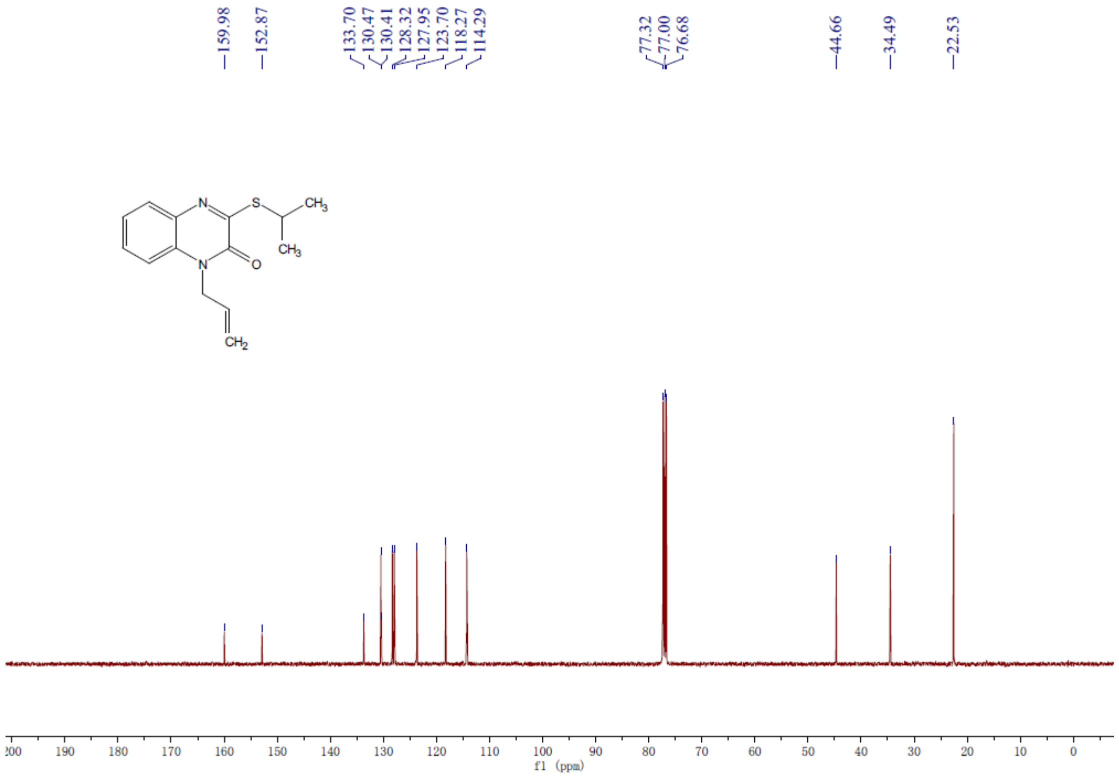

^{13}C spectra of **3ea**

3-(isopropylthio)-1-(prop-2-yn-1-yl)quinolin-2(1*H*)-one (3fa)

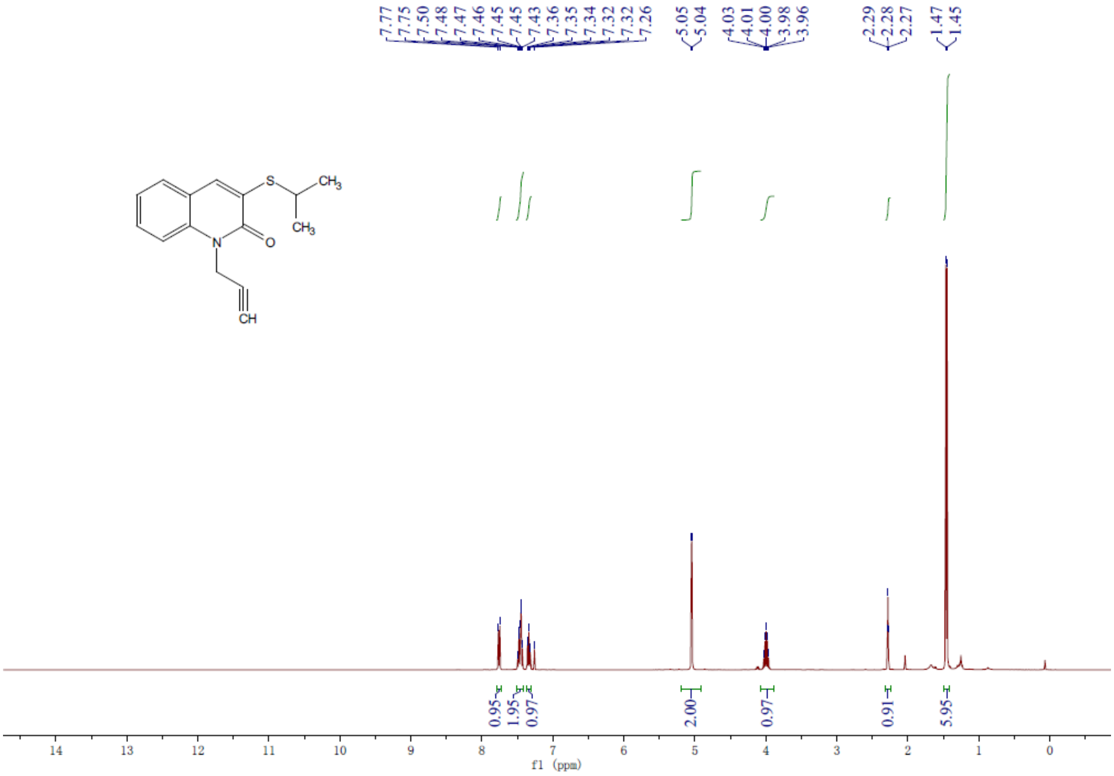

^{1}H spectra of **3fa**

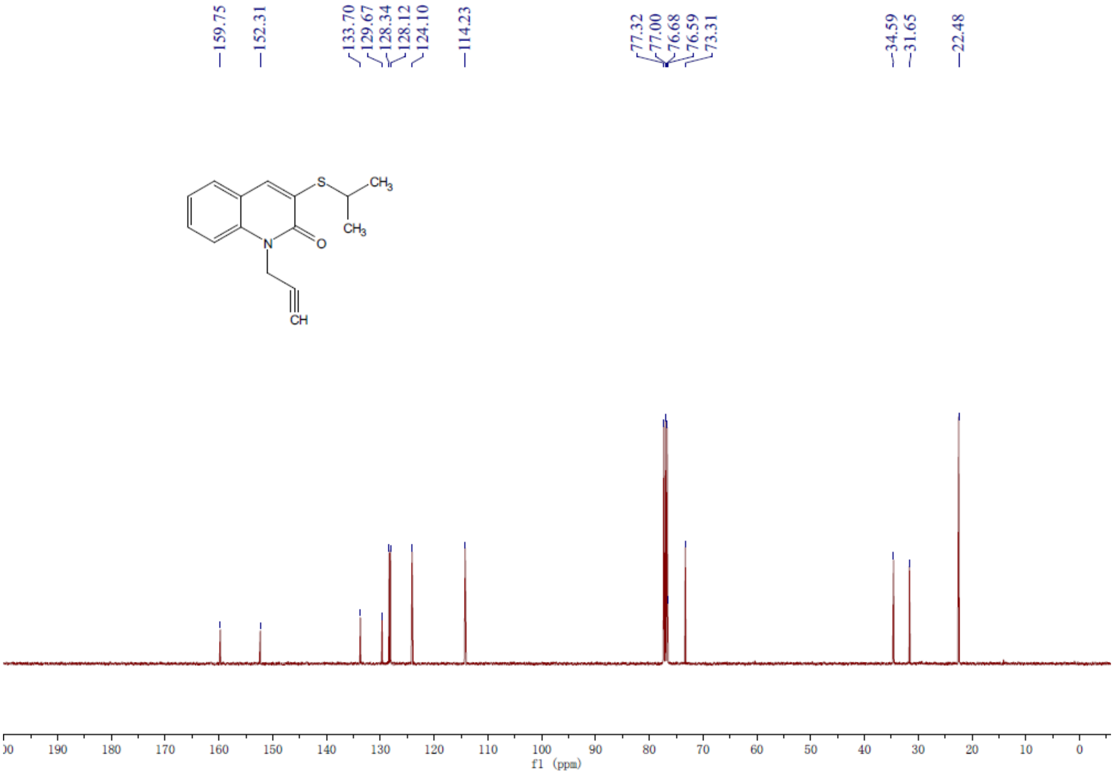

^{13}C spectra of **3fa**

ethyl 2-(3-(isopropylthio)-2-oxoquinoxalin-1(2*H*)-yl)acetate (3ga)

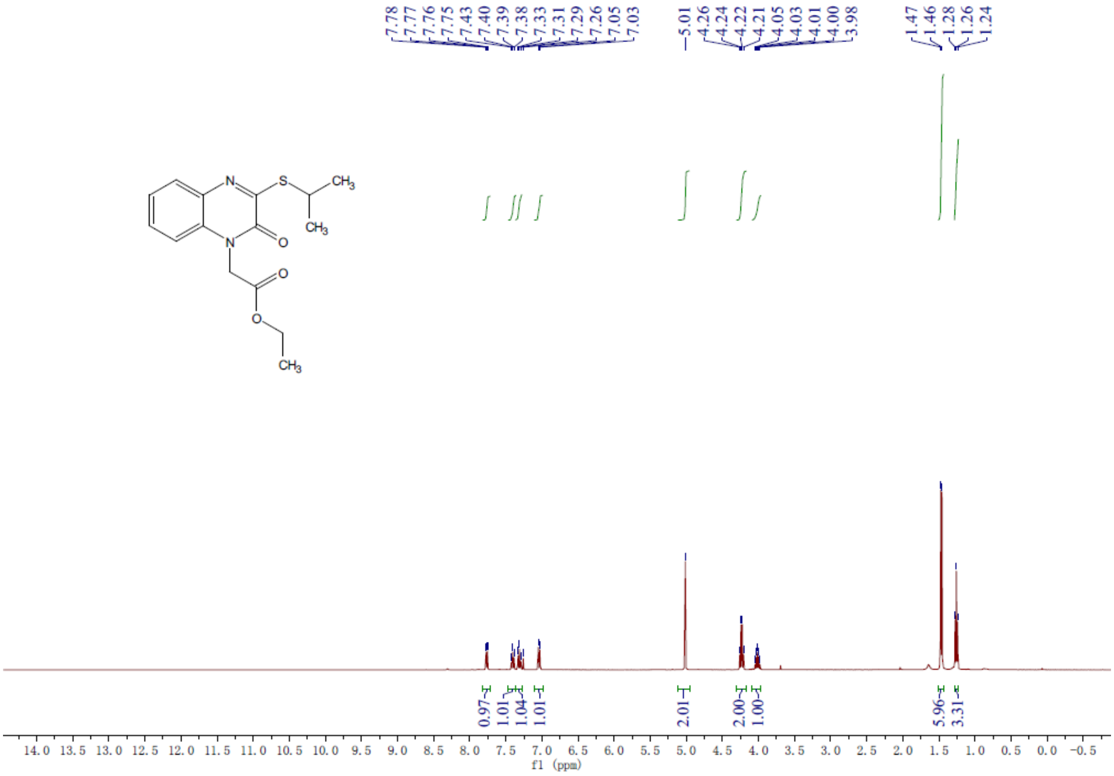

^{1}H spectra of **3ga**

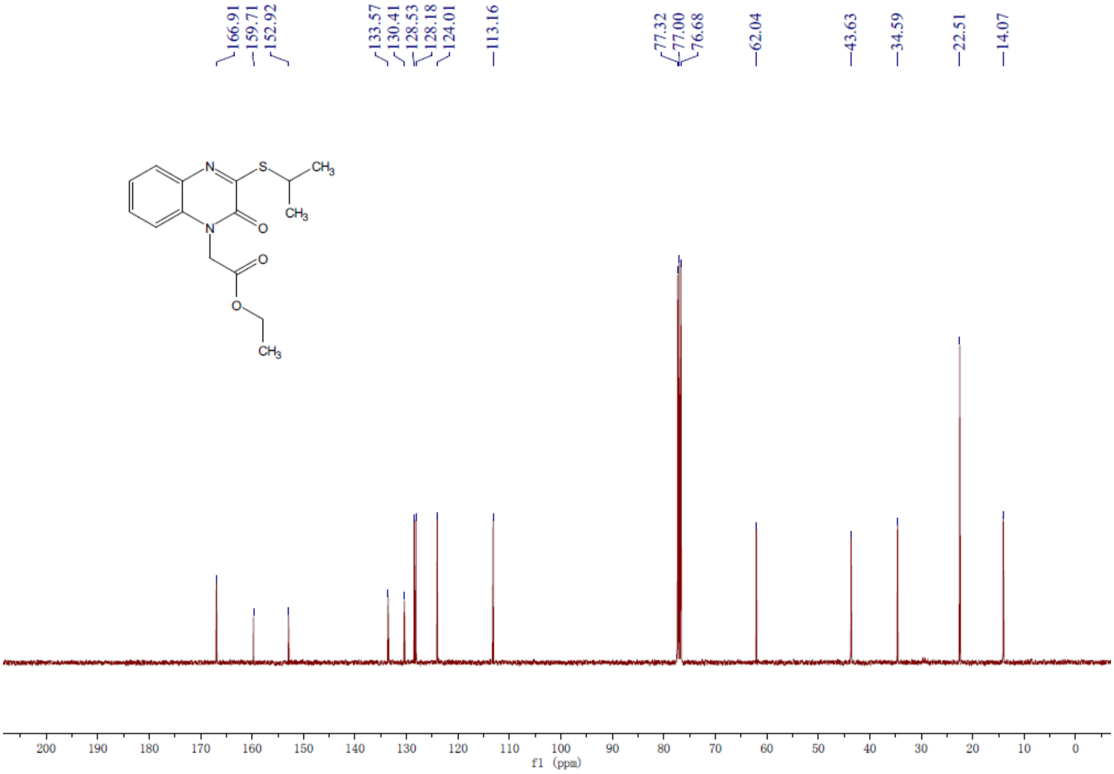

^{13}C spectra of **3ga**

3-(isopropylthio)-1-(2-oxo-2-phenylethyl)quinoxalin-2(1*H*)-one (3ha)

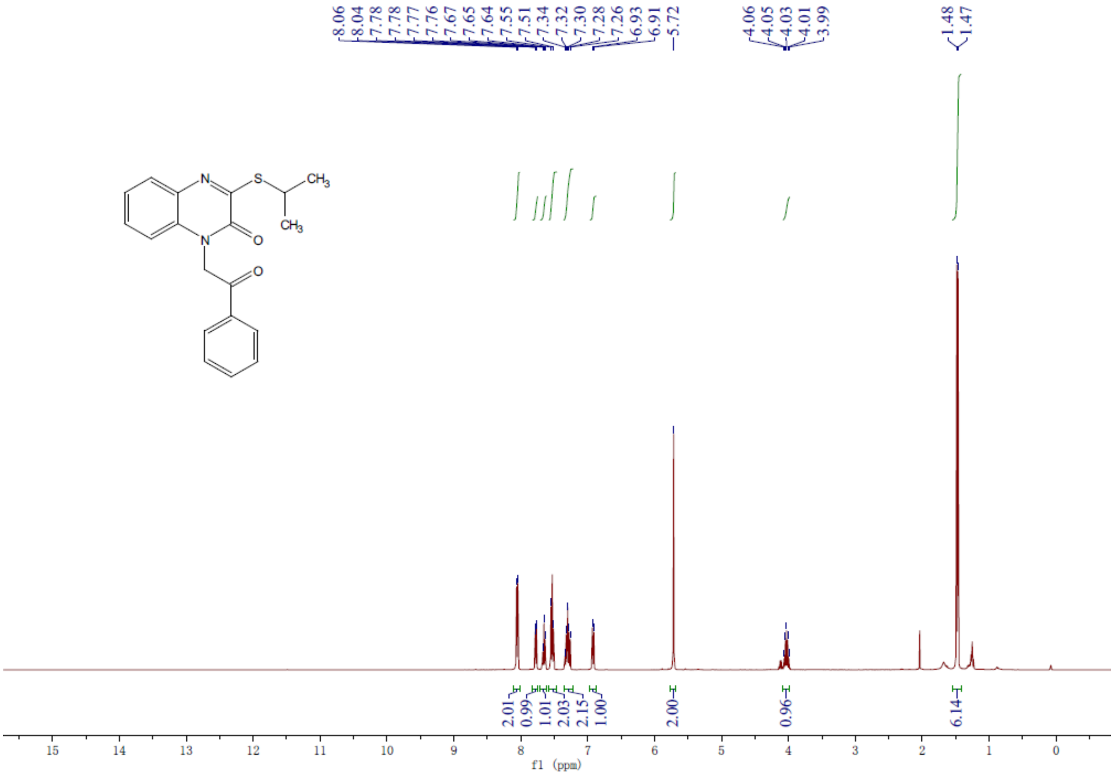

^{1}H spectra of **3ha**

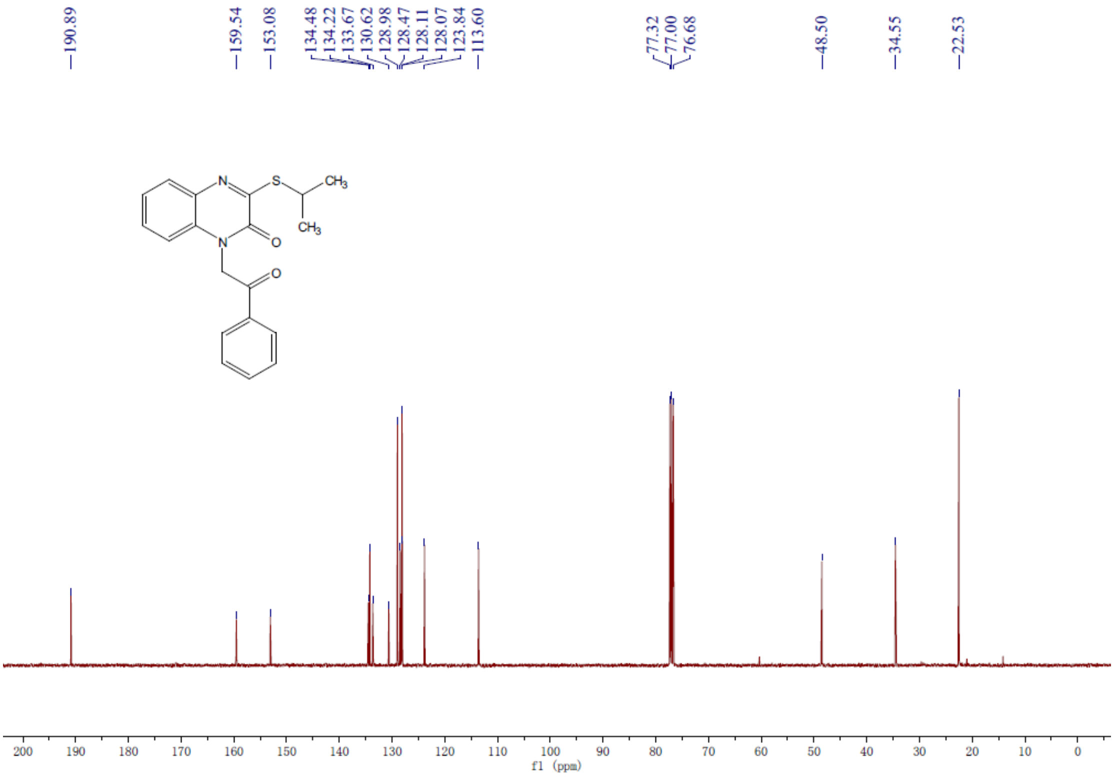

^{13}C spectra of **3ha**

3-(isopropylthio)-1-phenylquinoxalin-2(1*H*)-one (3ia)

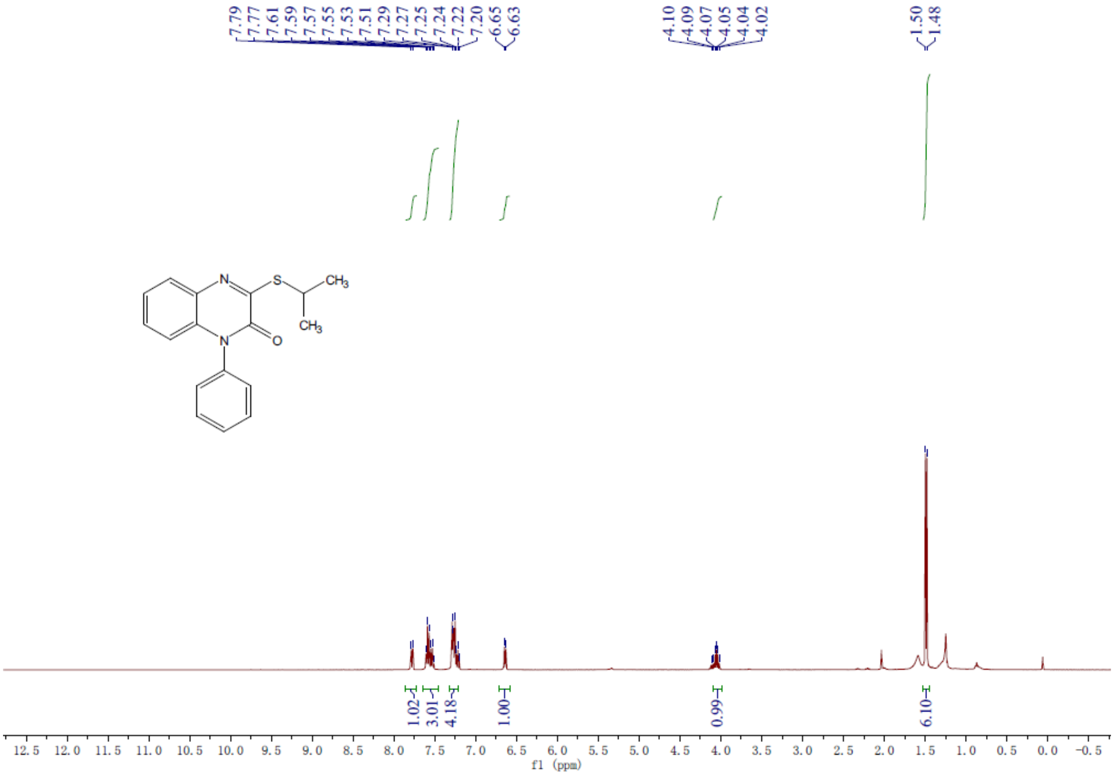

^{1}H spectra of **3ia**

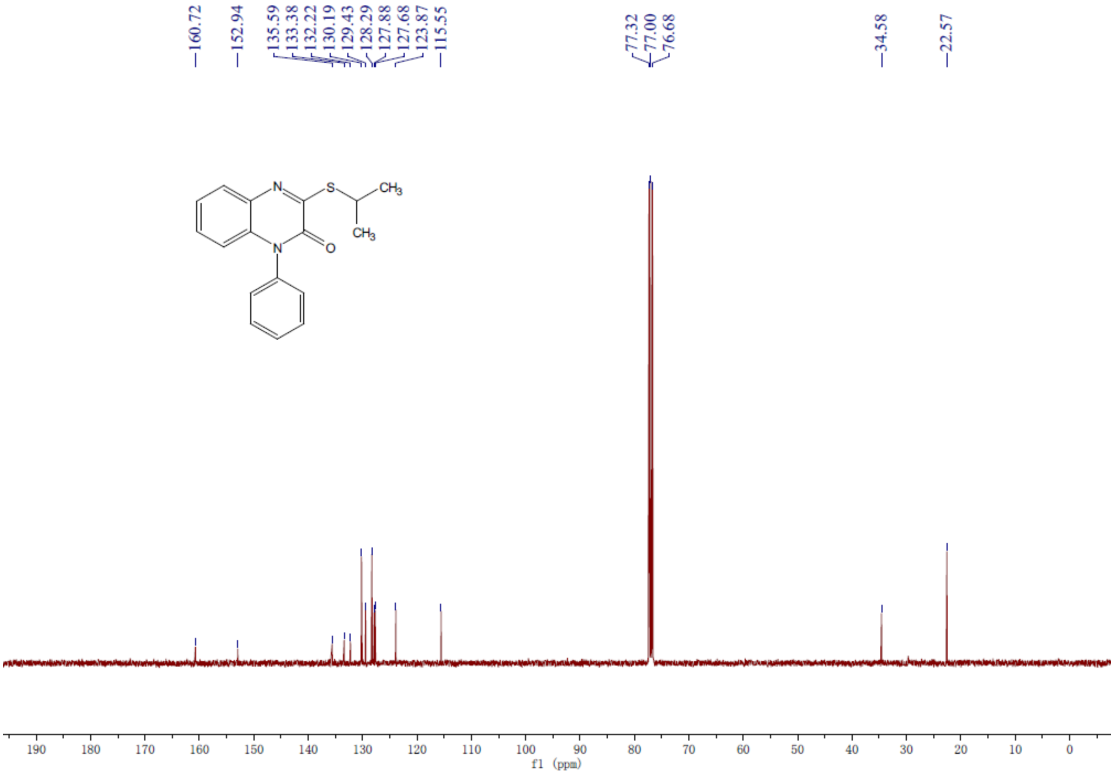

^{13}C spectra of **3ia**

5-chloro-3-(isopropylthio)-1-methylquinoxalin-2(1*H*)-one (3ja)

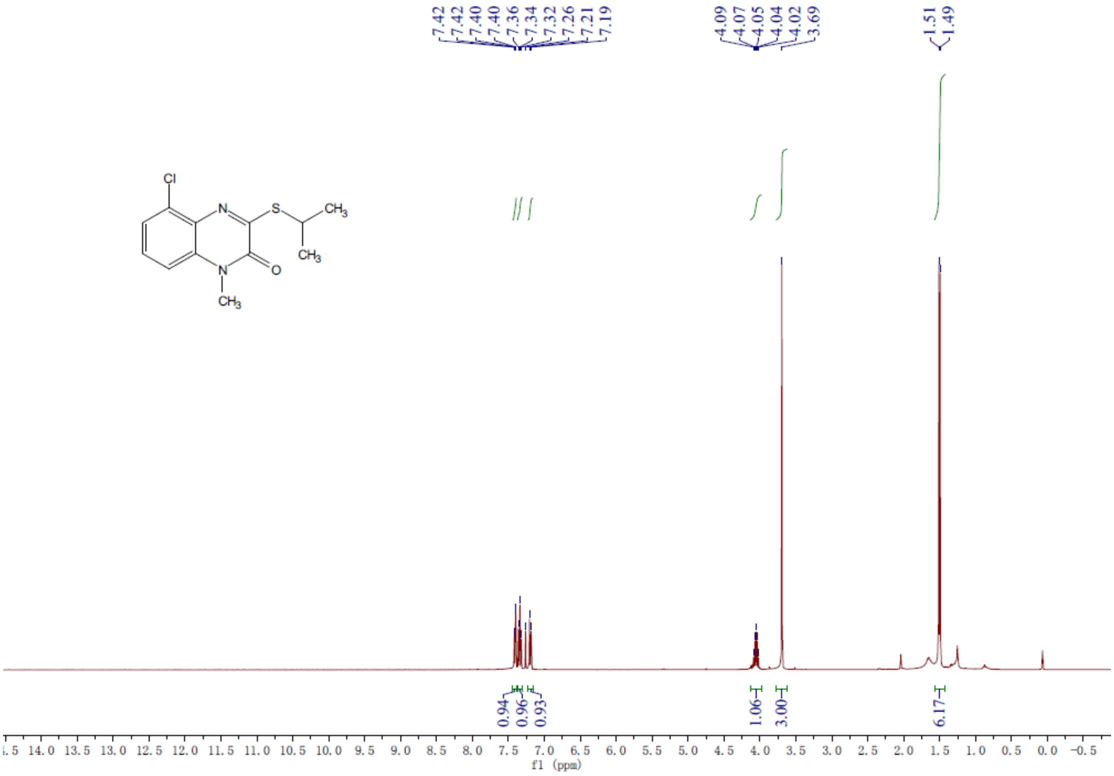

^{1}H spectra of **3ja**

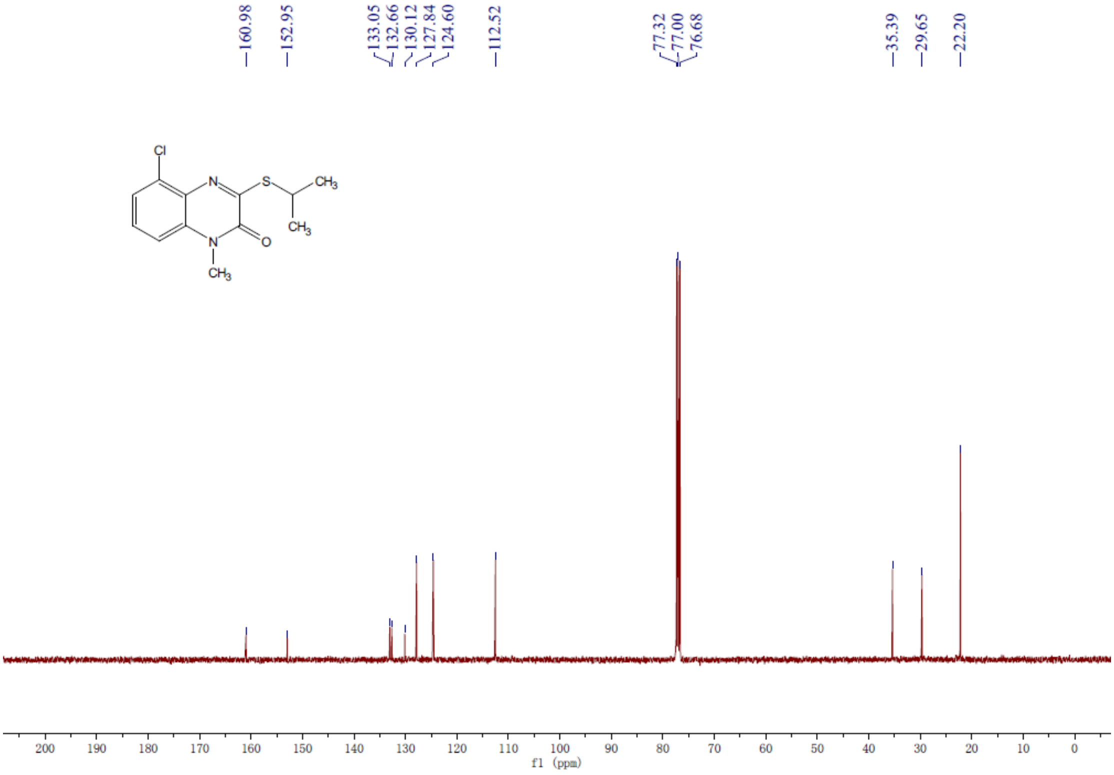

^{13}C spectra of **3ja**

6-fluoro-3-(isopropylthio)-1-methylquinoxalin-2(1*H*)-one (3ka)

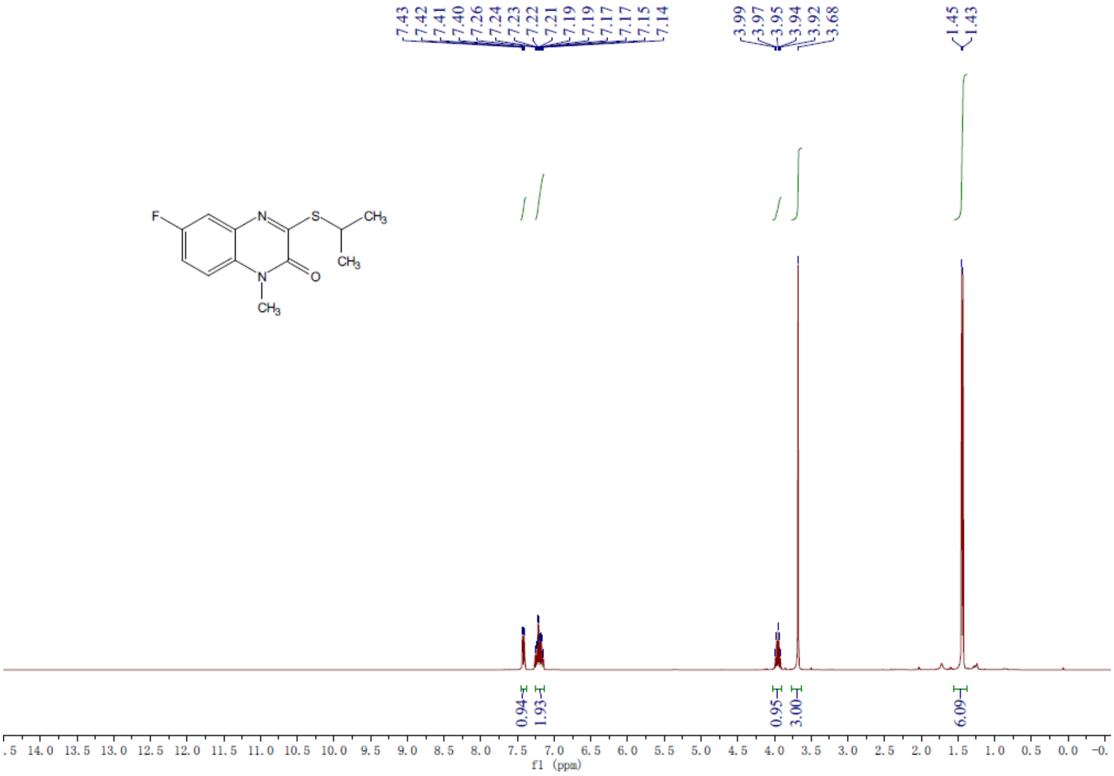

^{1}H spectra of **3ka**

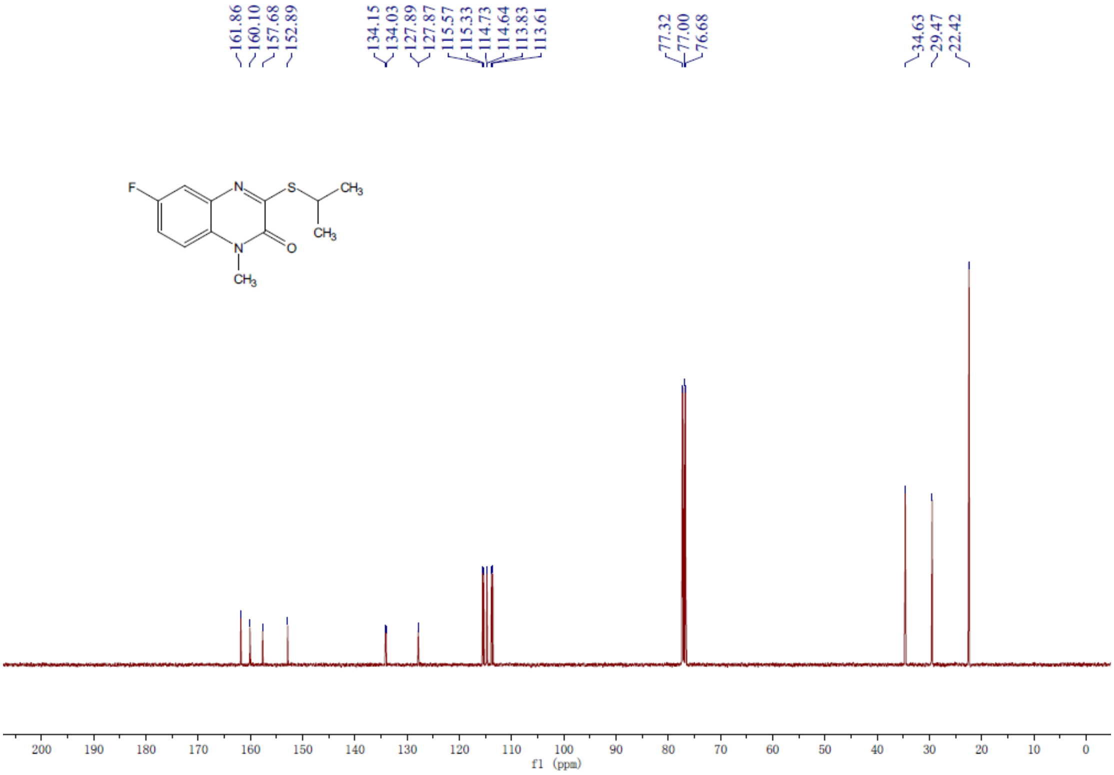

^{13}C spectra of **3ka**

6-chloro-3-(isopropylthio)-1-methylquinolin-2(1*H*)-one (3la)

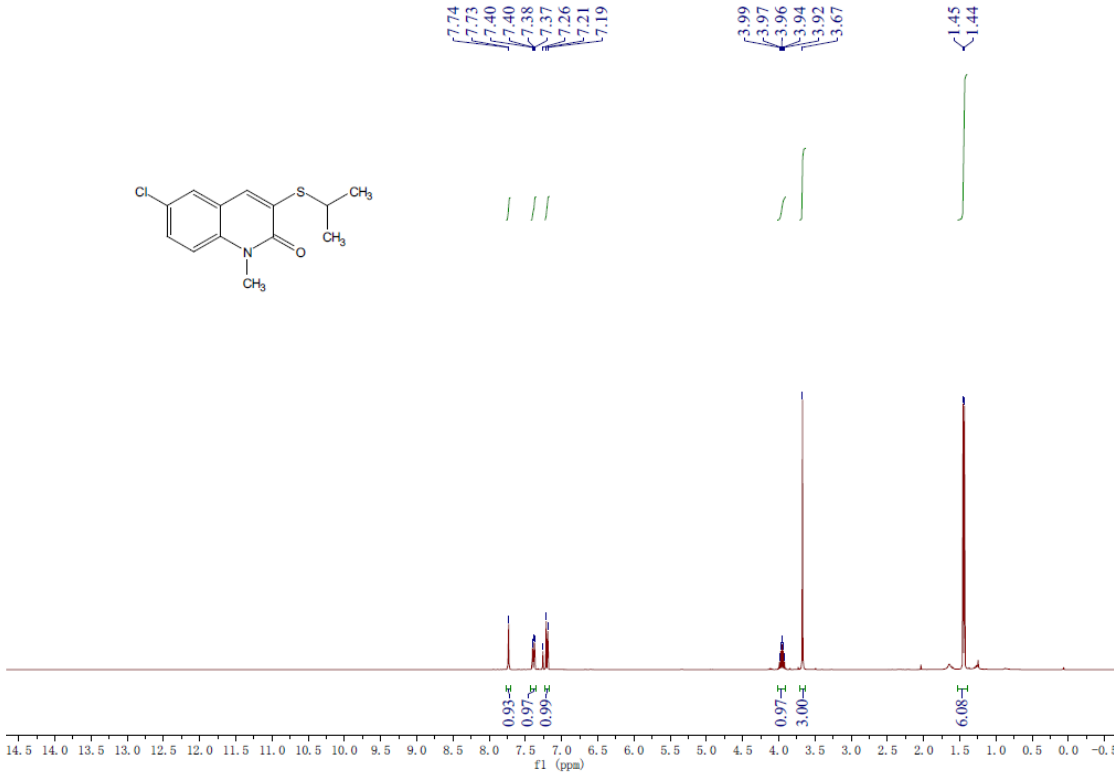

^{1}H spectra of **3la**

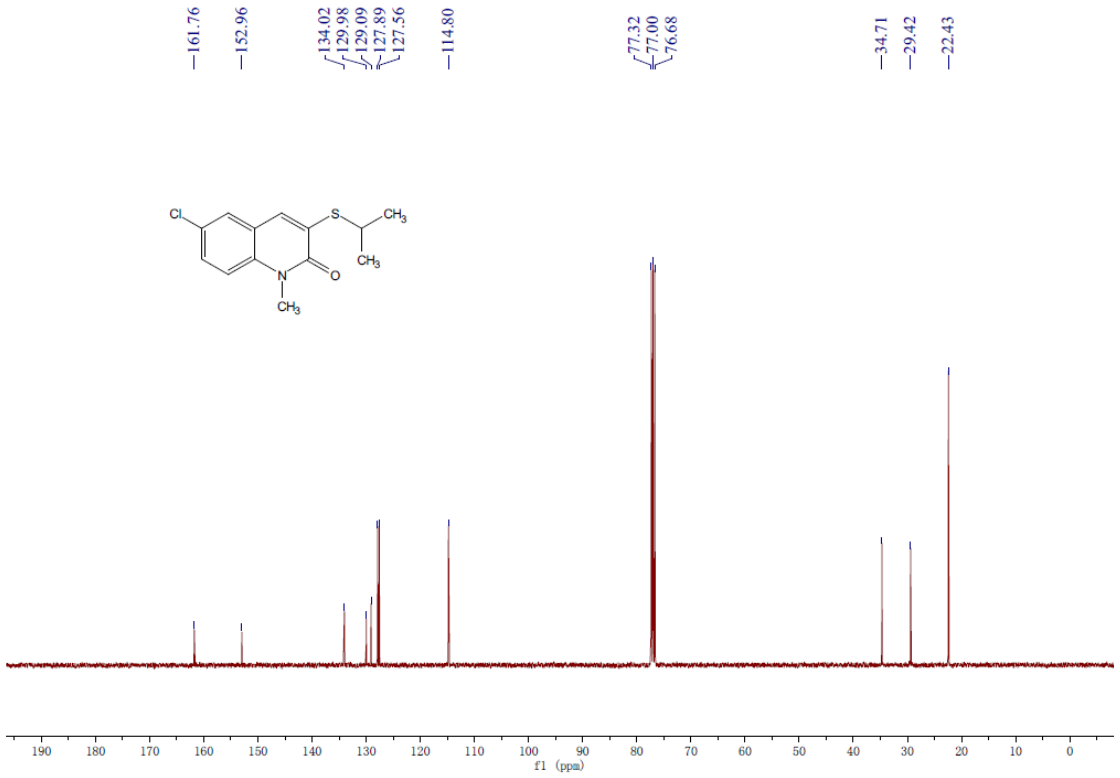

^{13}C spectra of **3la**

6-bromo-3-(isopropylthio)-1-methylquinolin-2(1*H*)-one (3ma)

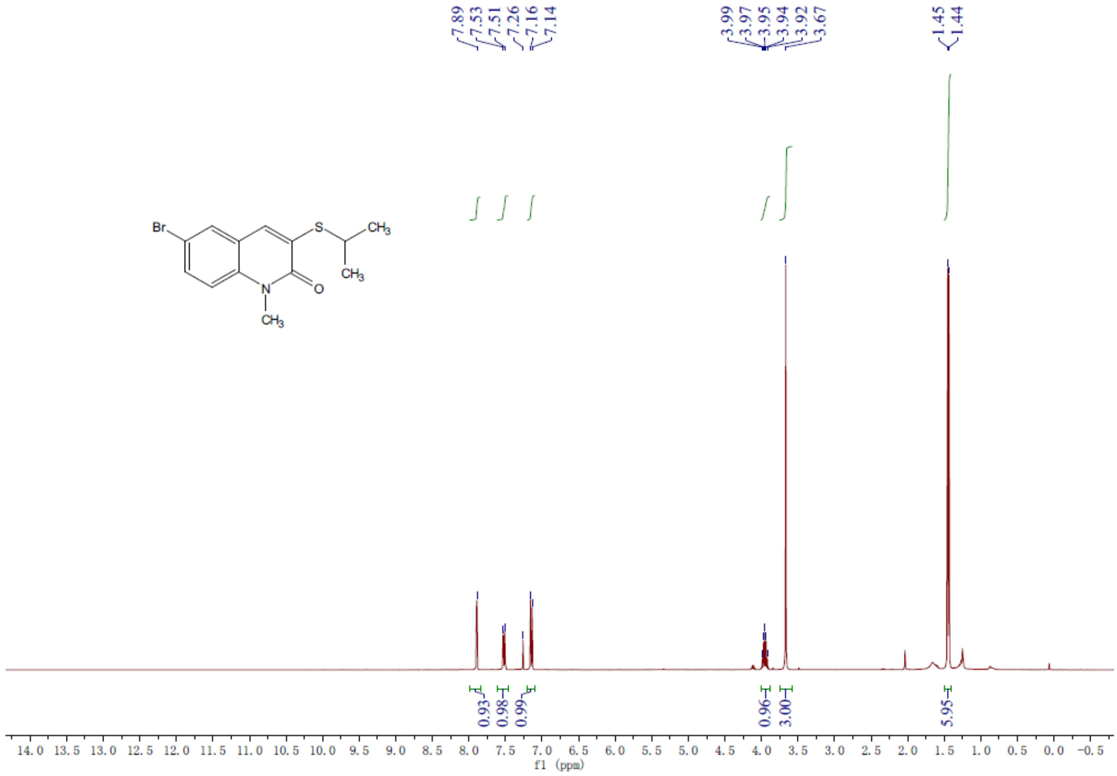

^{1}H spectra of **3ma**

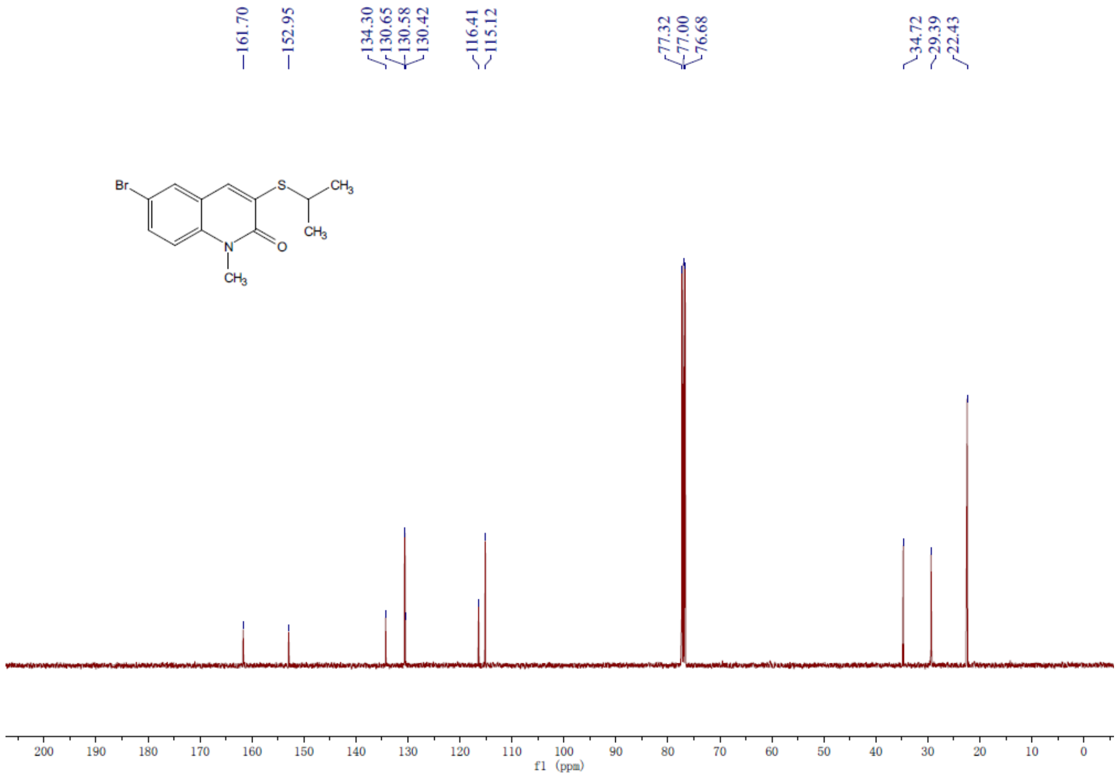

^{13}C spectra of **3ma**

3-(isopropylthio)-1-methyl-6-(trifluoromethyl)quinolin-2(1*H*)-one (3na)

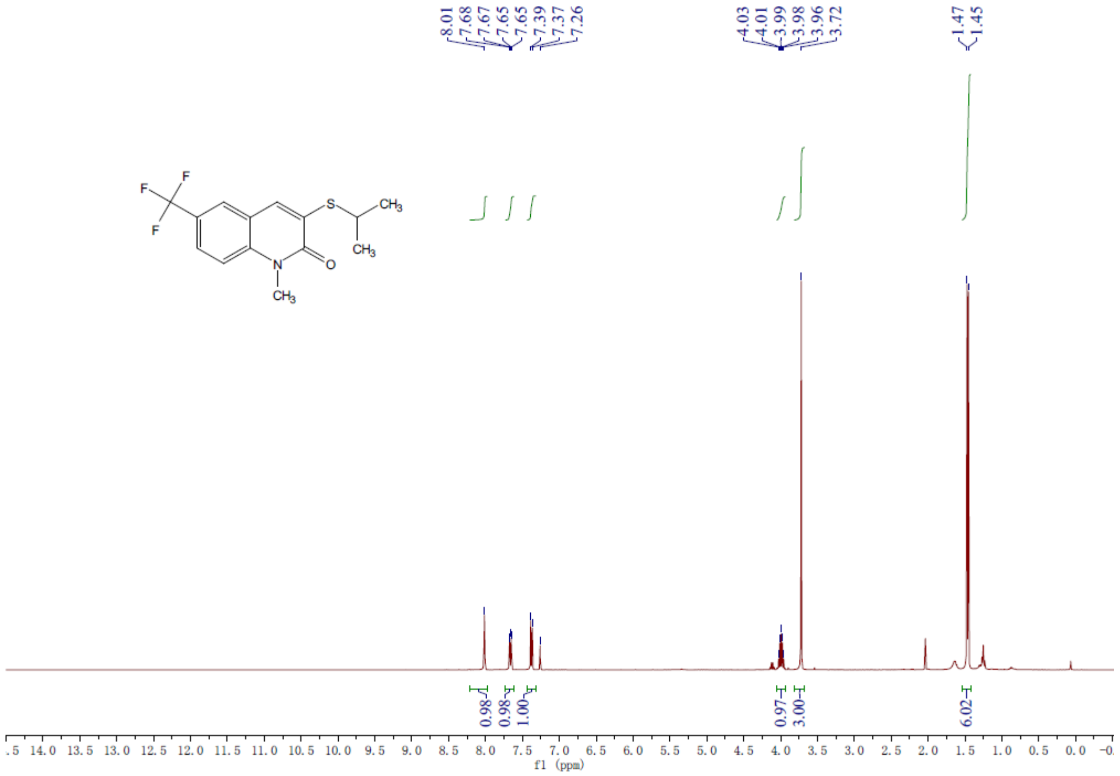

^{1}H spectra of **3na**

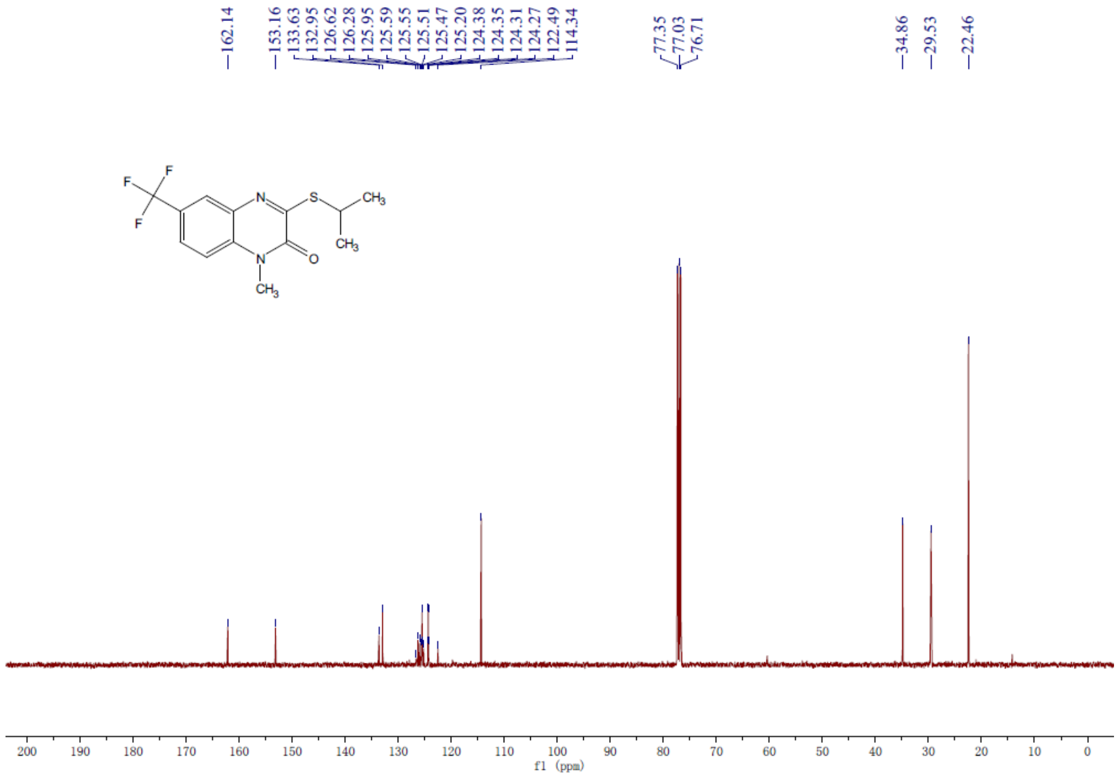

^{13}C spectra of **3na**

6-benzoyl-3-(isopropylthio)-1-methylquinoxalin-2(1*H*)-one (3oa)

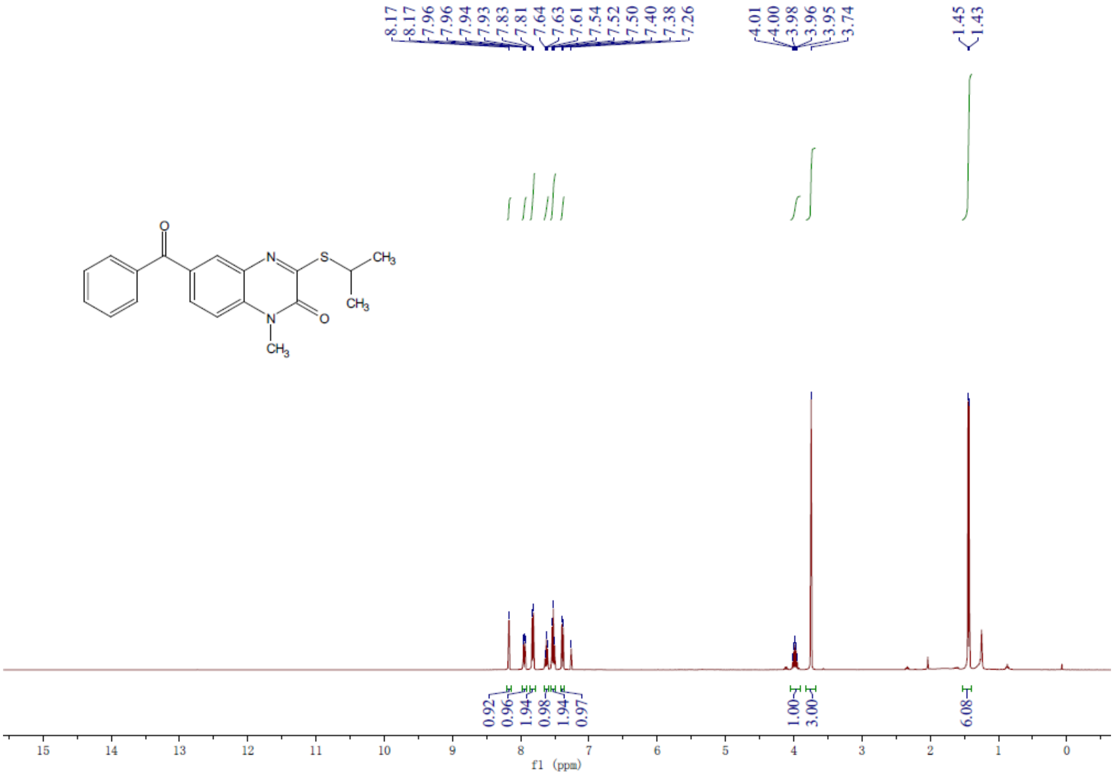

^{1}H spectra of **3oa**

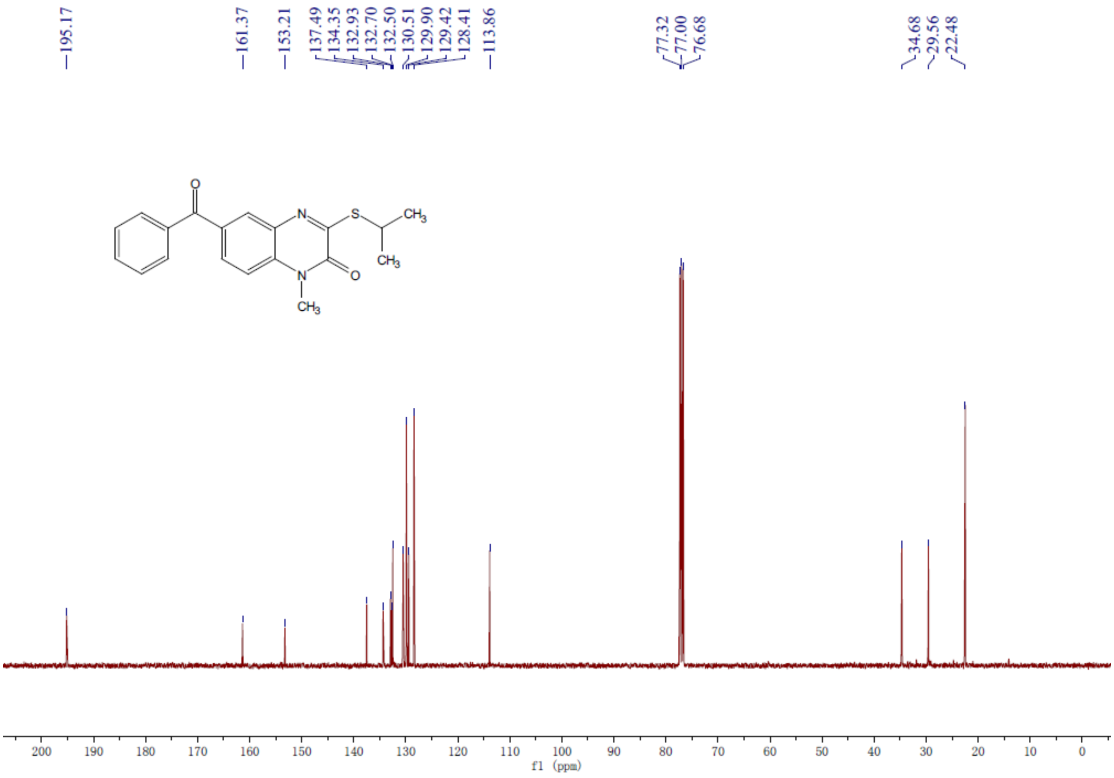

^{13}C spectra of **3oa**

7-fluoro-3-(isopropylthio)-1-methylquinolin-2(1*H*)-one (3pa)

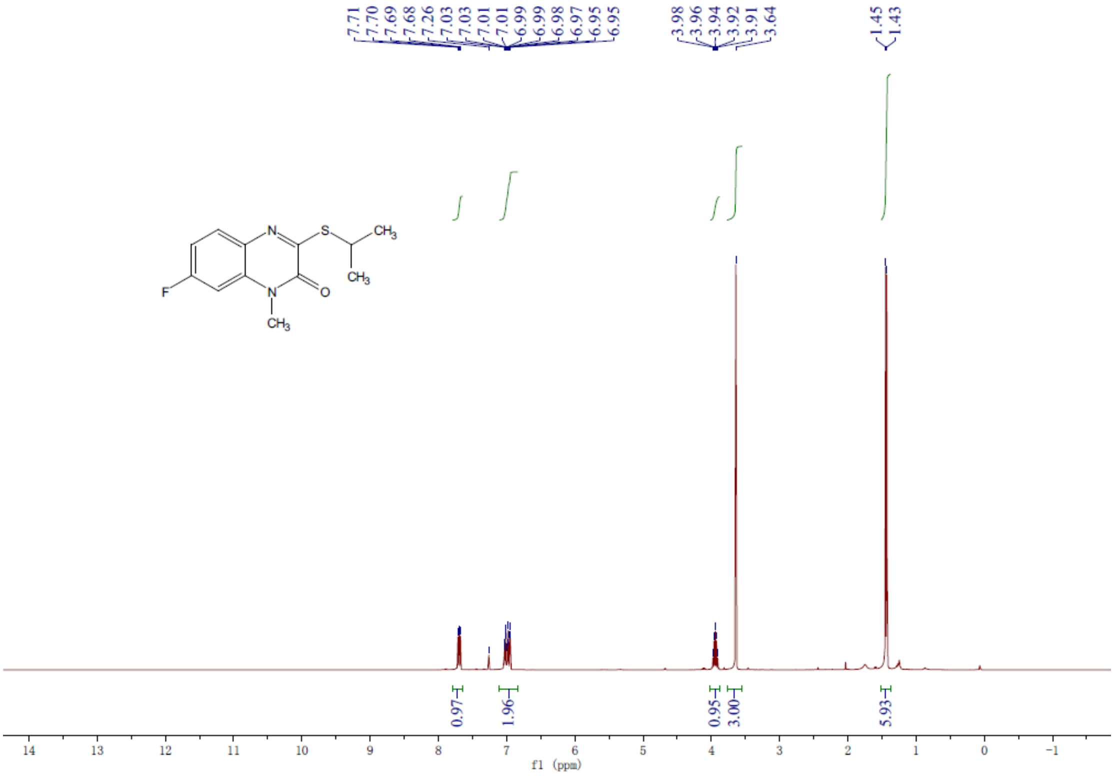

^{1}H spectra of **3pa**

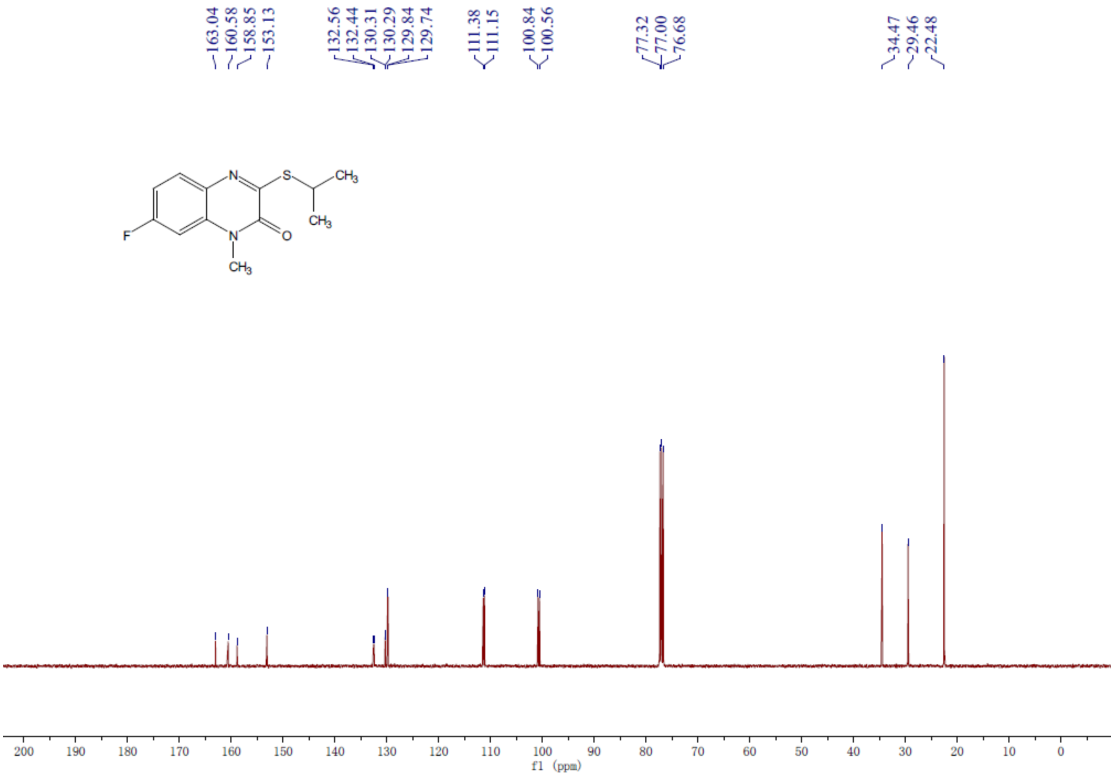

^{13}C spectra of **3pa**

3-(isopropylthio)-1-methyl-7-(trifluoromethyl)quinolin-2(1*H*)-one (3qa)

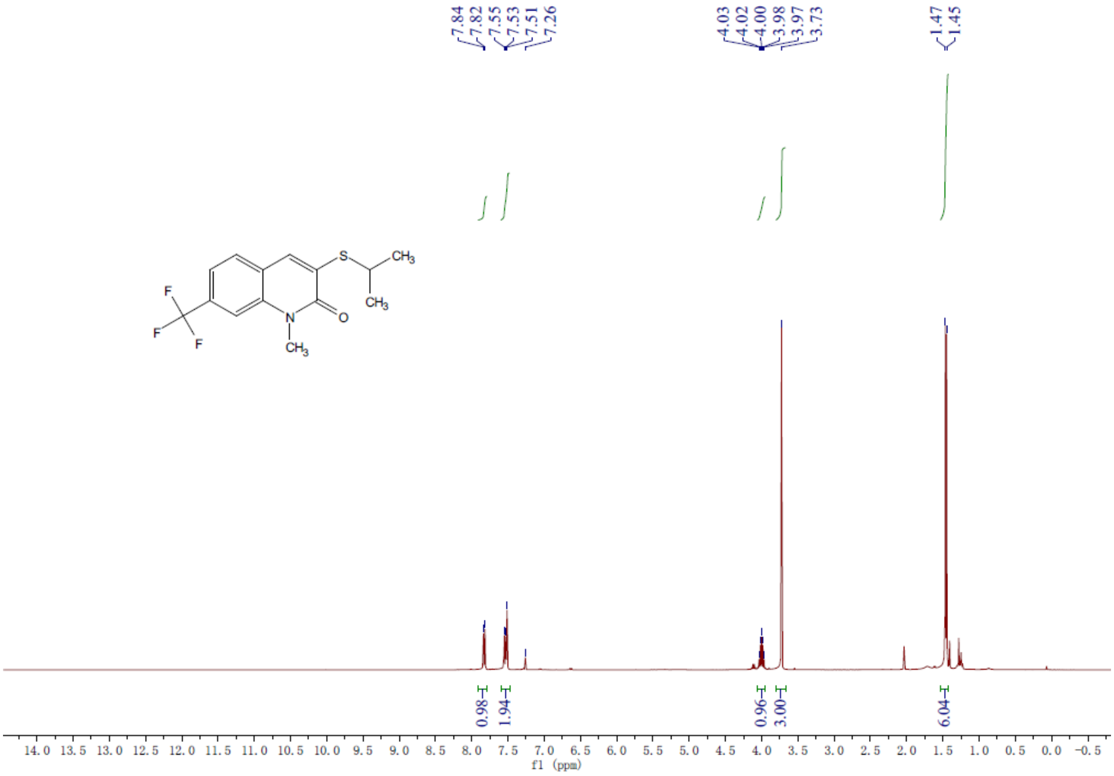

^{1}H spectra of **3qa**

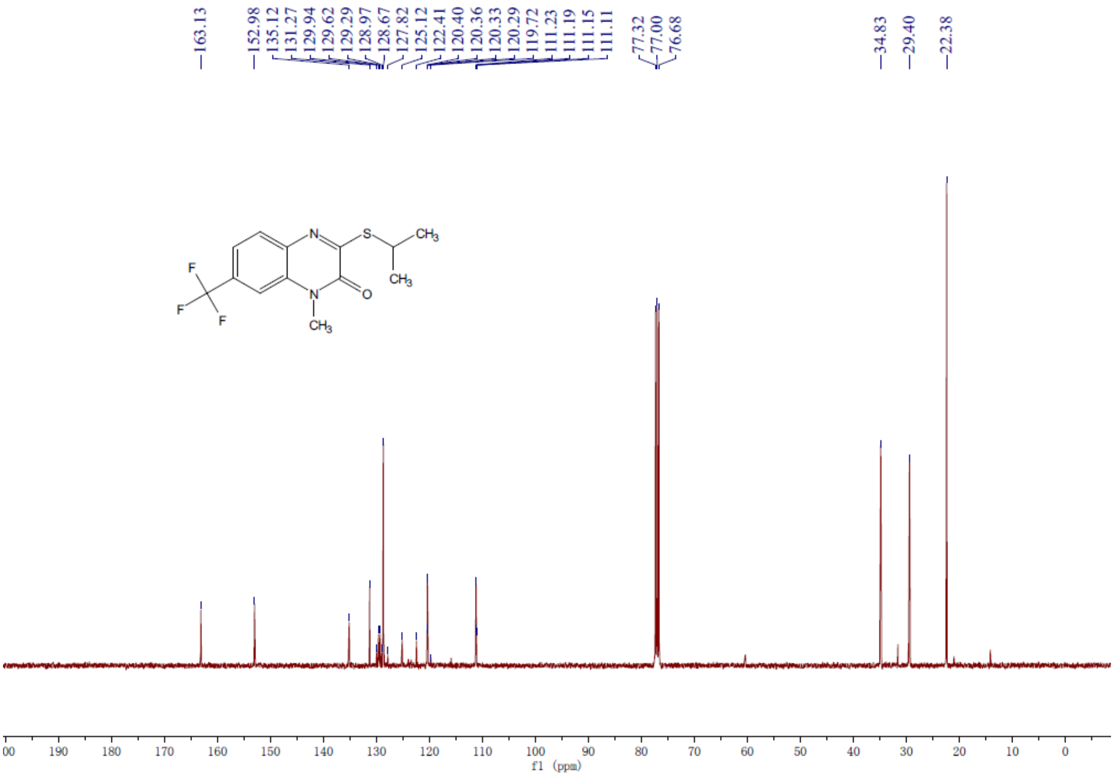

^{13}C spectra of **3qa**

methyl 2-(isopropylthio)-4-methyl-3-oxo-3,4-dihydroquinoxaline-6-carboxylate (3ra)

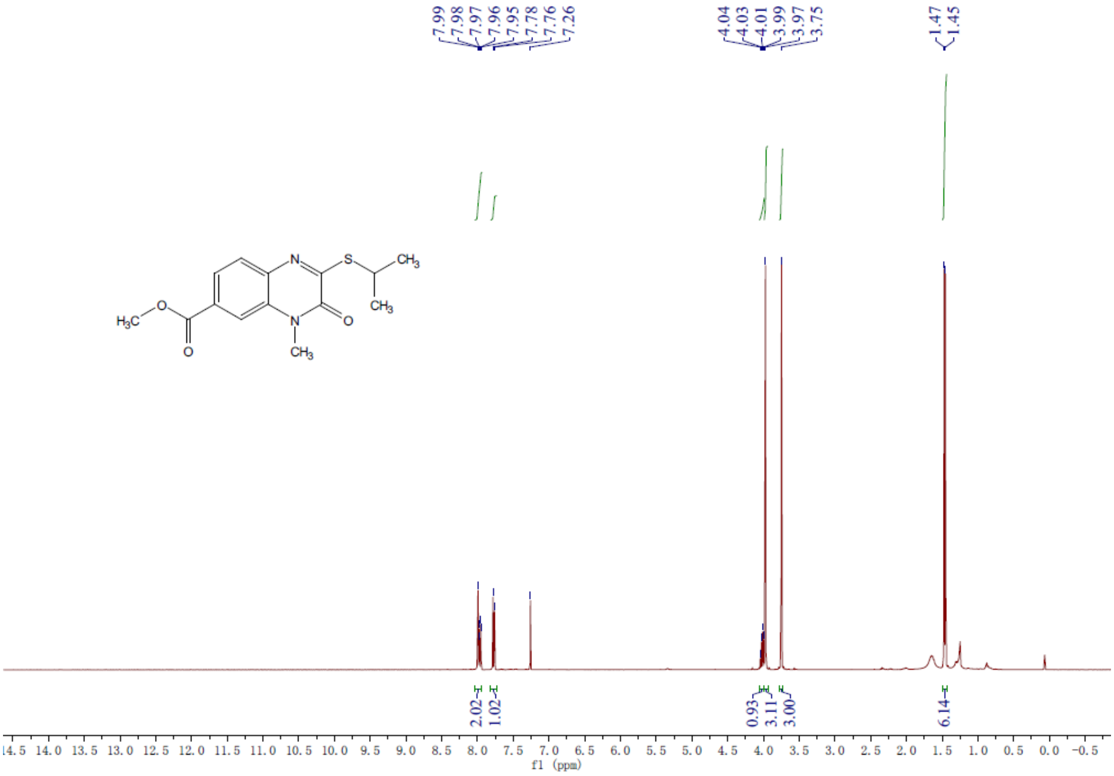

^{1}H spectra of **3ra**

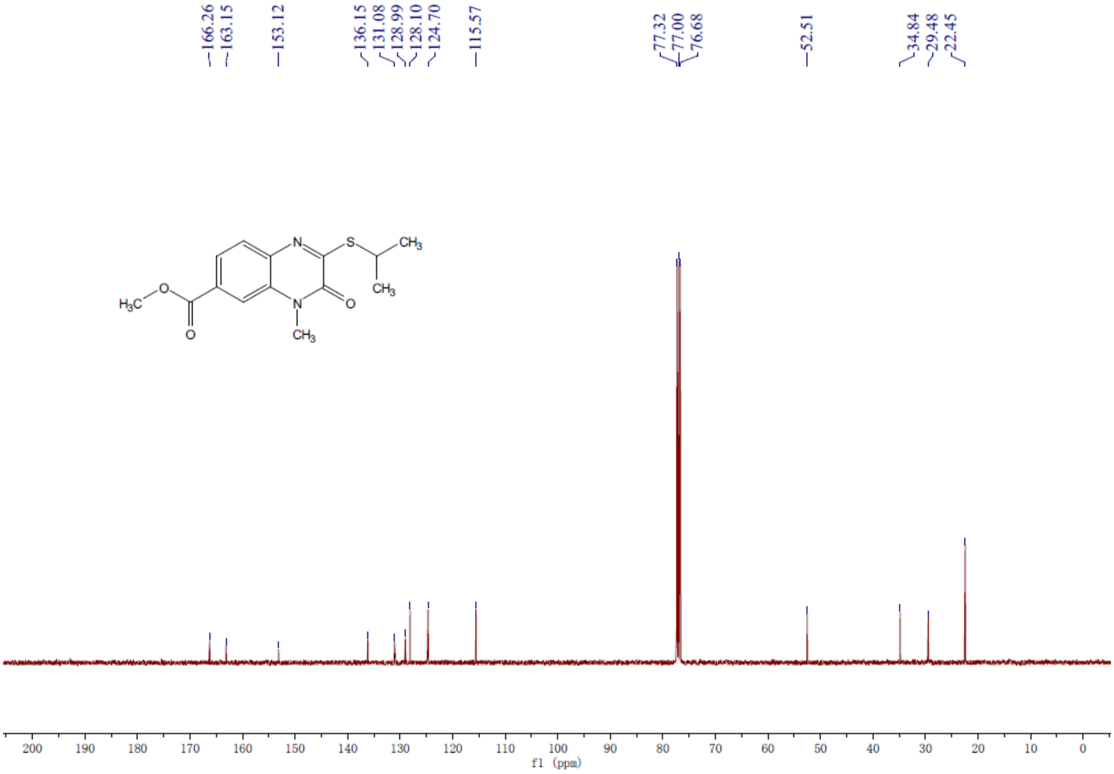

^{13}C spectra of **3ra**

3-(isopropylthio)quinoxalin-2(1*H*)-one (3sa)

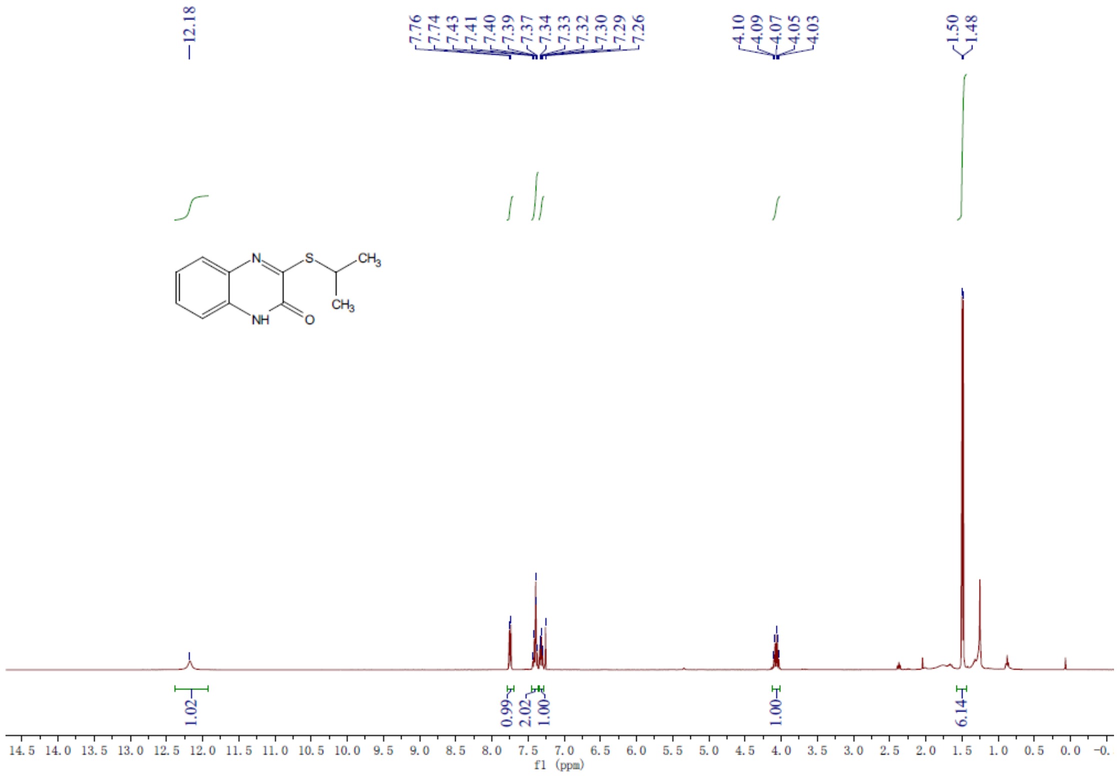

^{1}H spectra of **3sa**

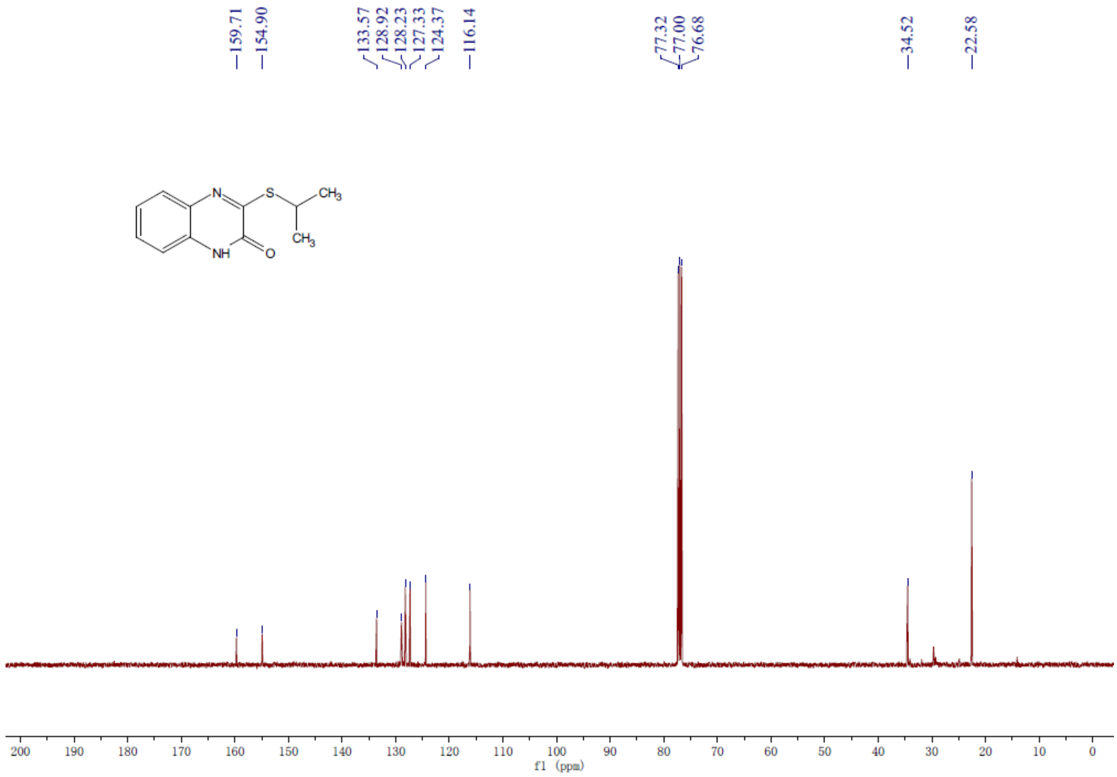

^{13}C spectra of **3sa**

3-(isopropylthio)-1-methylbenzo[g]quinoxalin-2(1*H*)-one (3ta)

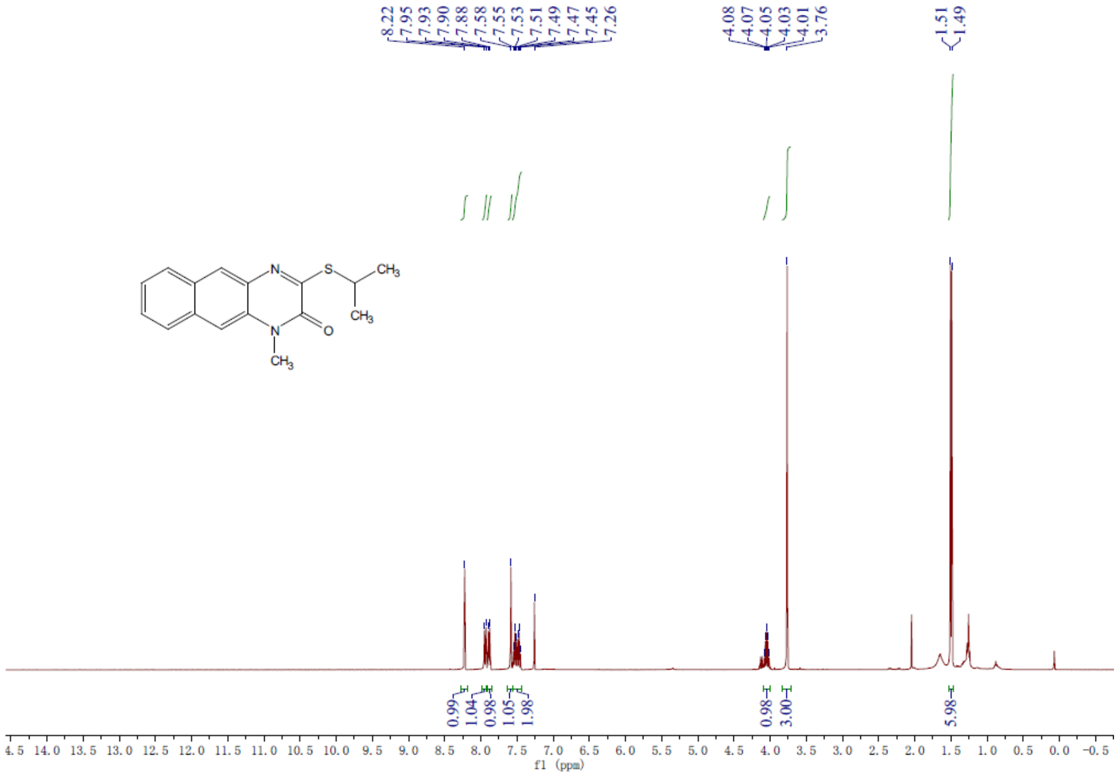

^{1}H spectra of **3ta**

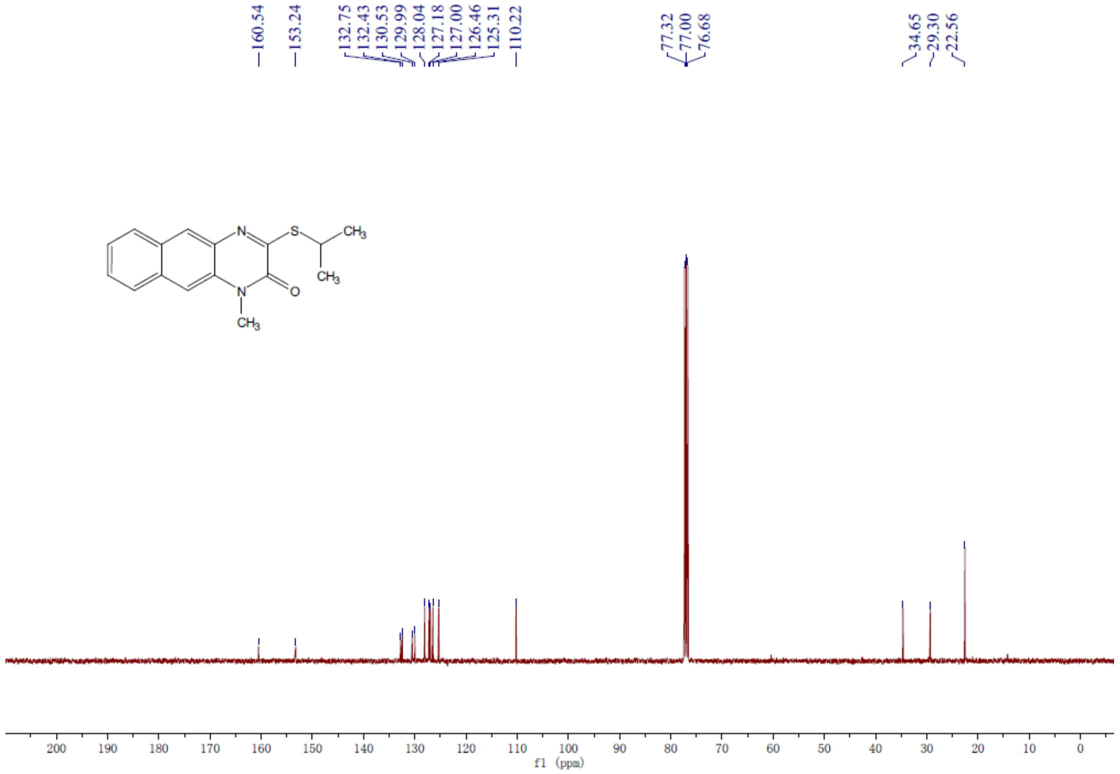

^{13}C spectra of **3ta**

3-(butylthio)-1-methylquinoxalin-2(1*H*)-one (3ab)

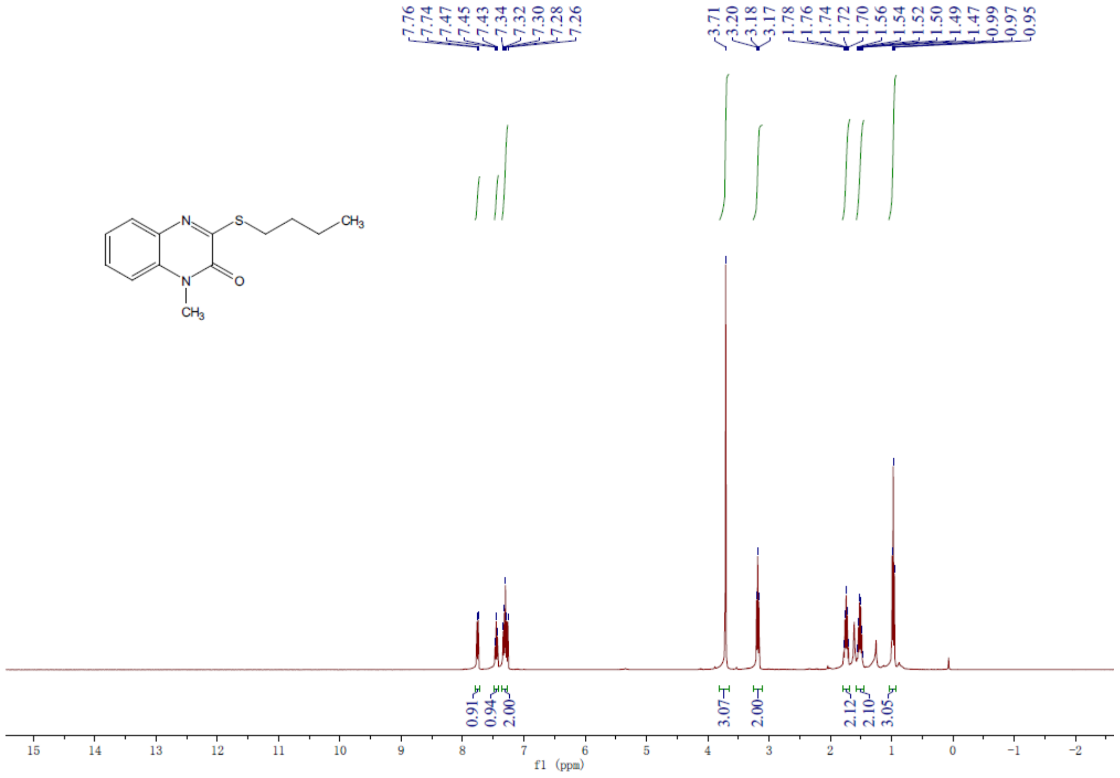

^{1}H spectra of **3ab**

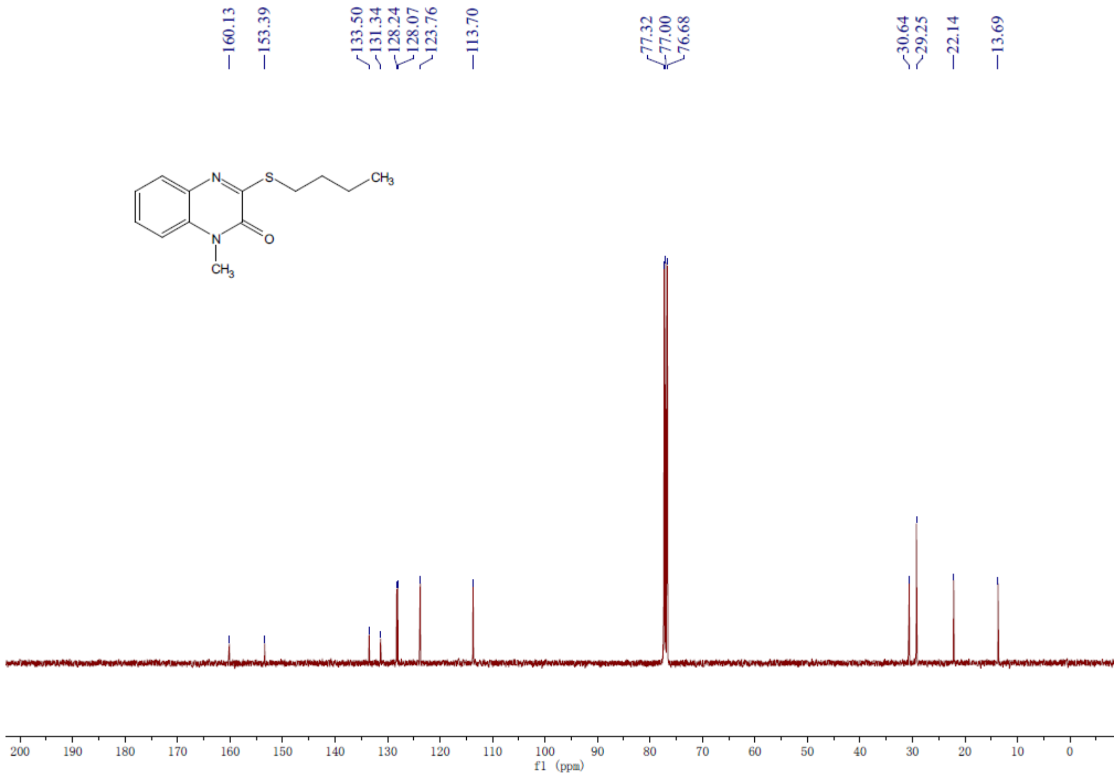

^{13}C spectra of **3ab**

1-methyl-3-(octylthio)quinoxalin-2(1*H*)-one (3ac)

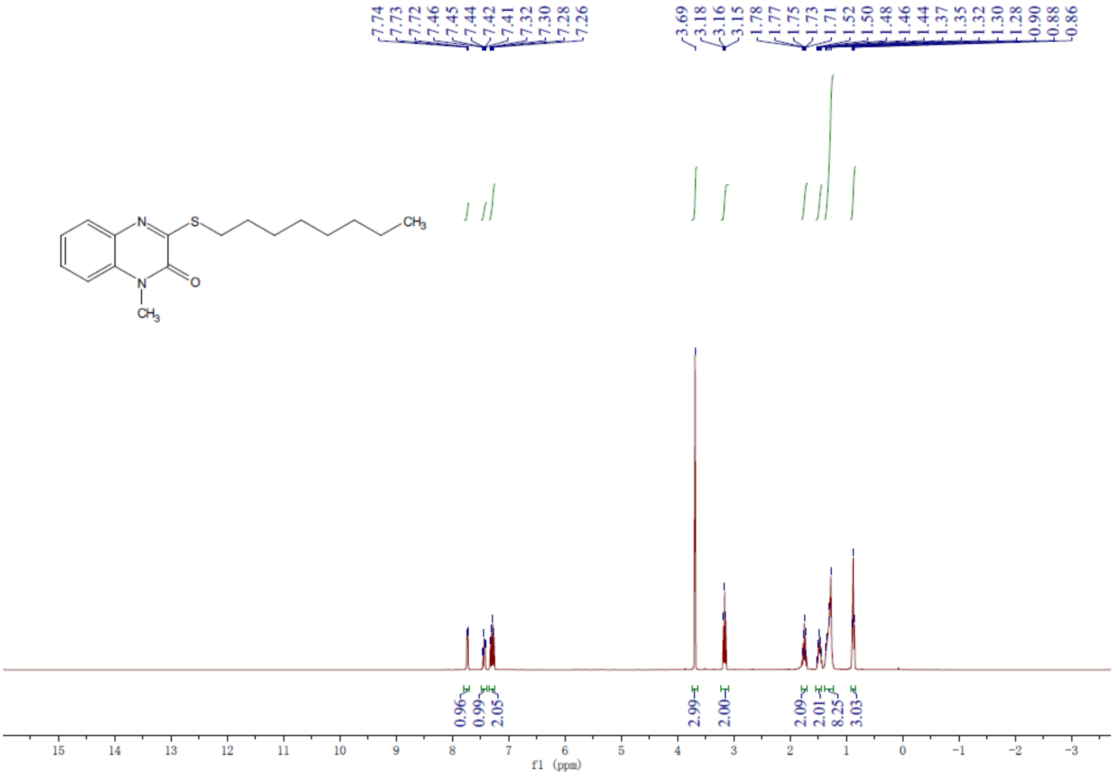

^{1}H spectra of **3ac**

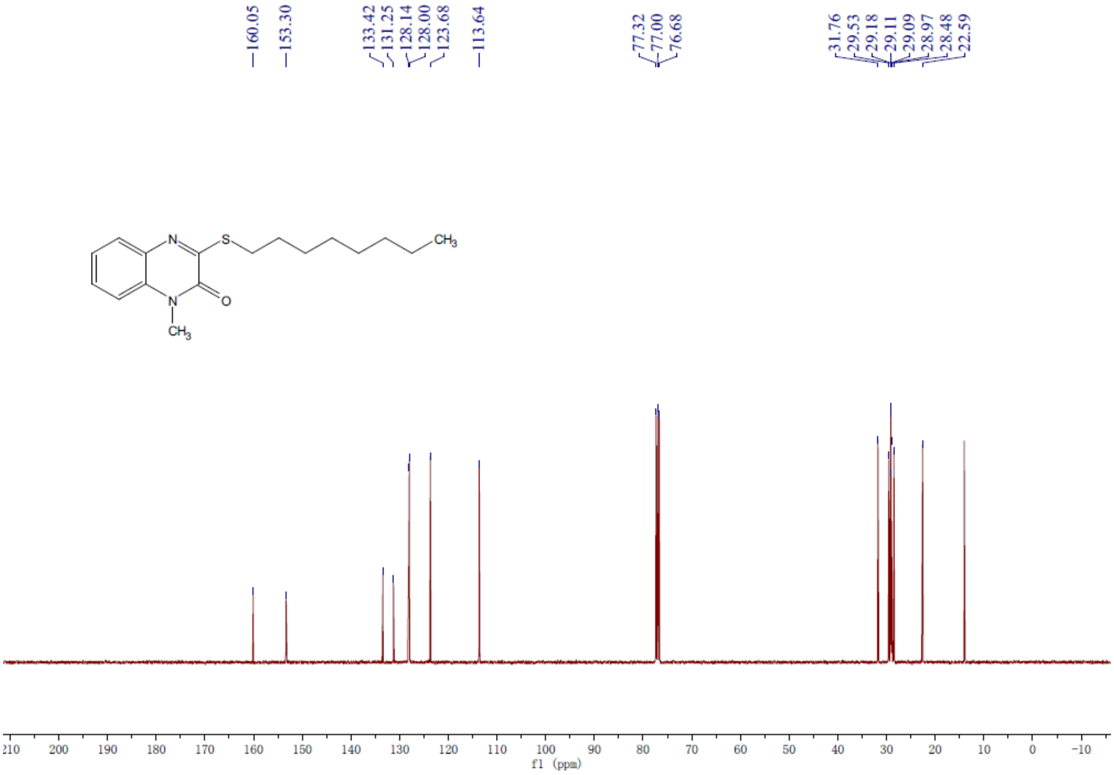

^{13}C spectra of **3ac**

3-(isobutylthio)-1-methylquinoxalin-2(1*H*)-one (3ad)

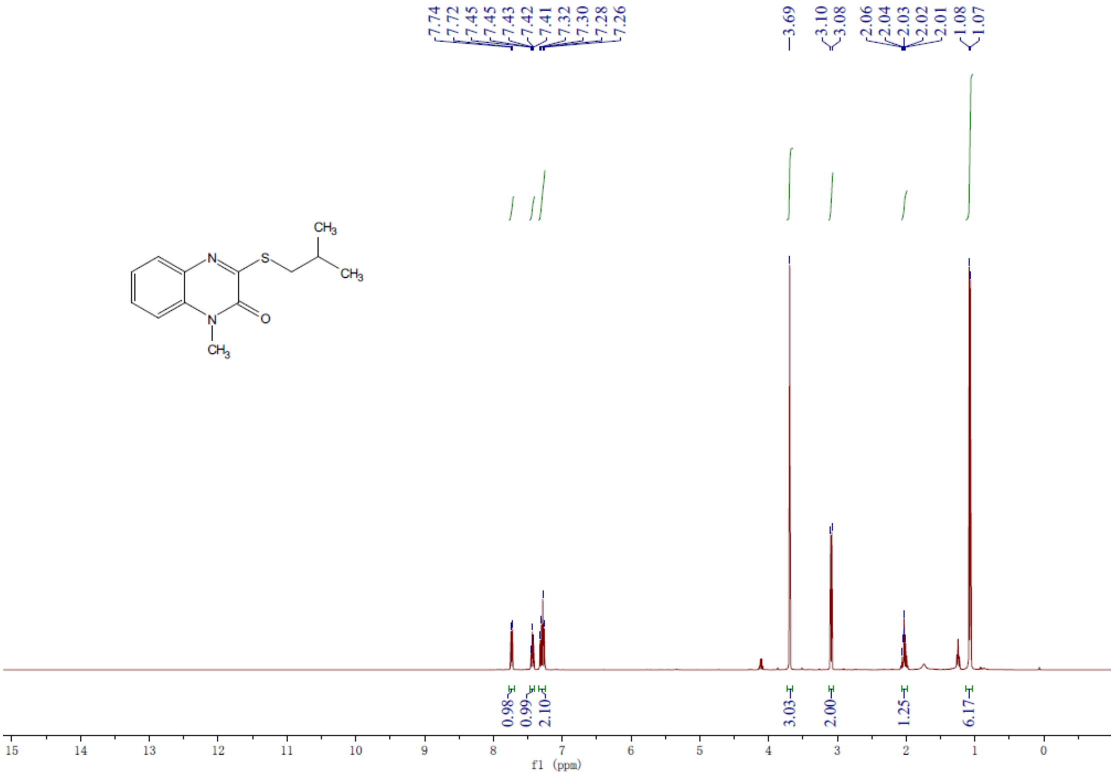

^{1}H spectra of **3ad**

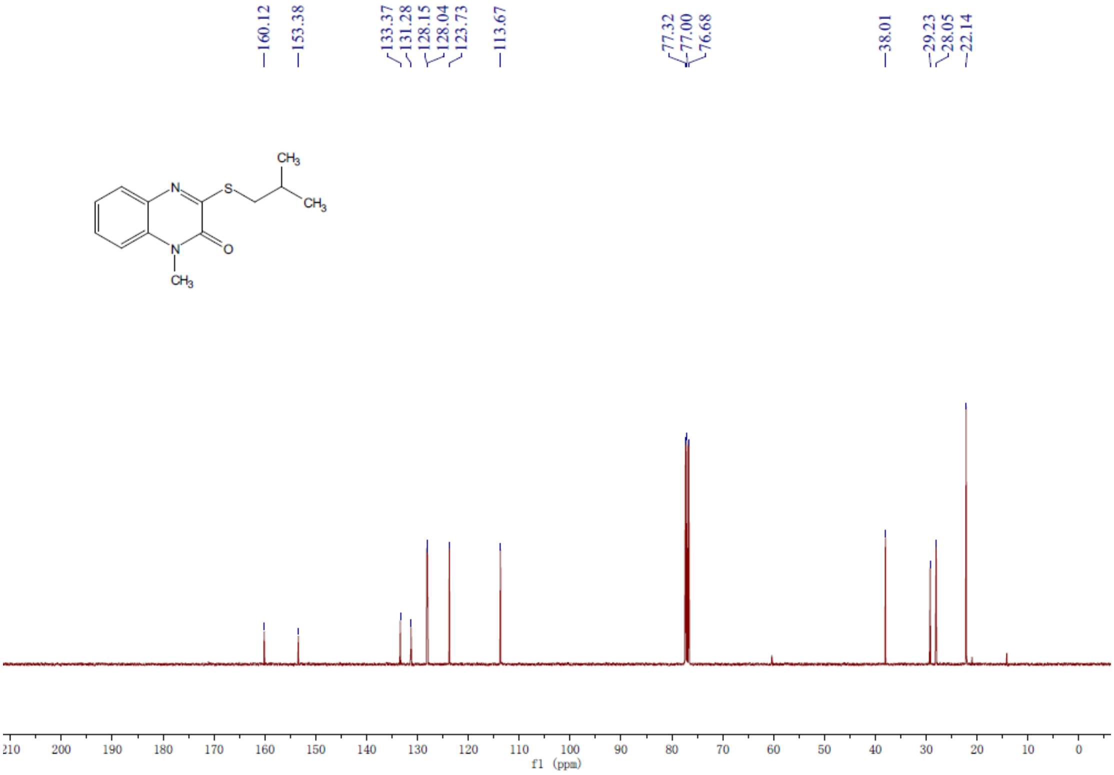

^{13}C spectra of **3ad**

3-(tert-butylthio)-1-methylquinoxalin-2(1*H*)-one (3ae)

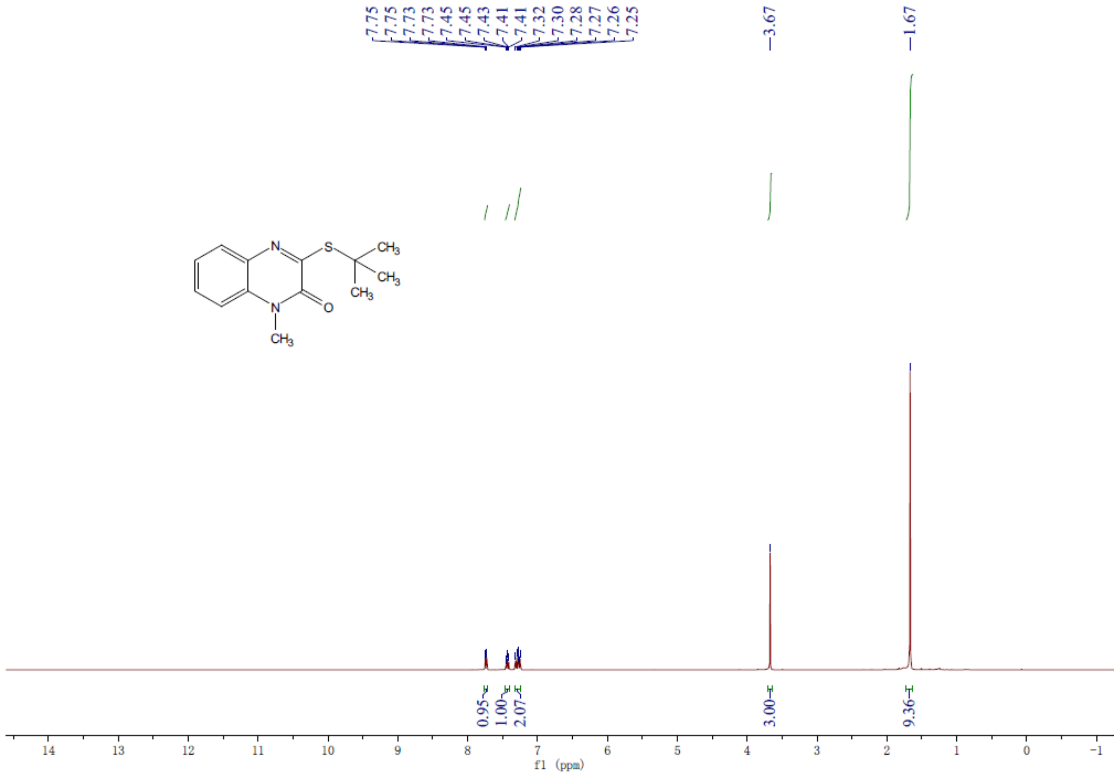

^{1}H spectra of **3ae**

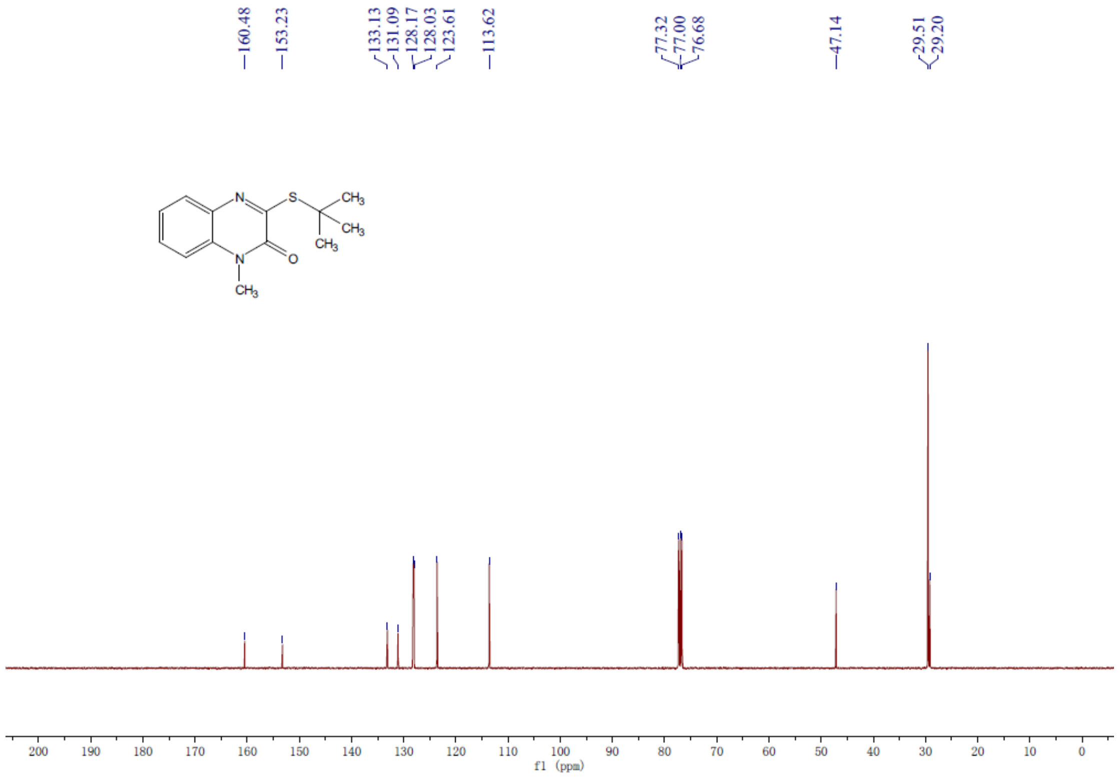

^{13}C spectra of **3ae**

1-methyl-3-((3-methylbutan-2-yl)thio)quinoxalin-2(1*H*)-one (3af)

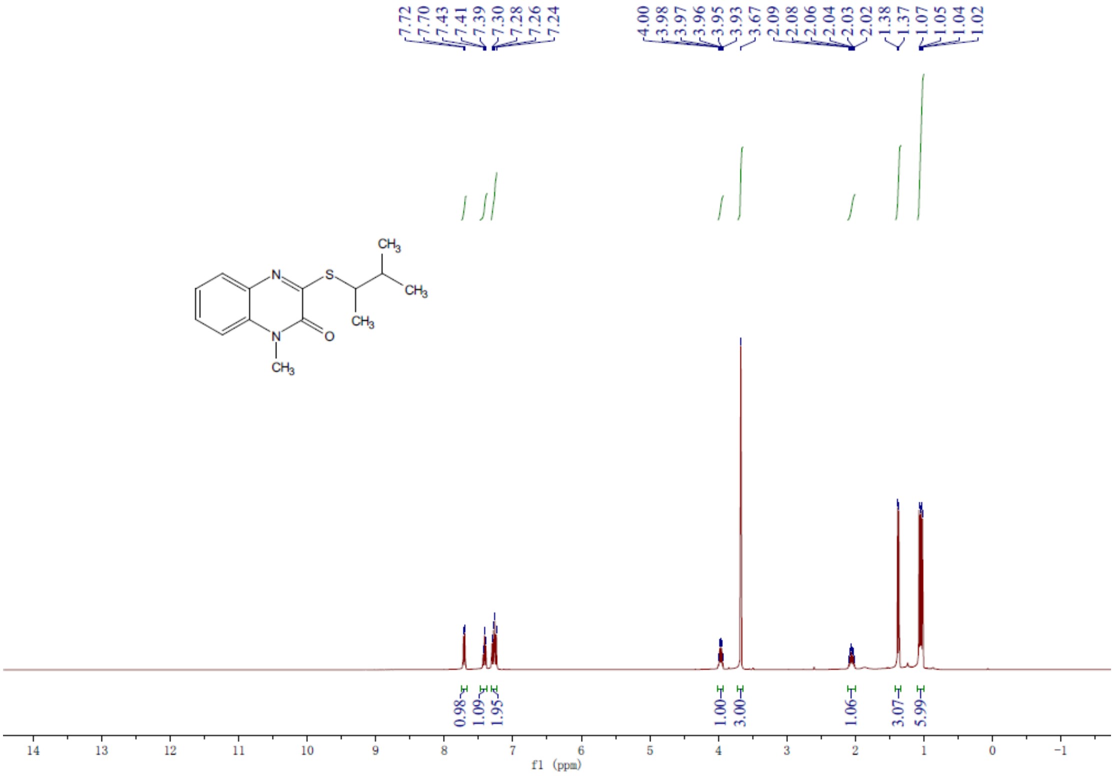

^{1}H spectra of **3af**

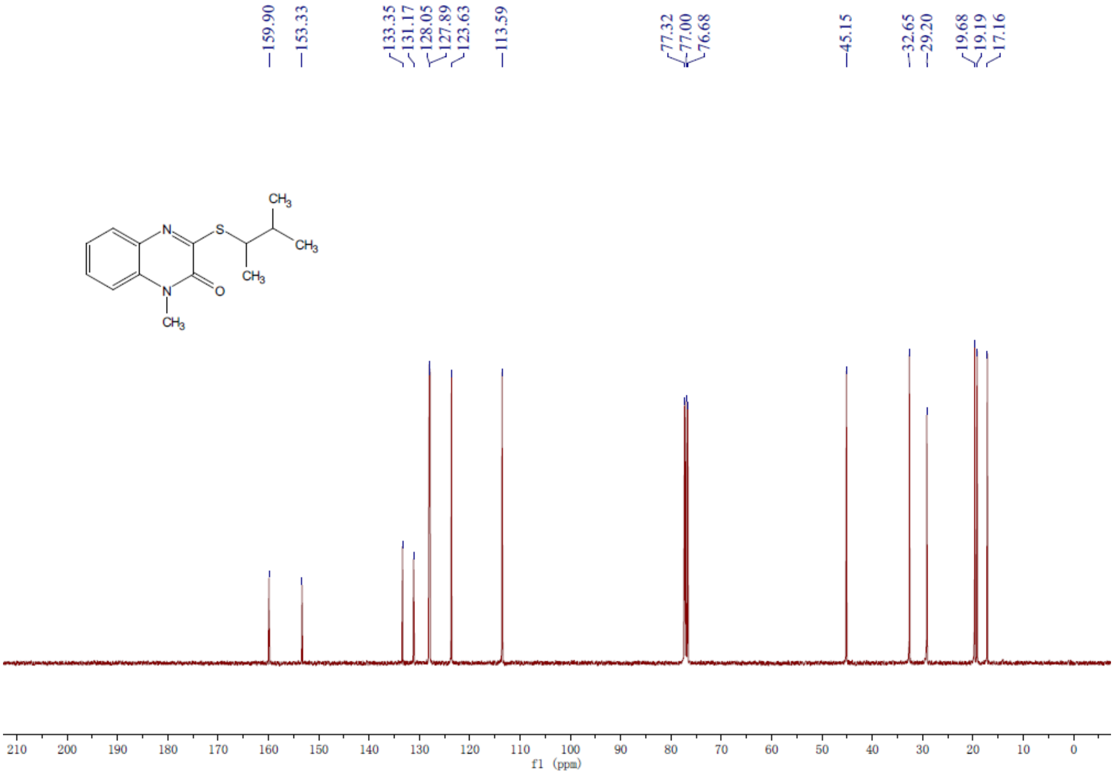

^{13}C spectra of **3af**

3-(benzylthio)-1-methylquinoxalin-2(1*H*)-one (3ag)

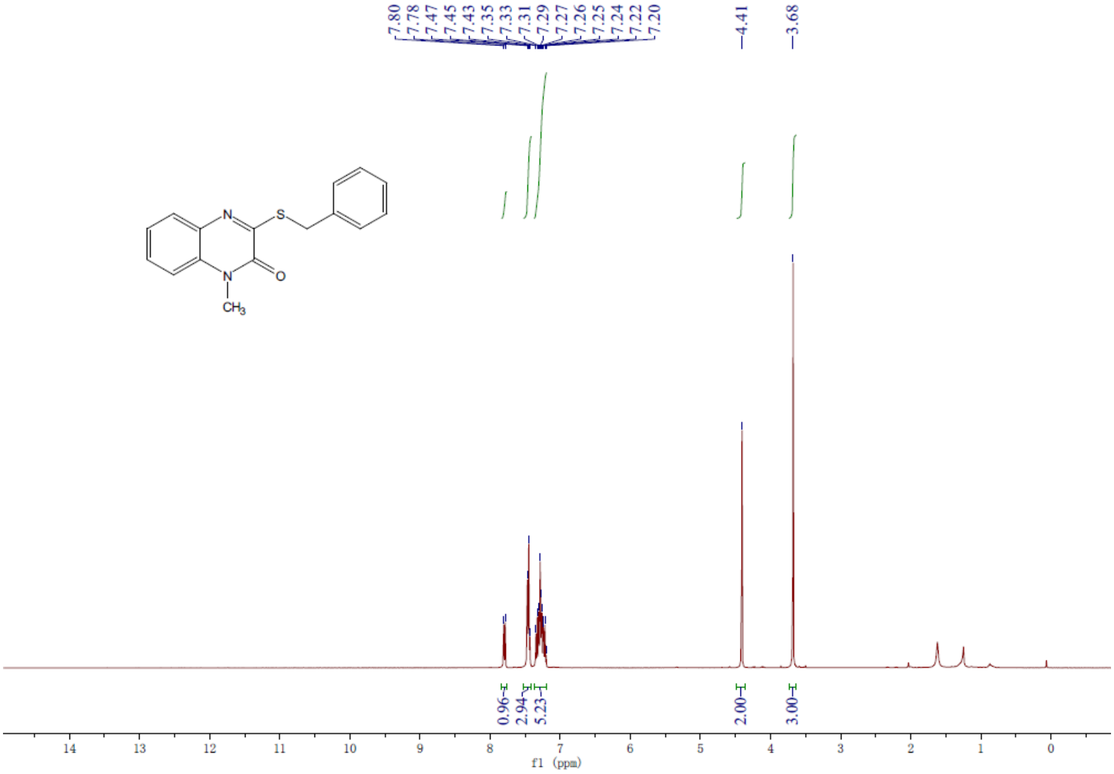

^{1}H spectra of **3ag**

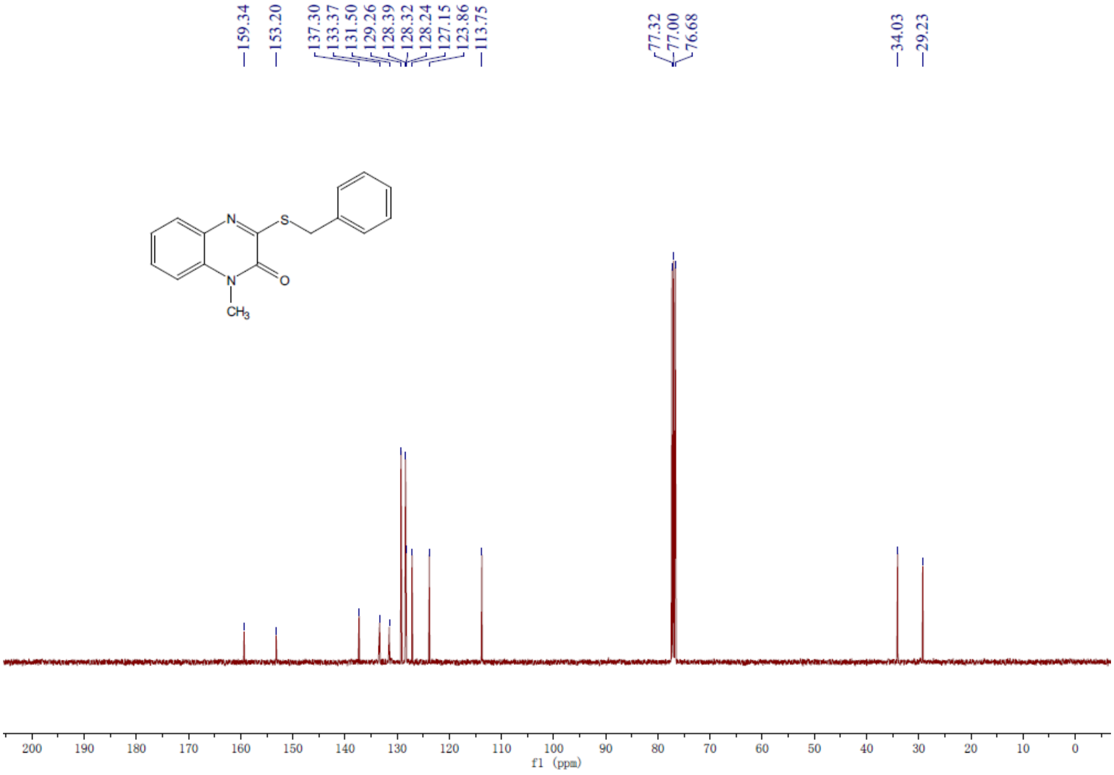

^{13}C spectra of **3ag**

3-((4-chlorobenzyl)thio)-1-methylquinoxalin-2(1*H*)-one (3ah)

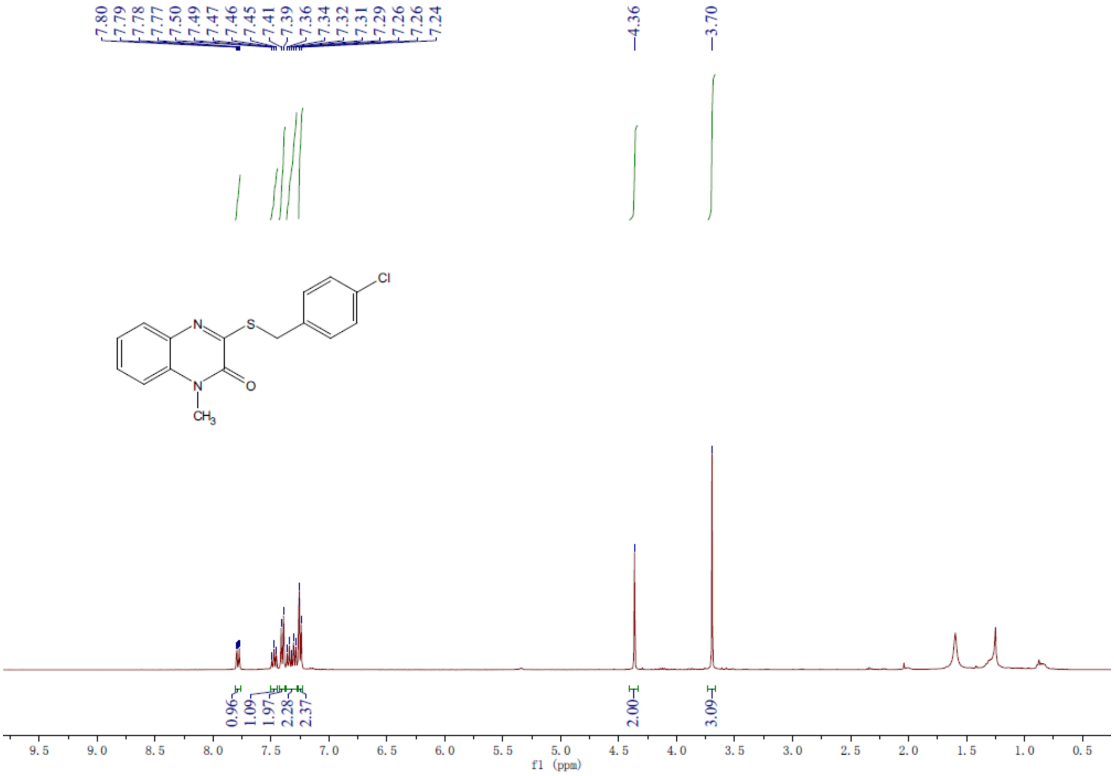

^{1}H spectra of **3ah**

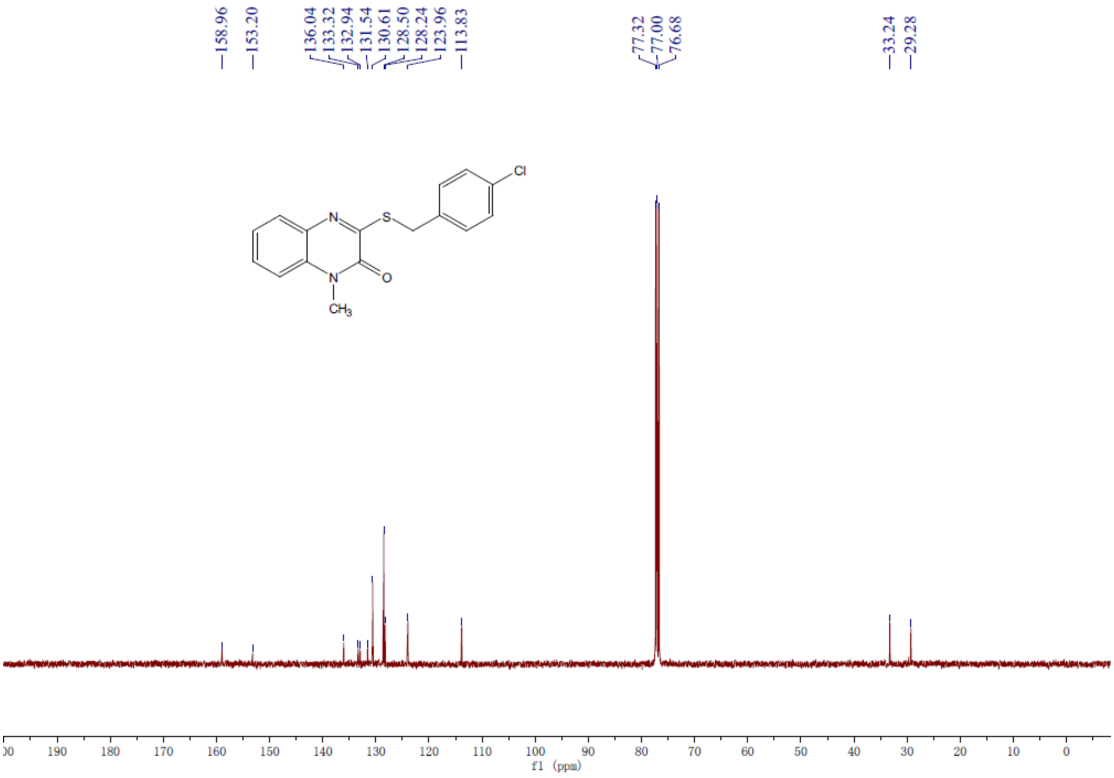

^{13}C spectra of **3ah**

3-((furan-2-ylmethyl)thio)-1-methylquinoxalin-2(1*H*)-one (3ai)

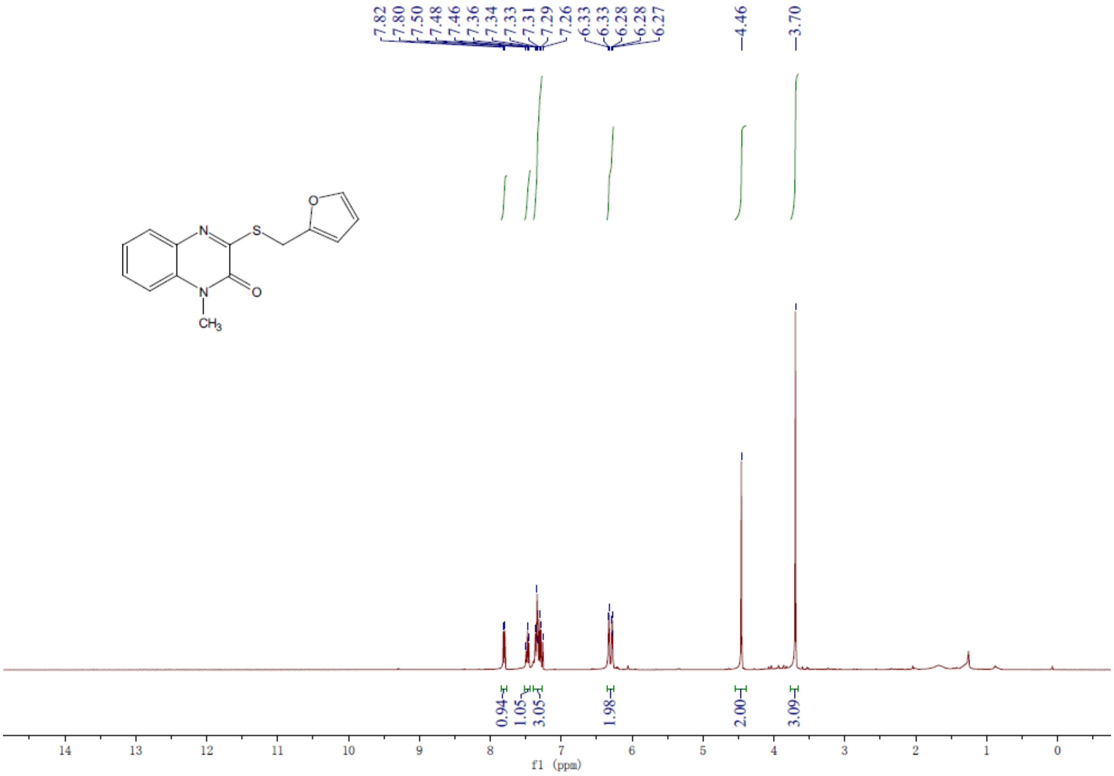

^{1}H spectra of **3ai**

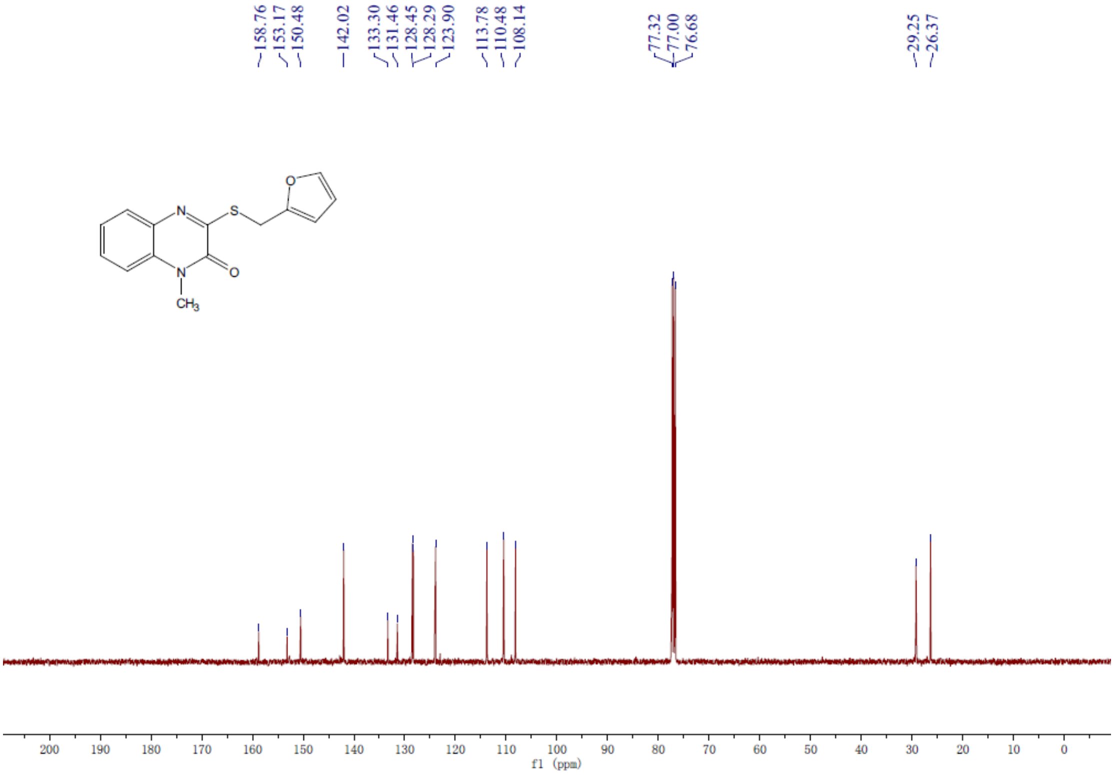

^{13}C spectra of **3ai**

3-(cyclopentylthio)-1-methylquinoxalin-2(1*H*)-one (3aj)

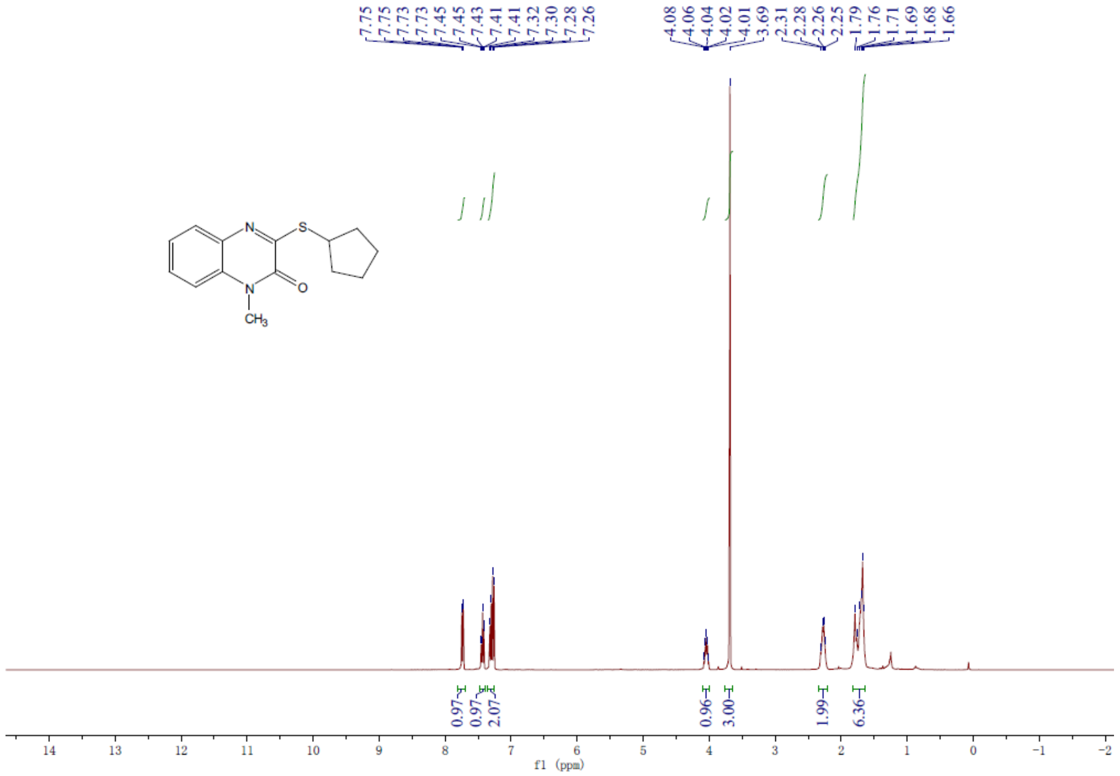

^{1}H spectra of **3aj**

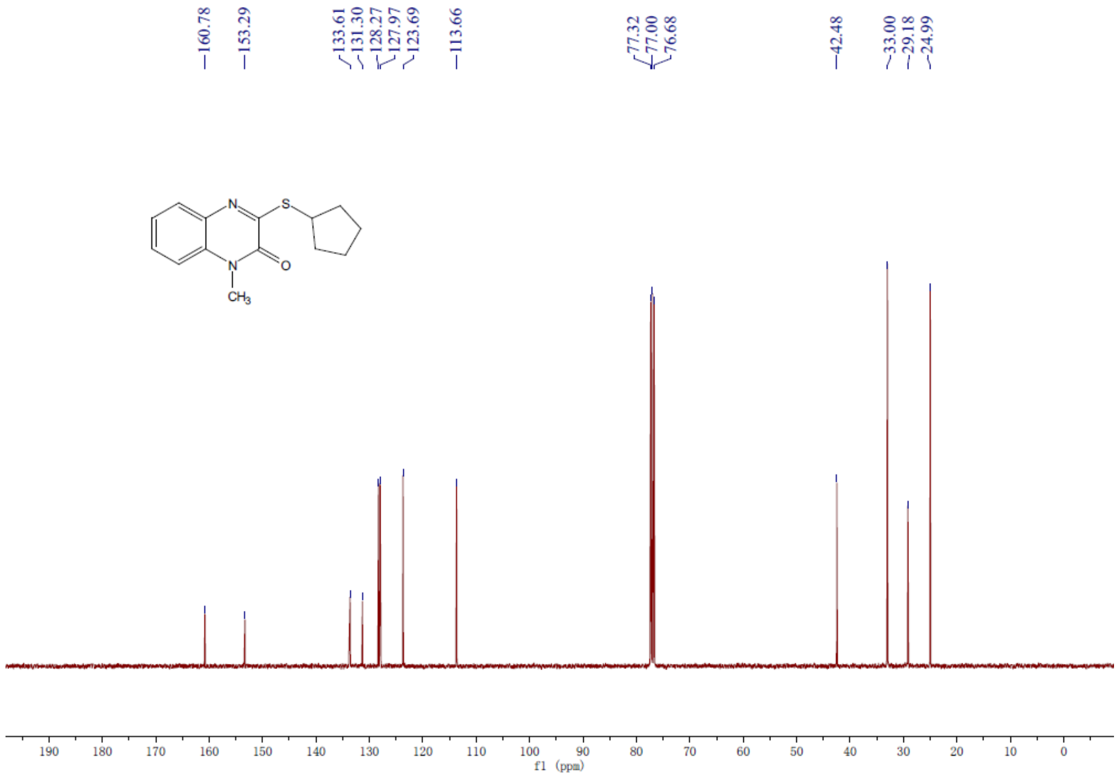

^{13}C spectra of **3aj**

3-(cyclohexylthio)-1-methylquinoxalin-2(1*H*)-one (3ak)

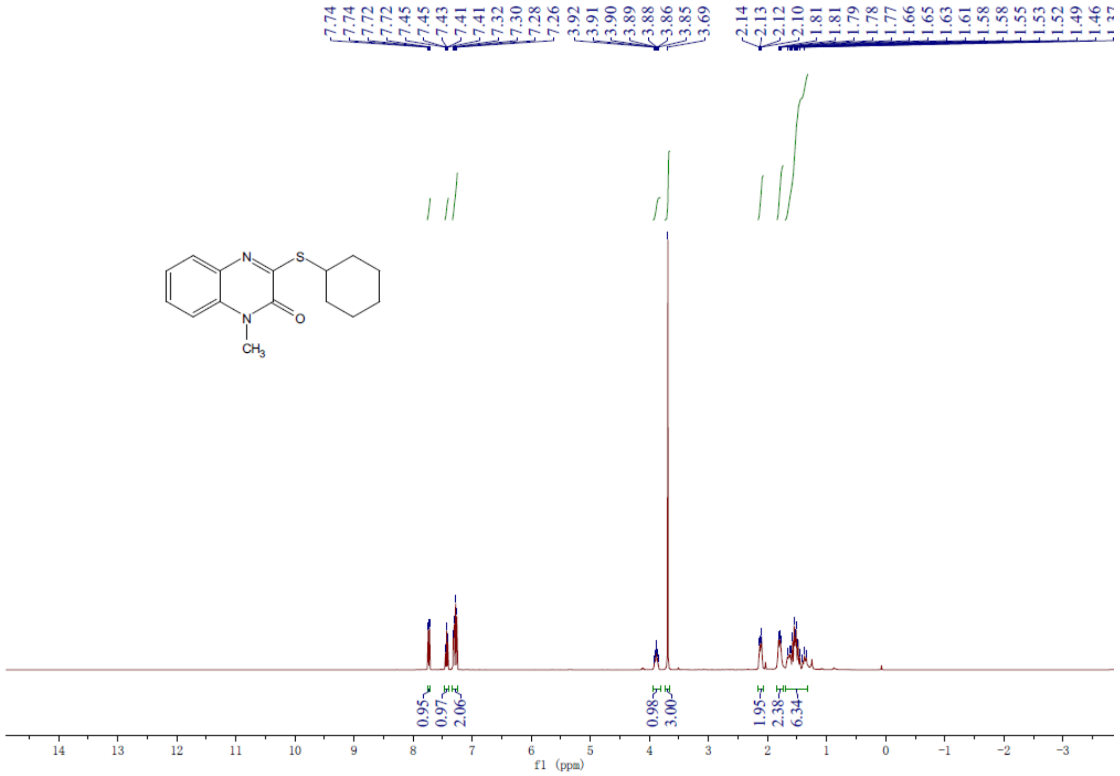

^{1}H spectra of **3ak**

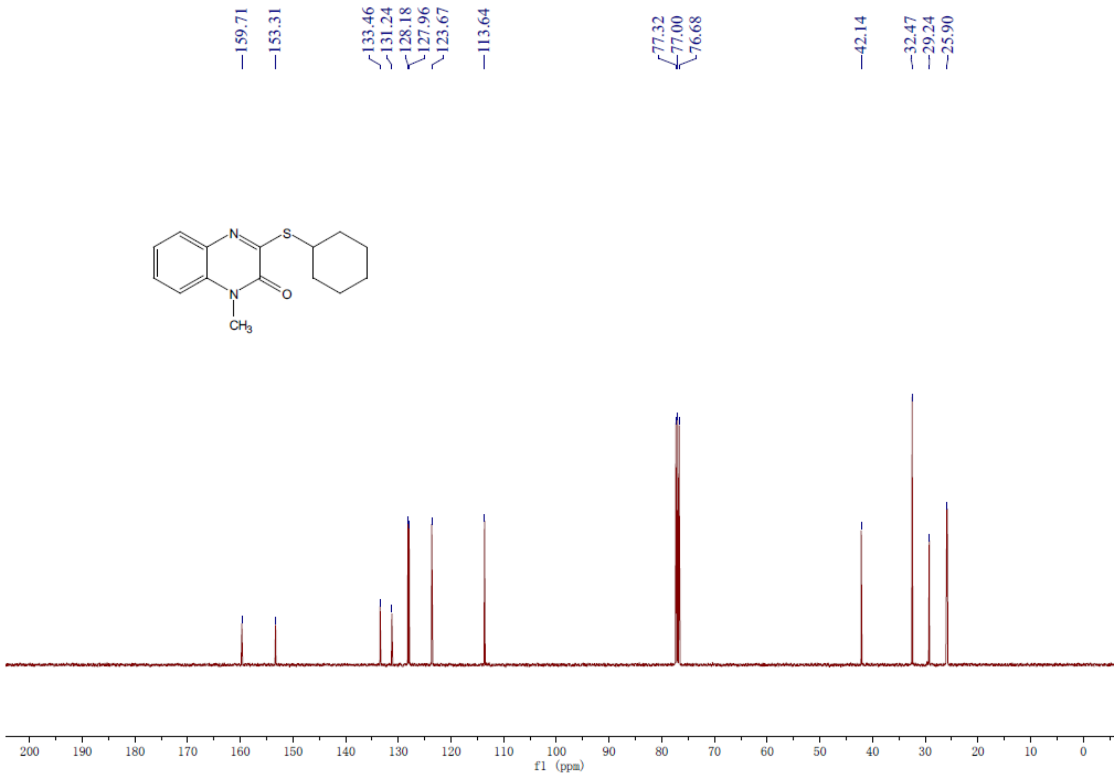

^{13}C spectra of **3ak**

1-methyl-3-((2-methyltetrahydrofuran-3-yl)thio)quinoxalin-2(1*H*)-one (3al)

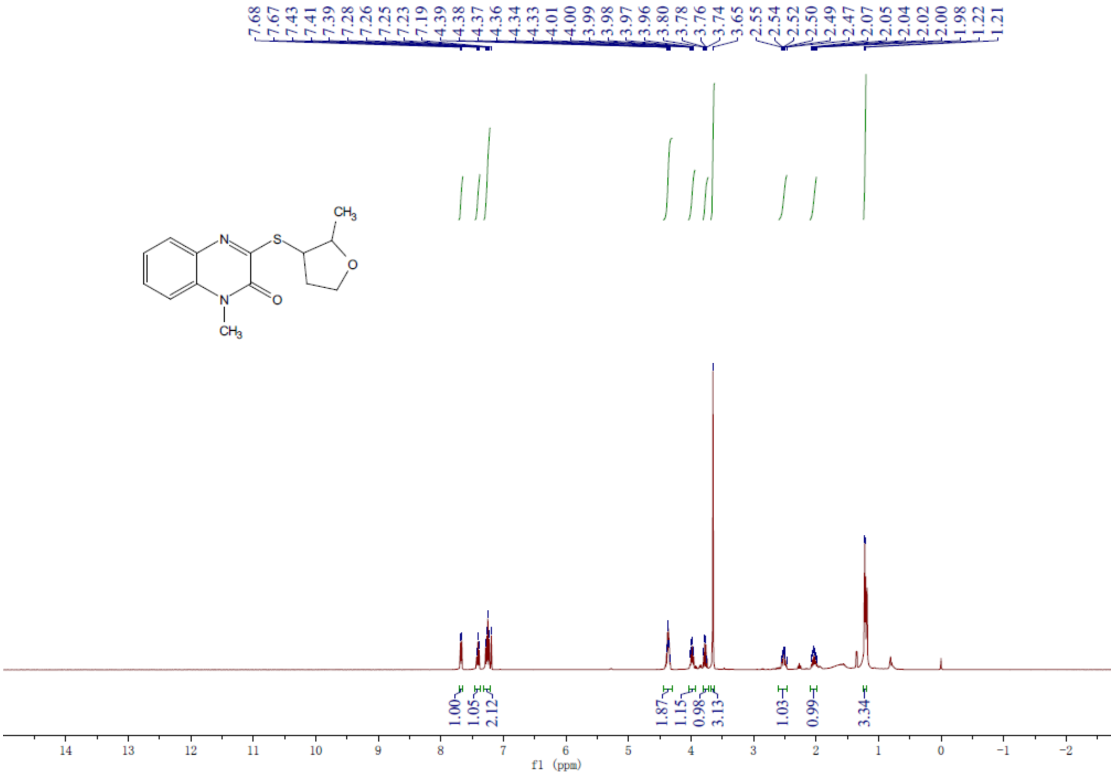

^{1}H spectra of **3al**

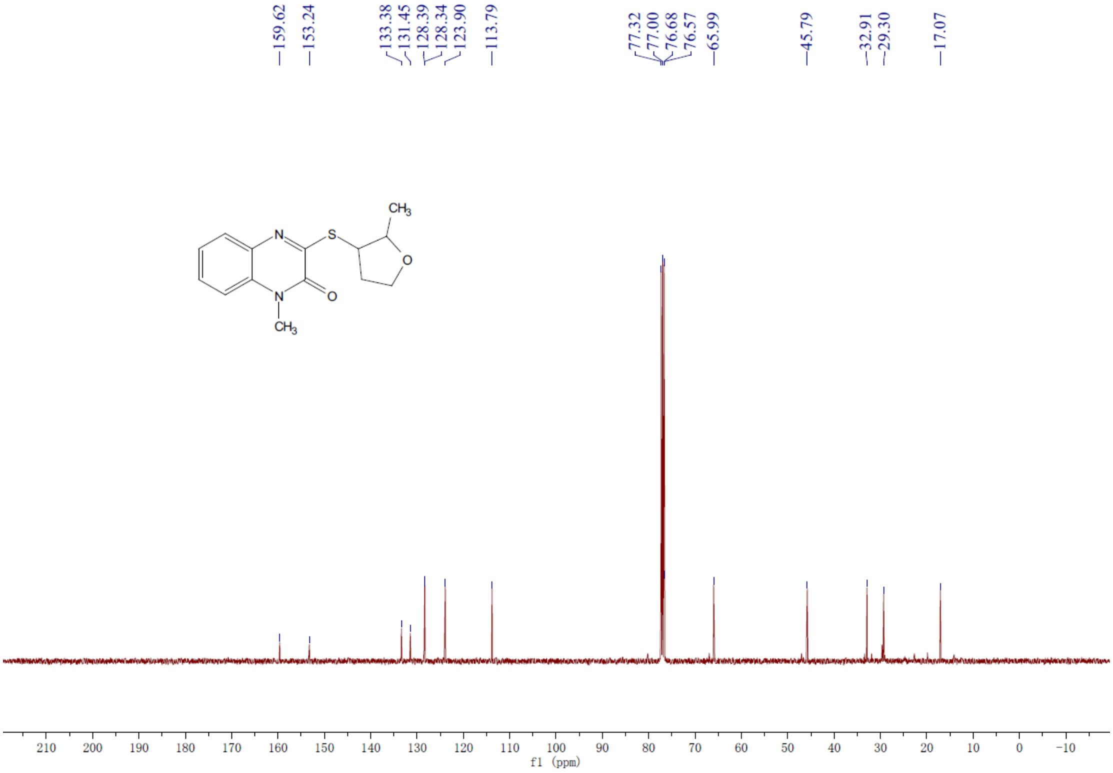

^{13}C spectra of **3al**
